# Supplementary material for: Targeted assembly of ectopic kinetochores to induce chromosome‐specific segmental aneuploidies
Source: EMBO J. 2023 Apr 17;42(10):e111587. doi: 10.15252/embj.2022111587 (PMC10183824; doi:10.15252/embj.2022111587)
Supplement: Supplementary file 2 — Movie EV1 [file EMBJ-42-e111587-s006.zip › EMBOJ-2022-111587R-movie_EV1_legend.docx]

**Movie EV1: CENP-T^∆C^-dCas9-nucleated ectopic kinetochores induce prolonged metaphase, and target chromosome mis-segregation.** Time-lapse movie (8h) from live cell imaging of a HEK293T cell with CENP‑T^∆C^‑dCas9‑EGFP targeted to chromosome 9 (Chr9-CEN). The cell exhibits a prolonged metaphase before mis-segregating the EGFP signal during anaphase. Blue = Hoechst, Green = EGFP. All frames are maximum intensity projections taken across the height of the cell.
