# Supplementary material for: Targeted assembly of ectopic kinetochores to induce chromosome‐specific segmental aneuploidies
Source: EMBO J. 2023 Apr 17;42(10):e111587. doi: 10.15252/embj.2022111587 (PMC10183824; doi:10.15252/embj.2022111587)
Supplement: Supplementary file 3 — Movie EV2 [file EMBJ-42-e111587-s003.zip › EMBOJ-2022-111587R-movie_EV2_legend.docx]

**Movie EV2: CENP-T^∆C^-dCas9 target chromosomes mis-segregated under Mps1 inhibition are often unequally distributed between daughter cells.** Time-lapse movie (4h) from live cell imaging of a HEK293T cell expressing H2B-RFP with CENP‑T^∆C^‑dCas9‑EGFP targeted to chromosome 9 (Chr9-CEN) after Mps1i treatment. The cell mis-segregates the EGFP signal during anaphase, and fails to resolve the error in telophase leaving an unresolved bridge in the formed daughter cells. This bridge resolves, breaking into 2 EGFP spots to leave the daughter cells with an unequal distribution of EGFP signals (4:3). Magenta = H2B-RFP, Green = EGFP. All frames are maximum intensity projections taken across the height of the cell.
