# Supplementary material for: Targeted assembly of ectopic kinetochores to induce chromosome‐specific segmental aneuploidies
Source: EMBO J. 2023 Apr 17;42(10):e111587. doi: 10.15252/embj.2022111587 (PMC10183824; doi:10.15252/embj.2022111587)
Supplement: Supplementary file 4 — Dataset EV1 [file EMBJ-42-e111587-s004.pdf]

Expanded View Figures

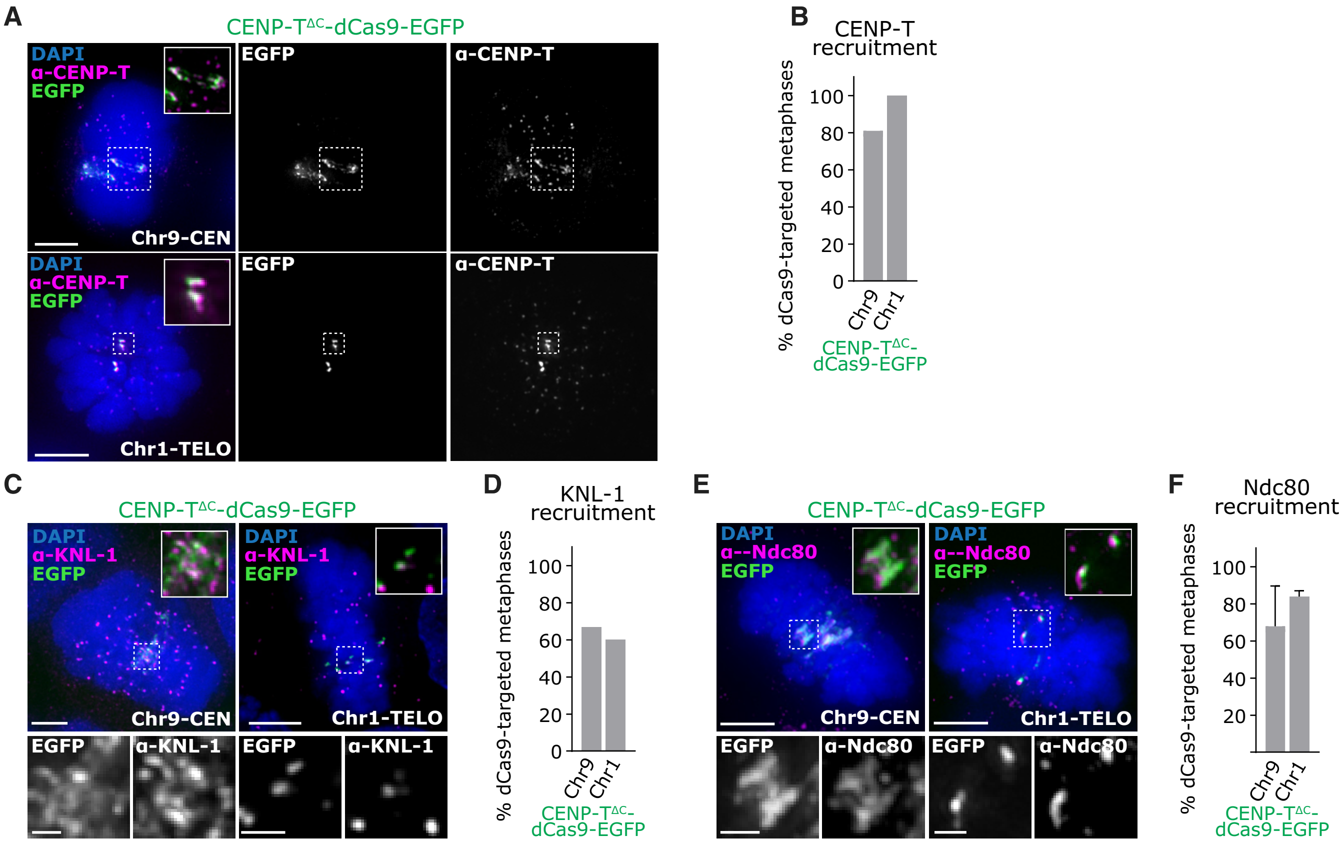

Figure EV1. related to Fig 2. CENP-T$^{\Delta C}$-dCas9 targeting recruits KNL-1 and Ndc80 to large repetitive chromosomal loci in HCT116 cells.

A Immunofluorescence images of HCT116 cells with CENP-T$^{\Delta C}$-dCas9-EGFP targeted to Chr9-CEN or Chr1-TELO, stained with antibodies against CENP-T.

B Percentage of metaphase cells showing EGFP and CENP-T signal co-localisation. ≥ 20 metaphases analysed per condition, 1 experiment.

C Immunofluorescence images of HCT116 cells with CENP-T$^{\Delta C}$-dCas9-EGFP targeted to Chr9-CEN or Chr1-TELO, stained with antibodies against KNL-1.

D Percentage of metaphase cells showing EGFP and KNL-1 signal co-localisation. ≥ 20 metaphases analysed per condition, 1 experiment.

E Immunofluorescence images of HCT116 cells with CENP-T$^{\Delta C}$-dCas9-EGFP targeted to Chr9-CEN or Chr1-TELO, stained with antibodies against Ndc80.

F Percentage of metaphase cells showing colocalization of EGFP with Ndc80. ≥ 20 metaphases counted per condition, in each of 2 experiments, Bars = mean ± SD.

Data information: Images in (A, C, E) are maximum intensity projections taken across the depth of the EGFP foci. Scale bars = 5 µm on large images, 1 µm on zooms.

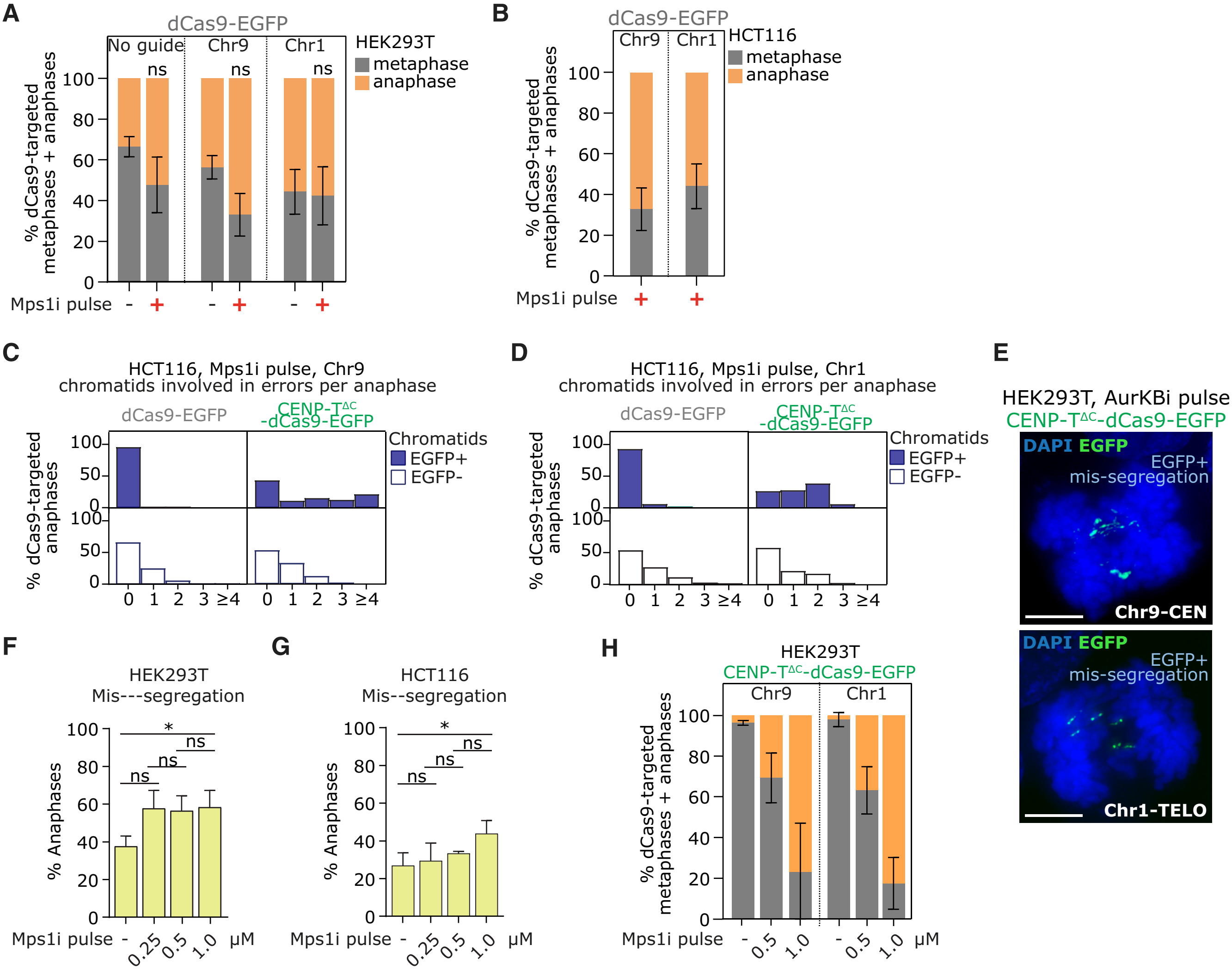

Figure EV2. related to Fig 5. Lowering Mps1 inhibitor dose does not improve off-target chromosome mis-segregation.

A, B Quantification of mitotic stage from fixed dCas9-EGFP targeted HEK293T (A) or HCT116 (B) cells following Mps1i pulse.

C, D Quantification of number of EGFP+ or EGFP- chromatids involved in segregation errors from HCT116 anaphases with dCas9 targeting.

E Immunofluorescence images of anaphase HEK293T cells with CENP-TΔC-dCas9 EGFP targeting after 30 min AurKBi treatment, showing examples of EGFP+ mis segregation. Maximum intensity projection taken across the depth of the EGFP foci. Scale bars = 5 µm.

F, G Mis-segregation rate in untransfected HEK293T (F) or HCT116 (G) cells following Mps1i pulse at 0.25, 0.5 or 1 µM. $^{ns}P > 0.05$, $^{*}P < 0.05$ (One way ANOVA with Šidák's multiple comparison correction).

H Quantification of mitotic stage from fixed CENP $T^{\Delta C}$-dCas9-EGFP targeted HEK293T cells after a pulse or Mps1i at 0.5 or 1 µM. ≥ 50 metaphases + anaphases analysed per condition, in each of 2 experiments.

Data information: Data in (A, B, H) are from ≥ 40 metaphase + anaphases per condition, in each of 3 experiments for (A, B) and 2 experiments for (H). $^{ns}P > 0.05$ (One-way ANOVA with Šidák's multiple comparison correction). Bars = mean ± SD. Data in (C, D, F, G) are from ≥ 25 anaphases analysed per condition, in each of ≥ 3 experiments. In (F, G) Bars = mean + SD.

 © 2023 The Authors

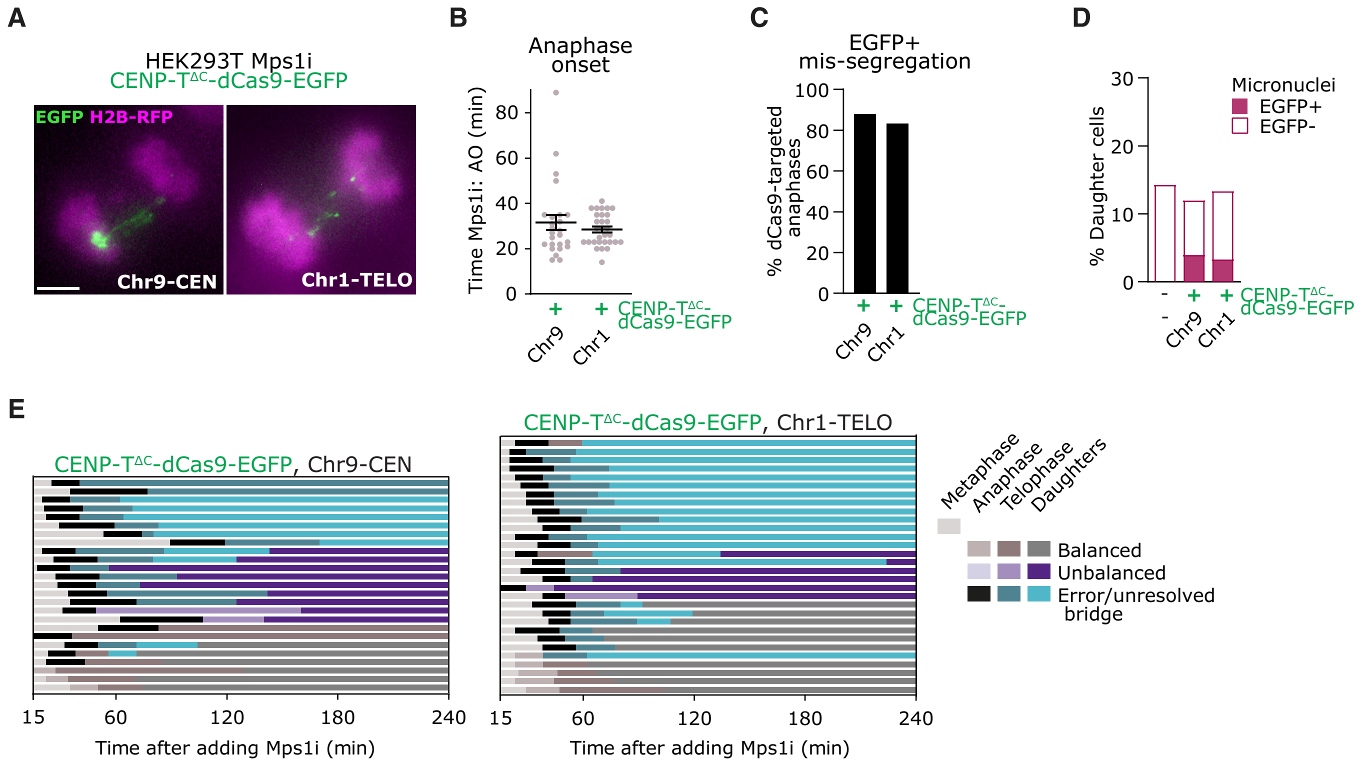

Figure EV3. related to Fig 5. Tracking the fate of mis-segregated target chromosomes by live cell imaging.

A Frames from live cell imaging of HEK293T H2B-RFP cells after treatment with Mps1i, with CENP-T$^{\Delta C}$-dCas9-EGFP targeted to Chr9-CEN or Chr1-TELO, showing examples of EGFP+ segregation errors. Scale bar = 5 μm.

B Time from addition of Mps1i to anaphase onset (AO), for CENPT$^{\Delta C}$dCas9-EGFP targeted HEK293T H2B-RFP cells that were in metaphase when Mps1i was added. Lines = mean ± SEM. Each point = 1 cell.

C Mis-segregation rate in CENP-T$^{\Delta C}$-dCas9-EGFP targeted HEK293T H2B-RFP daughter cells assessed by live cell imaging.

D Percentage of daughter cells with micronuclei, classified as EGFP+ or EGFP-.

E Cell cycle stages of cells with CENP-T$^{\Delta C}$-dCas9-EGFP targeting, by live-cell imaging (each line = 1 cell). Cell cycle stages have been sub-categorised based on EGFP state, ie. balanced/unbalanced distribution between daughter cells/anaphase or telophase fronts, and involvement in errors or unresolved bridges.

Data information: Data in (B, C, D, E) are from ≥ 25 cells tracked per condition, compiled from ≥3 independent experiments.

Figure EV4. related to Fig 6. Additional data from single cell sequencing experiments.

A Heatmap showing copy number alterations (CNAs) in single HEK293T cells treated with Mps1i. Heterogeneity is seen at the whole, and partial chromosome level between cells. Median ploidy was calculated across the genome to produce a median karyotype (lower heatmap).

B, C CNA pileups indicating the CNAs present in each condition, after filtering out any clonal or subclonal CNAs present in control conditions. Copy number gains are indicated in red (pale red = +1, red = +2, dark red = +3 copies above median reference) and losses in blue (pale blue = −1, blue = −2, dark blue = −3 copies below reference). Orange lines indicate centromeres and green lines indicate CENP-T$^{\Delta C}$ target sites.

D Nonclonal large CNAs (> 20 Mb) calculated per chromosome per cell for each condition indicated. Points shown for no guide condition = CNA rates after filtering sub-clonal events from the 2 different control sets.

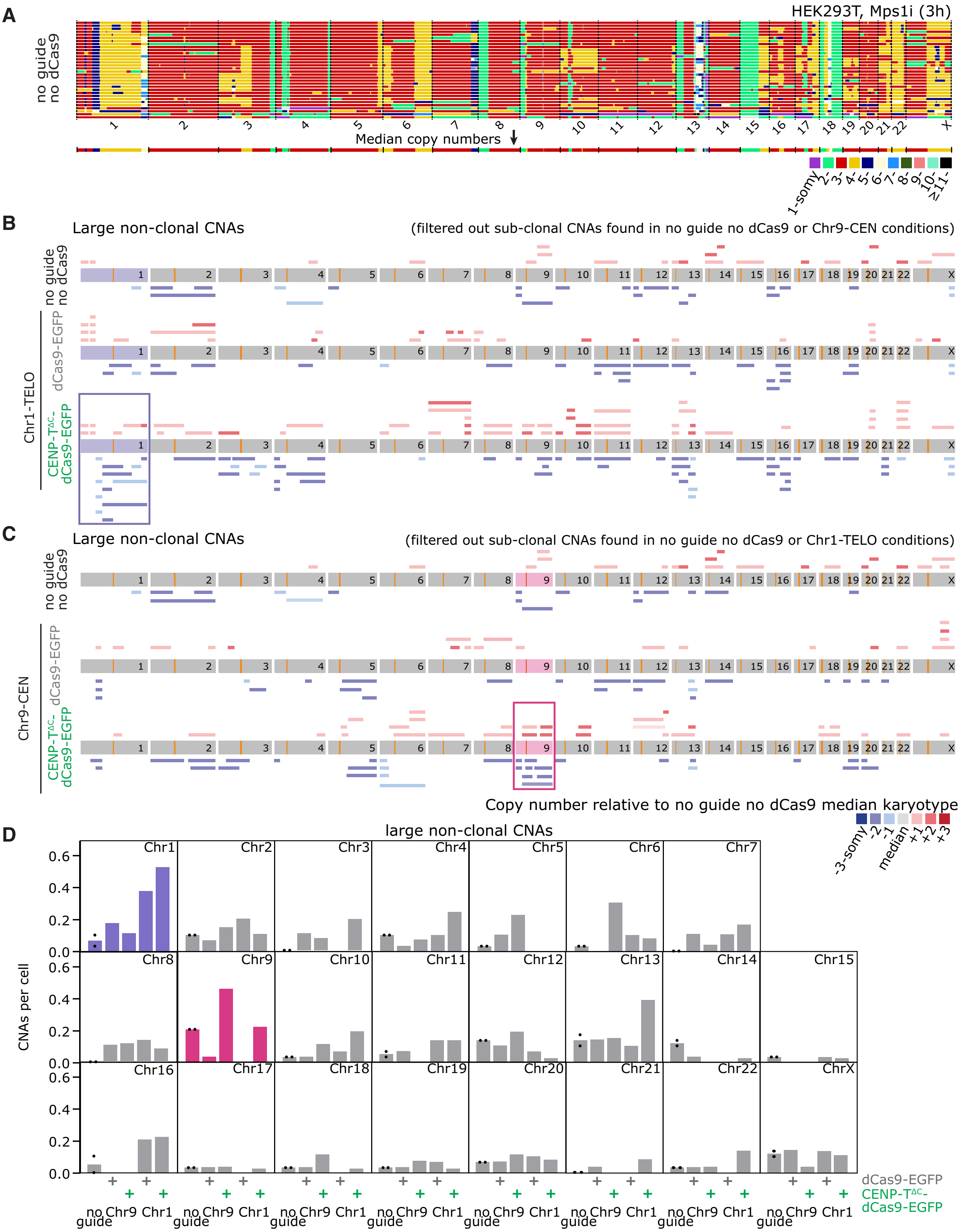

Figure EV4.

 © 2023 The Authors

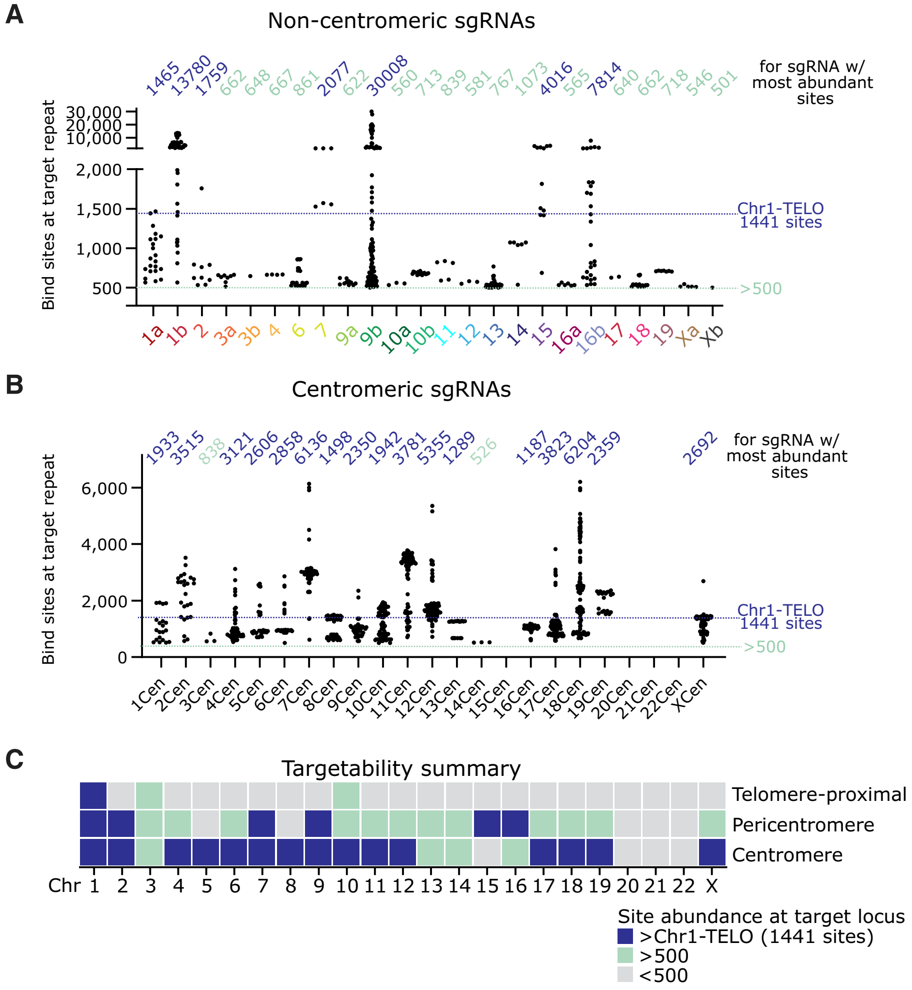

Figure EV5. related to Fig 7. Further details of putative guide RNAs for targeting repeats, including centromeres.

A, B Number of predicted binding sites at the target locus for each guide RNA. Each point = 1 guide RNA. Guides have been grouped by their target repeat and separated into those targeting noncentromeric (A) or centromeric (B) repeats. Numbers at the top of the plots = predicted binding sites at the target repeat for the guide RNA with the highest number of binding sites. Nomenclature for repeats is Cen = centromere, a, b = noncentromeric starting alphabetically at p-telomere.

C Heatmap summarising the identified target repeats for each chromosome, coloured by site abundance for the guide RNA that has the highest number of binding sites. Noncentromeric repeats have been further subcategorised into those proximal to the centromere (Pericentromere) or telomere (Telomere-proximal).

© 2023 The Authors
