# Supplementary material for: Targeted assembly of ectopic kinetochores to induce chromosome‐specific segmental aneuploidies
Source: EMBO J. 2023 Apr 17;42(10):e111587. doi: 10.15252/embj.2022111587 (PMC10183824; doi:10.15252/embj.2022111587)
Supplement: Supplementary file 5 — PDF+ [file EMBJ-42-e111587-s002.pdf]

Resource

TRANSPARENT PROCESS OPEN ACCESS

THE EMBO JOURNAL

Targeted assembly of ectopic kinetochores to induce chromosome-specific segmental aneuploidies

Laura Tovini[1,†], Sarah C Johnson[2,†], Molly A Guscott[2], Alexander M Andersen[2], Diana Carolina Johanna Spierings[3], René Wardenaar[3], Floris Foijer[3] & Sarah E McClelland[2,*]

Abstract

Cancer cells display persistent underlying chromosomal instability, with individual tumour types intriguingly exhibiting characteristic subsets of whole, and subchromosomal aneuploidies. Few methods to induce specific aneuploidies will exist, hampering investigation of functional consequences of recurrent aneuploidies, as well as the *acute* consequences of specific chromosome mis-segregation. We therefore investigated the possibility of sabotaging the mitotic segregation of specific chromosomes using nuclease-dead CRISPR-Cas9 (dCas9) as a cargo carrier to specific genomic loci. We recruited the kinetochore-nucleating domain of centromere protein CENP-T to assemble ectopic kinetochores either near the centromere of chromosome 9, or the telomere of chromosome 1. Ectopic kinetochore assembly led to increased chromosome instability and partial aneuploidy of the target chromosomes, providing the potential to induce specific chromosome mis-segregation events in a range of cell types. We also provide an analysis of putative endogenous repeats that could support ectopic kinetochore formation. Overall, our findings provide new insights into ectopic kinetochore biology and represent an important step towards investigating the role of specific aneuploidy and chromosome mis-segregation events in diseases associated with aneuploidy.

Keywords aneuploidy; centromere; chromosomal instability; chromosome mis-segregation; kinetochore

Subject Categories Cell Cycle; DNA Replication, Recombination & Repair

DOI 10.15252/embj.2022111587 | Received 3 May 2022 | Revised 31 March 2023 | Accepted 5 April 2023 | Published online 17 April 2023

The EMBO Journal (2023) 42: e111587

Introduction

Whole and partial chromosomal aneuploidies often occur in patterns: human pluripotent stem cells display recurrent aneuploidies (Baker *et al*, 2016) and congenital aneuploidy syndromes affect a small subset of specific chromosomes (Hutaff-Lee *et al*, 2013). Aneuploidy in cancer is also nonrandom, with individual cancer types exhibiting characteristic aneuploidy and somatic copy number alteration (SCNA) landscapes (Taylor *et al*, 2018), and displaying nonrandom chromosome mis-segregation (Klaasen *et al*, 2022). For example, chromosomes 1 and 6 are the most frequently affected by chromosome arm-length aneuploidies in primary retinoblastoma (Kooi *et al*, 2016). In some instances, specific cancer aneuploidies are associated with clinical outcomes, for example the association of monosomy 7 with poor-risk acute myeloid leukaemia (AML) (Breems *et al*, 2008). In general, however, the functional consequences and clinical implications for specific chromosome alterations remain unknown—in large part due to a lack of tractable cell models that allow targeted mis-segregation of specific chromosomes. To date, microcell-mediated chromosome transfer approaches were used to catalogue the impact of specific single chromosome gains and more recently, losses, on cells and have provided important insights into cellular responses to stable expression of a specific aneuploidy (Passerini *et al*, 2016; Chunduri *et al*, 2021; Krivega *et al*, 2021). Such approaches to manipulate karyotypes rely on a period of selection during which acute responses to aneuploidy may be lost, or adapted to. CRISPR-targeted telomere cleavage has been utilised to induce chromosome-specific bridges and track the fate of the bridged chromosome in daughter cells using single-cell microscopy-based isolation methods (Umbreit *et al*, 2020). Similarly, CRISPR-targeted cleavage has been shown to generate damaged chromosomes that form DNA bridges or micronuclei and are subsequently prone to chromothripsis (Leibowitz *et al*, 2021), and cutting and pasting telomere sequences near centromeres using CRISPR-Cas9 can induce specific acentric chromosome formation and loss (preprint: Girish *et al*, 2023). We were thus motivated to discover whether it was possible to develop a platform that would allow a large range of different specific chromosome mis-segregation events to be modelled, without the requirement for chromosomal cleavage.

Elegant prior studies demonstrated that ectopic kinetochores can be induced by artificially recruiting kinetochore-nucleating domains

1 Instituto Gulbenkian de Ciência, Oeiras, Portugal
2 Centre for Cancer Genomics and Computational Biology, Barts Cancer Institute, Queen Mary University of London, London, UK
3 European Research Institute for the Biology of Ageing, University of Groningen, University Medical Center Groningen, Groningen, The Netherlands
*Corresponding author. Tel: +44 (0)20 7882 3840; E-mail: s.mcclelland@qmul.ac.uk
†These authors contributed equally to this work

© 2023 The Authors. Published under the terms of the CC BY 4.0 license.

of the inner kinetochore proteins CENP-T, or CENP-C using LacI fusions in cell lines engineered to harbour a LacO array in noncentromeric chromatin (Gascoigne *et al*, 2011; Hori *et al*, 2013). CENP-C and CENP-T targeting to LacO arrays initiates the recruitment of downstream kinetochore components, demonstrating the potential to bypass the specialised centromeric CENP-A-containing nucleosomes to form an ectopic kinetochore at noncentromeric locations. Moreover, the resulting pseudodicentric chromosomes were subject to faulty chromosome segregation and induced translocations in the LacO-containing chromosome (Gascoigne & Cheeseman, 2013), revealing the potential of this method to induce chromosome mis-segregation. However, several questions remain regarding the functionality of ectopic kinetochores, such as the efficiency of proper kinetochore-microtubule attachment, sister chromatid cohesion or mitotic checkpoint silencing, and how these functions might vary with genomic position. Moreover, the number of protein moieties required to nucleate a functional ectopic kinetochore could not be answered with the previous systems. In addition, the extremely large and repetitive LacO array employed in these studies carries the potential to form a fragile site (Jacome & Fernandez-Capetillo, 2011) and requires genome editing to create stable cell lines harbouring the LacO array. We therefore designed an approach that could be fine-tuned, allowing the provoked mis-segregation of specific chromosomes, in multiple cell types, without the requirement for prior genetic engineering.

Nuclease-dead CRISPR-Cas9 (dCas9) has been extensively used for imaging purposes (Chen *et al*, 2013; Ma *et al*, 2015; Qin *et al*, 2017; Stanyte *et al*, 2018) and also for recruiting functional proteins to the genome, such as transcription factors (Tanenbaum *et al*, 2014; Joung *et al*, 2017) or chromatin remodellers (Saunderson *et al*, 2017), as well as centromere protein CENP-B (Dumont *et al*, 2020). This prompted us to test whether dCas9 could also nucleate the formation of functional kinetochores at ectopic loci in human cells, similar to what has been shown in *S. cerevisiae* (Kuhl *et al*, 2020). This would create a pseudodicentric chromosome that would be more susceptible to mis-segregation due to simultaneous attachment to both centrosomes (Fig 1A). Such a system would allow the use of endogenous repetitive arrays rather than relying on engineered cell lines harbouring LacO arrays, and the position of the ectopic kinetochore could be varied to target different chromosomes, or chromosomal regions for mis-segregation. Here, we demonstrate that dCas9 can efficiently recruit the kinetochore-nucleating domain of CENP-T (amino acids 1–375, hereafter 'CENP-T$^{\Delta C}$') to three endogenous repetitive arrays on chromosomes 1, 3 and 9 in both HEK293T and HCT116 human cell lines. Efficient recruitment of outer kinetochore components was achieved to large (> 1,400 guide RNA bind sites) endogenous repeat loci on chromosomes 1 and 9. Ectopic kinetochores were capable of binding to microtubules; however, attachments were frequently abnormal, culminating in the activation of the mitotic checkpoint and aberrant Mad2 retention on attached kinetochores. Overcoming the mitotic arrest using Mps1 inhibition led to elevated mis-segregation and aneuploidy of the target chromosomes. Live cell imaging revealed that mis-segregating targeted chromosomes rarely entered micronuclei, but frequently formed chromatin bridges. Lastly, we performed an analysis using the telomere-to-telomere human genome reference sequence (Nurk *et al*, 2022), and present an additional putative 23 target repeat sites for ectopic kinetochore formation across 18 of the 23 chromosomes.

Results

dCas9 can recruit CENP-T to ectopic loci at comparable levels to endogenous centromeres

We first created CENP-T$^{\Delta C}$-dCas9 fusion constructs, including a flexible linker between CENP-T$^{\Delta C}$ and dCas9, as previously done when using CENP-T$^{\Delta C}$ to nucleate kinetochores (Gascoigne *et al*, 2011). We also fused 3xEGFP to the C-terminus of dCas9 to allow imaging of targeted loci (Fig 1A). We expressed the CENP-T$^{\Delta C}$-dCas9 fusion protein, using dCas9-EGFP as a control, in HEK293T (human embryonic, transformed) or in HCT116 (human near-diploid colorectal cancer) cells, together with optimised guide RNA scaffolds for increased stability and assembly with dCas9 (Chen *et al*, 2013) (Fig 1C). We first tested ectopic CENP-T recruitment to the *MUC4* gene, a repetitive locus close to the telomere of chromosome 3 previously used to tether dCas9-EGFP for imaging purposes (Chen *et al*, 2013). Specifically, we targeted a region in the second exon of *MUC4*, which contains 100 to 400 repeats of a 48 bp sequence (Nollet *et al*, 1998) with a predicted 44–418 binding sites for sgMUC4 (Fig 1B; Table 1). Targeting CENP-T$^{\Delta C}$-dCas9-EGFP to the *MUC4* locus generated ectopic nuclear foci of CENP-T in the vast majority of transfected (EGFP-positive) HEK293T cells (Fig 1C and D). Quantification of signal intensities in metaphase cells revealed that ectopically recruited CENP-T$^{\Delta C}$ levels were on average approximately 60% of CENP-T levels at endogenous centromeres, with individual ectopic CENP-T$^{\Delta C}$ foci often similar in intensity to lower intensity endogenous centromeres (Fig 1E). However, these ectopic CENP-T$^{\Delta C}$ foci were not sufficient to recruit the downstream kinetochore component KNL-1 during mitosis (Fig 1F–H).

Increasing the dCas9 binding site size initiates recruitment of downstream kinetochore component KNL-1

To test whether recruiting CENP-T$^{\Delta C}$-dCas9 to a larger chromosomal target site would allow the establishment of a functional kinetochore, we directed CENP-T$^{\Delta C}$-dCas9 to two larger endogenous repetitive arrays. These are located at the pericentromere of human chromosome 9 ('Chr9-CEN'; Ma *et al*, 2015) or proximal to telomere of the p-arm of human chromosome 1 ('Chr1-TELO'; Dumont *et al*, 2020), and carry a predicted 556,532–3.8 million and 1,441–7,996 guide RNA-binding sites, respectively (Fig 2A; Table 1). Fluorescence *In-Situ* hybridisation validated dCas9-EGFP targeting to these loci on metaphase spread chromosomes (Fig 2B). Guiding CENP-T$^{\Delta C}$-dCas9 to either of these sites resulted in the formation of ectopic CENP-T$^{\Delta C}$ foci of significantly higher intensity than the *MUC4*-recruited foci in both interphase and mitosis, in both HEK293T and HCT116 cells (Figs 2C–E and EV1A and B) (the presence of three signals for Chr9-CEN is due to the near-triploid karyotype of HEK293T cells; Binz *et al*, 2019). Strikingly, KNL-1 was now efficiently recruited to both Chr9-CEN and Chr1-TELO in both cell lines during prometaphase when CENP-T-mediated KNL-1 loading usually occurs (Hara *et al*, 2018; Figs 2F–H and EV1C and D), resulting in ectopic KNL-1 foci of 3-fold (Chr1-TELO), or 20-fold (Chr9-CEN) higher intensity than endogenous centromeres (Fig 2H). Ndc80 was also efficiently recruited to Chr1-TELO and Chr9-CEN sites (Figs 2I and J and EV1E and F).

© 2023 The Authors

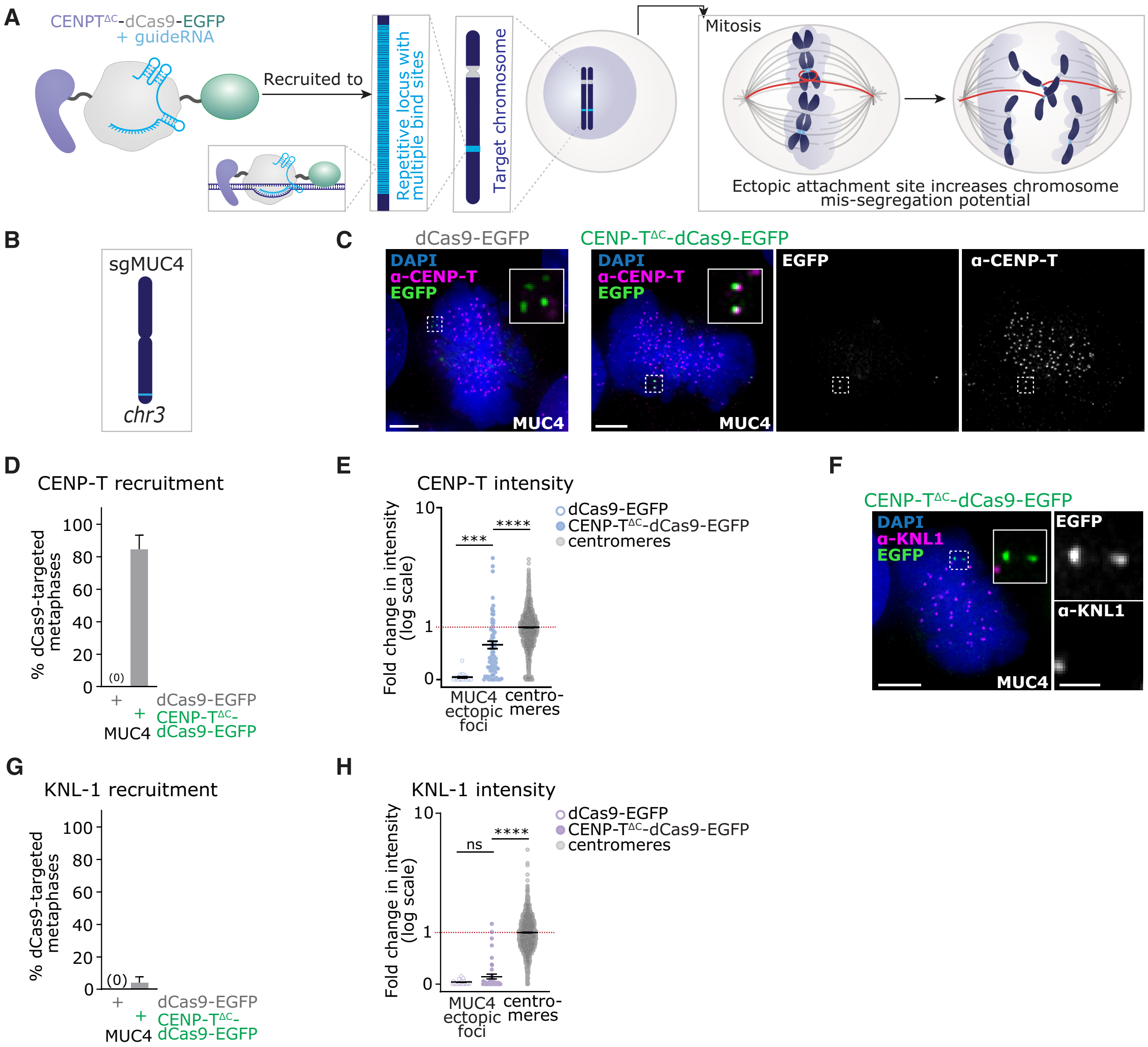

Figure 1. dCas9-mediated recruitment of CENP-T$^{\Delta C}$ foci to an ectopic site on Chromosome 3.

A Strategy for targeted recruitment of an ectopic kinetochore via dCas9. To specifically recruit CENP-T$^{\Delta C}$ to the target chromosome (dark blue), a fusion protein composed of CENP-T$^{\Delta C}$ (lilac), dCas9 (dark grey) and EGFP (green) was used with a guide RNA complementary to a sequence (light blue) within a highly repetitive locus. In mitosis, kinetochore-microtubule (grey/orange) attachment at endogenous centromeres (light grey) and ectopic sites (light blue) renders the target chromosome prone to mis-segregation in anaphase.

B Cartoon of chromosome 3 showing the approximate position of *MUC4* target site (light blue).

C–H Targeting of dCas9-EGFP or CENP-T$^{\Delta C}$-dCas9-EGFP to *MUC4* in HEK293T cells. (C) Immunofluorescence image of dCas9-EGFP or CENP-T$^{\Delta C}$-dCas9-EGFP targeted cells stained with antibodies against CENP-T. (D) Percentage of metaphase cells showing EGFP and CENP-T signal co-localisation. (E) Quantification of CENP-T signal intensity at *MUC4* ectopic foci vs. endogenous centromeres, normalised to CENP-T signal intensity at endogenous centromeres (= 1, red line). (F) Immunofluorescence images of dCas9-EGFP or CENP-T$^{\Delta C}$-dCas9-EGFP targeted cells stained with antibodies against KNL-1. (G) Percentage of metaphase cells showing EGFP and KNL-1 signal co-localisation. (H) Quantification of KNL-1 signal intensity at *MUC4* foci, normalised to KNL-1 signal intensity at centromeres (= 1, red line).

Data information: Data in (D and G) Bars = mean + SD, 3 experiments, each with ≥30 metaphases analysed per condition. Data in (E and H) Lines = mean ± SD, 3 experiments, each with ≥ 10 metaphases analysed per condition. Each point = 1 focus or centromere. $^{ns}P > 0.05$, ***$P < 0.01$, ****$P < 0.001$ (Kruskal Wallis test with Dunn's multiple comparison correction). Images in (C and F) are maximum intensity projections taken across the depth of the EGFP foci. Scale bars = 5 μm on large images, 1 μm on zooms.

© 2023 The Authors

Table 1. Details of guide RNAs used.

sgRNA	Sequence (5′-3′)	Predicted target repeat						
		Chr	Co-ordinates	Distance from (Mb)		Number of binding sites — Allowed mismatches (bp)		
				CEN	TELO	0	1	2
sgMUC4	GGCGTGACCTGTGGATGCTG	3	1,98,560,000–1,98,580,000	102.1	2.5	44	166	418
sgChr9-CEN	TGGAATGGAATGGAATGGAA	9	49,050,000–76,690,000	1.4	49.1	556,532	1,896,253	3,807,221
sgChr1-TELO	GATGCTCACCT	1	2,080,000–2,270,000	119.3	2.1	1,441	4,397	7,996

For each guide, binding sites within the genome were predicted allowing a range of allowed mismatches between site and guide. Co-ordinates are in CHM13_T2T v1.1 reference genome. CEN, centromere; TELO, closest telomere.

Ectopic kinetochores attach to, and stabilise microtubules

Similar to ectopic kinetochores generated with the LacO system (Gascoigne *et al*, 2011), mitotic but not interphase CENP-T$^{\Delta C}$-dCas9 foci recurrently showed a bar-like shape, whereas dCas9-EGFP control foci remained circular during mitosis (Fig 3A). This suggested interaction with mitotic spindle microtubules, and accordingly, these bar-like CENP-T$^{\Delta C}$-dCas9 foci were stretched compared with unattached foci, but reverted to circular morphology upon nocodazole-induced microtubule depolymerisation (Fig 3B and C). We therefore examined CENP-T$^{\Delta C}$-dCas9 foci for the presence of stably attached spindle microtubules as an indication of the functionality of the ectopic kinetochore. Cells were cold-treated for 10 min to depolymerise nonkinetochore attached microtubules (Brinkley & Cartwright Jr., 1975). The majority of mitotic cells exhibiting ectopic kinetochores at either chromosome 1, or chromosome 9, displayed obvious kinetochore fibres terminating at the ectopic CENP-T$^{\Delta C}$/KNL-1 site which were often abnormally large at Chr9-CEN (Fig 3D and E). Taken together, these data suggest that dCas9-tethered CENP-T$^{\Delta C}$ is able to recruit functionally active downstream kinetochore components and provide attachment to mitotic spindle microtubules.

CENP-T$^{\Delta C}$-nucleated kinetochores trigger the mitotic spindle assembly checkpoint

The expected outcome of an ectopic kinetochore is the creation of a "pseudo-dicentric" chromosome, which has an increased chance of becoming incorrectly attached to microtubules and is therefore more prone to mis-segregation (Gascoigne & Cheeseman, 2013; see schematic in Fig 1A). To test whether this would occur with dCas9-nucleated ectopic kinetochores, we performed live cell imaging of HEK293T cells with dCas9-CENP-T$^{\Delta C}$ targeted to Chr9-CEN (Fig 3F; Movie EV1). Strikingly, most mitotic cells exhibited a prolonged mitotic delay; the average length of metaphase in cells with dCas9-CENP-T$^{\Delta C}$ ectopic kinetochores was 175 min compared with 74 min in dCas9-EGFP-targeted cells and 50 min in EGFP-negative cells (Fig 3G and H). Since cells with ectopic kinetochores were often already in metaphase at the start of the movie, this was also an underestimation of the metaphase delay.

Ectopic kinetochores form a range of attachment types including a Mad2-positive attached state

To examine the reasons for the mitotic arrest in more detail, we co-stained cold-treated cells with Mad2 and tubulin antibodies and characterised the attachment status of ectopic kinetochore sites. Whilst amphitelic attachments were observed at some ectopic kinetochores (~ 25%), the majority displayed erroneous attachments predominantly in the form of merotely and monotely (Fig 4A and B). Meanwhile, Mad2 staining revealed the majority of individual CENP-T$^{\Delta C}$-dCas9 kinetochores to be Mad2-bound in metaphase (Fig 4C and D). In line with normal Mad2 function (Waters *et al*, 1998), all unattached ectopic kinetochores were Mad2-positive (Fig 4E and F). However, we also observed an unusual state: approximately 60% of single attached ectopic kinetochores retained clear Mad2 loading (Fig 4G and H). This observation reconciled our previous observations that 80% cells with ectopic kinetochores had stabilised tubulin bundles terminating at ectopic kinetochores, yet 60% cells arrested in metaphase for longer than 120 min (Fig 3E and H). We conclude that although ectopic kinetochores are capable of attaching to microtubules, a high proportion of attachments do not correctly regulate the removal of Mad2 (see discussion for possible reasons).

Aurora B activity contributes to a CENP-T$^{\Delta C}$-nucleated ectopic kinetochore-induced mitotic arrest

The observed metaphase arrest and retention of Mad2 on ectopic kinetochores suggested an activated mitotic checkpoint. Accordingly, treating CENP-T$^{\Delta C}$-dCas9-targeted cells with Mps1 kinase inhibitor NMS-P715 (Colombo *et al*, 2010; hereafter "Mps1i") to abrogate the mitotic checkpoint (Tighe *et al*, 2008) allowed the majority of metaphase-arrested cells to progress into anaphase while not significantly affecting metaphase: anaphase ratios in dCas9-EGFP control-targeted cells (Figs 5A and B and EV2A and B). Given our observations of merotelic, syntelic and unattached ectopic kinetochores (Fig 4B), we reasoned that Aurora B-mediated detachment of improperly attached ectopic kinetochores could contribute to the activation of the mitotic checkpoint. To test this, we inhibited Aurora B in transfected cells using ZM447439 (Ditchfield *et al*, 2003). Aurora B inhibition reduced the metaphase arrest seen upon targeting CENP-T$^{\Delta C}$-dCas9 to either Chr9-CEN or Chr1-TELO, matching the efficiency of Mps1 inhibition in HCT116 cells with Chr9 ectopic kinetochores (Figs 5A and B, and EV2A, B, and E). In some cells, however, inhibition of Aurora B was insufficient to overcome the metaphase arrest (particularly in HEK293T cells; Figs 5A and B).

CENP-T$^{\Delta C}$-nucleated ectopic kinetochores induce specific mis-segregation of targeted chromosomes

We reasoned that abrogating the mitotic arrest by inhibiting the Mps1 kinase (Tighe *et al*, 2008) would allow us to score the impact

 © 2023 The Authors

of ectopic kinetochore assembly on chromosome segregation fidelity. We therefore scored CENP-T$^{\Delta C}$-dCas9-targeted chromosome segregation error rates from anaphase cells that were visible after a short Mps1i pulse, exploiting the EGFP fluorescence present on ectopic kinetochores to detect the target chromosome in the act of mis-segregation. We classified cells as exhibiting either (i) EGFP-

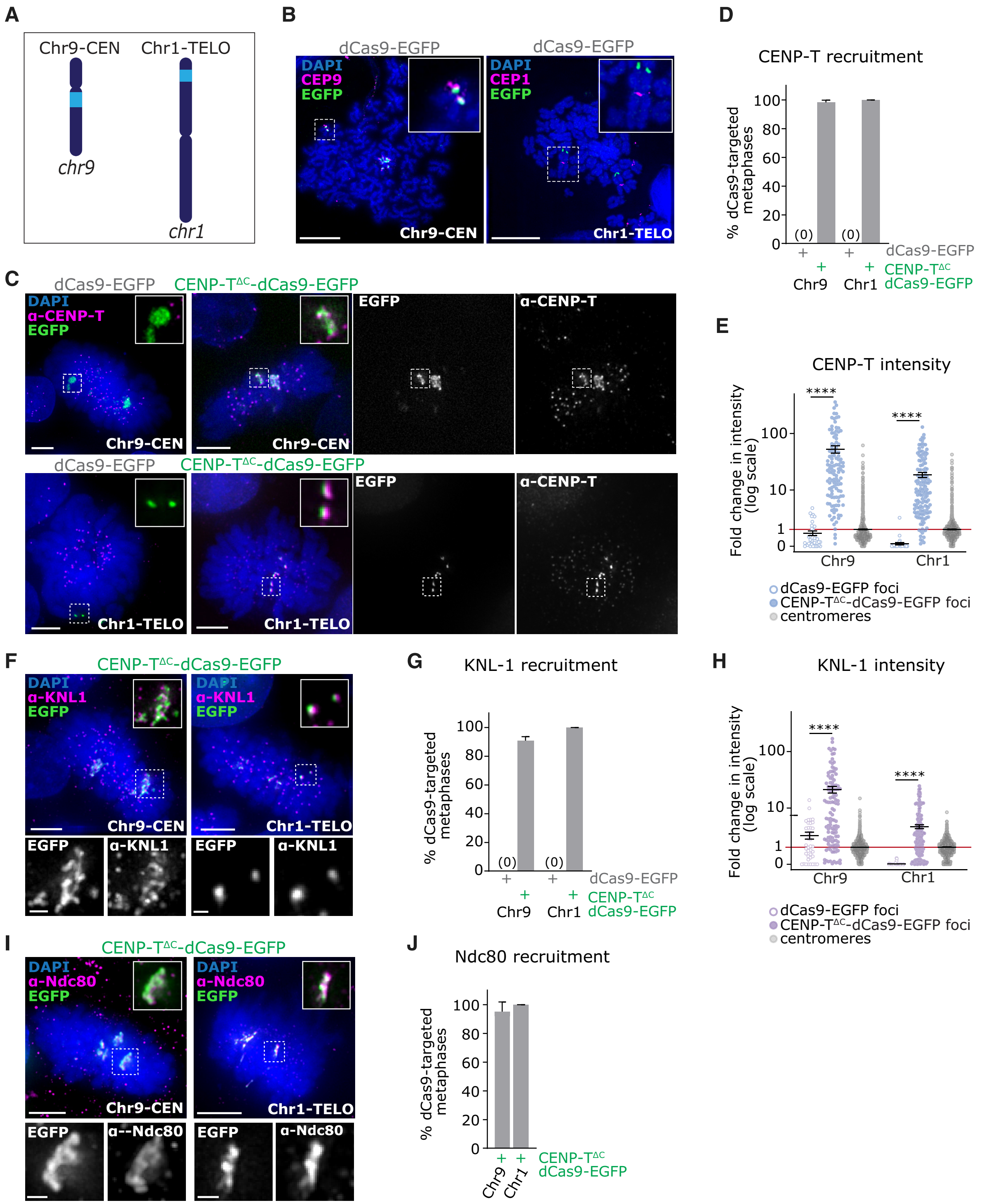

Figure 2.

Figure 2. CENP-T$^{\Delta C}$-dCas9 targeting recruits KNL-1 and Ndc80 to large repetitive chromosomal loci.

A Cartoon showing the approximate position of the Chr9-CEN and Chr1-TELO target sites (light blue).

B–J Targeting of dCas9-EGFP or CENP-T$^{\Delta C}$-dCas9-EGFP to Chr9-CEN and Chr1-TELO in HEK293T cells. (B) FISH images on chromosome spreads from cells with dCas9-EGFP targeting. (C) Immunofluorescence images of dCas9-EGFP or CENP-T$^{\Delta C}$-dCas9-EGFP targeted cells stained with antibodies against CENP-T. (D) Percentage of metaphase cells showing EGFP and CENP-T signal co-localisation. (E) Quantification of CENP-T signal intensity at Chr9-CEN and Chr1-TELO ectopic foci vs. endogenous centromeres, normalised to CENP-T signal intensity at endogenous centromeres (= 1, red line). (F) Immunofluorescence images of CENP-T$^{\Delta C}$-dCas9-EGFP targeted cells stained with antibodies against KNL-1. (G) Percentage of metaphase cells showing EGFP and KNL-1 signal co-localisation. (H) Quantification of KNL-1 signal intensity at Chr9-CEN and Chr1-TELO foci vs. endogenous centromeres, normalised to KNL-1 signal intensity at centromeres (= 1, red line). (I) Immunofluorescence images of dCas9-EGFP or CENP-T$^{\Delta C}$-dCas9-EGFP targeted cells stained with antibodies against Ndc80. (J) Percentage of metaphase cells showing EGFP and Ndc80 signal co-localisation.

Data information: Data in (D, G and J) Bars = mean + SD, 3 experiments, each with ≥50 metaphases per condition. Data in (E and H) Lines = mean ± SD, 3 experiments, each with ≥ 10 metaphases per condition. ****$P < 0.001$ (Kruskal–Wallis test with Dunn's multiple comparison correction). Images in (B, C, F and I) are maximum intensity projections taken across the depth of the EGFP foci. Scale bars = 5 µm on large images, 1 µm on zooms.

negative only (ii) EGFP-positive only or (iii) EGFP-positive and EGFP-negative errors. Cells with ectopic kinetochores displayed significantly higher EGFP-positive chromosome mis-segregation rates than dCas9-EGFP controls (Fig 5E and F). This was true for both chromosome 9 and 1, and in both HEK293T and HCT116 cells (Figs 5C–F and EV2C and D). Note that given the resolution of the microscopy, we cannot determine whether mis-segregating chromatin was composed of whole intact chromosomes, or partial and/or damaged chromosomes. Since the use of Mps1i induces some EGFP-negative errors, we also tested a lower concentration of Mps1i in an attempt to reduce background error rates. Although this proved ineffective in HEK293T cells with the lower doses yielding no reduction in segregation error rate, slightly decreased segregation error rates were observed in HCT116 cells (Fig EV2F–G). This lower concentration was, however, markedly less able to relieve CENP-T$^{\Delta C}$-dCas9-induced mitotic arrest in HEK293T cells (Fig EV2H). We therefore conclude that it may be possible to reduce background segregation error rates in some cell lines, but this may come at the cost of reducing the efficiency of mitotic arrest bypass.

To track the fate of mis-segregating chromatin, we performed a set of live cell experiments combined with an Mps1i pulse. Metaphase cells with dCas9-EGFP foci were identified under the microscope then Mps1i was applied, points refocussed, and cells imaged for the following 4 h (Fig 5G). In accordance with the fixed cell data (Fig 5A), metaphase cells with CENP-T$^{\Delta C}$-dCas9 foci released into anaphase approximately 30 min following the addition of Mps1i, with over 80% of cells displaying mis-segregating EGFP-positive chromatin (Fig EV3A–D). Most cells exhibiting EGFP-positive lagging chromatin formed daughter cells with unbalanced EGFP signals indicating mis-partitioning of the target chromosome (Fig 5H–I). In addition, a large proportion of ectopic kinetochore mis-segregating cells exhibited unresolved EGFP-positive bridges of which ~ 75% failed to resolve for the remainder of the experiment (~ 3 h) (Figs 5G–I and EV3E; Movie EV2). We also wondered whether mis-segregated EGFP-positive chromatin might become incorporated into micronuclei; however, this was observed only rarely, in fewer than 5% of cells (Figs 5I and EV3C; Movie EV2).

Induction of segmental aneuploidies on target chromosomes

To directly test the consequences of targeted mis-segregation on aneuploidy of daughter cells, we sequenced individual G1 cells following transfection and a pulse of Mps1i. Cells were allowed to complete mitosis and separate into two daughter cells before undergoing single-cell low-pass whole-genome sequencing. HEK293T cells contain a rearranged, but stable karyotype with only minor translocations involving chromosome 1 or 9 (Binz *et al*, 2019). We therefore computed copy number alterations (CNAs) compared with a pseudo bulk HEK293T reference obtained from our control (Mps1i, no guide no dCas9) population of sequenced cells (Figs 6A and EV4A; See Materials and Methods). ClonalMasker (Shaikh *et al*, 2022) (see Materials and Methods) was used to remove clonal and subclonal aneuploidies (present in identical positions across more than four cells) present in the relevant nontargeted controls from each additional condition, and report back only unique aneuploidies (see pileups in Fig EV4B and C). We then scored partial and whole chromosome aneuploidy events > 20 Mb for each chromosome, across all conditions (Figs 6B–D and EV4D). Determining new aneuploidy events occurring on a heterogeneous background is challenging, and in addition only approximately 40% cells displayed CENP-T$^{\Delta C}$-dCas9 targeting within this experiment. Nonetheless, with CENP-T$^{\Delta C}$ targeting to chromosome 1 and 9, we were able to detect a statistically significant enrichment in partial aneuploidies affecting the target chromosomes (Fig 6D). Interestingly, the dCas9-EGFP control targeting to chromosome 1 also induced segmental aneuploidies of chromosome 1 although we interpret this result with caution since chromosome 1 exhibited a high frequency of alteration across most conditions likely due to additional subclonal aneuploidies present in the parental line that failed the threshold for removal by ClonalMasker (Figs 6C and EV4B). Closer examination of induced CNAs (Fig 6B and C) revealed differences in the distribution of breakpoints observed when targeting Chr9 vs Chr1, potentially reflecting the pericentromeric vs telomere-proximal positions of the loci. For CENP-T$^{\Delta C}$ targeting to Chr9-CEN (Fig 6B), induced CNAs were all found with breakpoints in the q-arm proximal to the pericentromeric target site. Meanwhile, ectopic kinetochore targeting to Chr1-TELO (Fig 6C) led to an enrichment of breakpoints between the target telomere and endogenous centromere but at a larger distance from the actual target site, suggesting physical breakage of the chromosome between two opposing attachment sites as expected for a dicentric chromosome. Although two of the large CNAs originated in the Chr9 guide locus we did not observe any focal CNAs (<20 Mb) within the target site itself (Fig 6E and F). Taken together, these data show we can use CENP-T$^{\Delta C}$-dCas9 to induce specific chromosome mis-segregation and subchromosomal aneuploidy.

 © 2023 The Authors

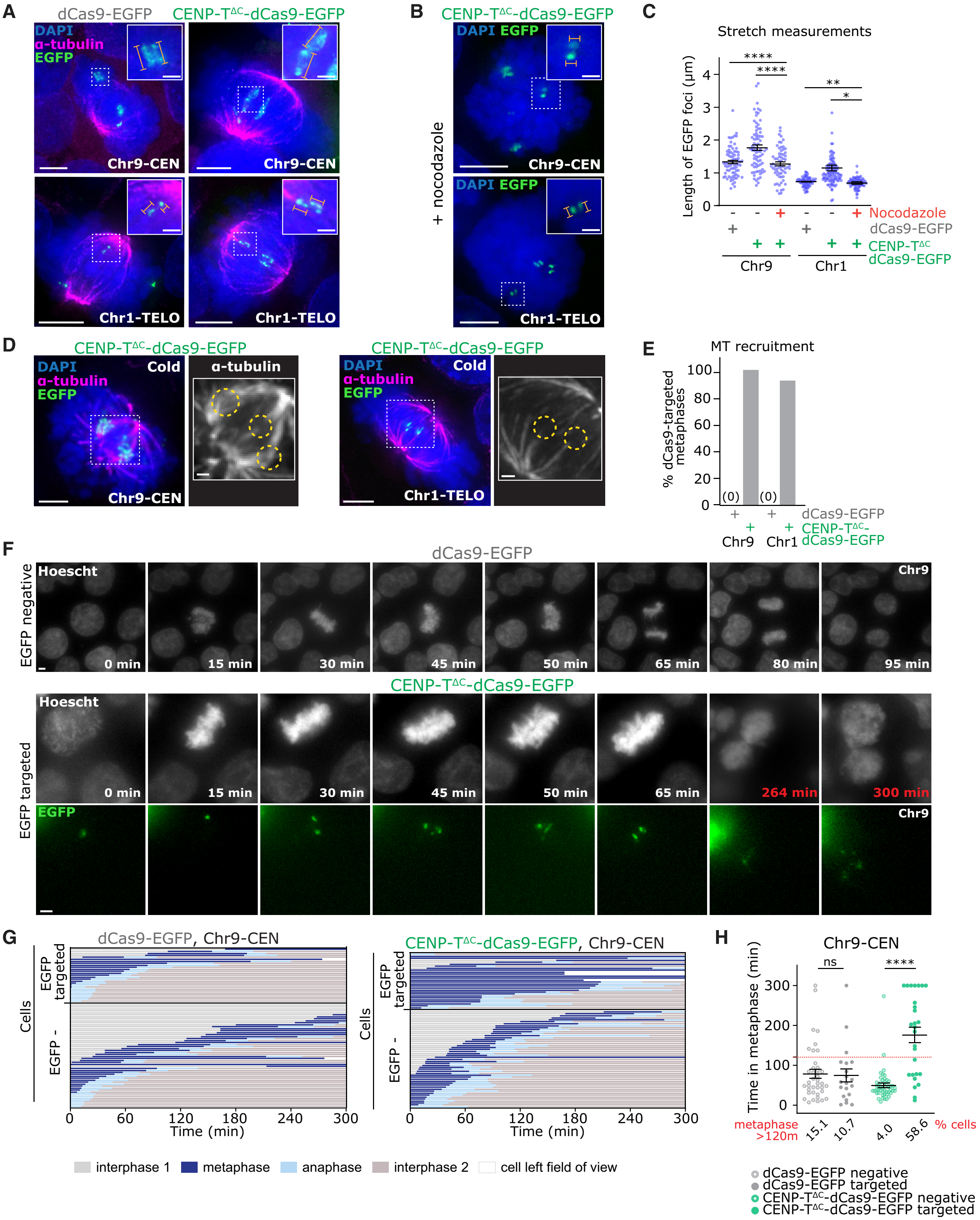

Figure 3.

© 2023 The Authors

Figure 3. CENP-T$^{\Delta C}$-dCas9-nucleated ectopic kinetochores recruit microtubules but induce mitotic delay.

Targeting of dCas9-EGFP or CENP-T$^{\Delta C}$-dCas9-EGFP to Chr9-CEN and Chr1-TELO in HEK293T cells.

A Immunofluorescence images of dCas9-EGFP or CENP-T$^{\Delta C}$-dCas9-EGFP targeted metaphase cells stained with antibodies against alpha-tubulin.

B Immunofluorescence images of mitotic cells with CENP-T$^{\Delta C}$-dCas9-EGFP targeting after nocodazole treatment.

C Length measurements of individual EGFP foci in μm. Example measurements are indicated on zooms in (A, B) in orange. Data is ≥ 20 cells per condition, from 1 experiment. Lines = mean ± SEM. *$P < 0.05$, **$P < 0.01$, ****$P < 0.001$ (Kruskal-Wallis with multiple comparison correction).

D Immunofluorescence images of metaphase cells with CENP-T$^{\Delta C}$-dCas9-EGFP targeting after cold treatment, stained with antibodies against alpha-tubulin.

E Percentage of metaphase cells showing microtubule (MT) recruitment. ≥50 metaphases per condition, from one experiment..

F Frames from live cell imaging of cells with dCas9 targeted to Chr9-CEN, or an EGFP-negative cell.

G Cell cycle stages of cells with CENP-T$^{\Delta C}$-dCas9-EGFP or dCas9-EGFP transfected cells, by live-cell imaging (each line = 1 cell, ≥ 27 cells per group), showing EGFP negative and EGFP targeted cells separately.

H Time spent in metaphase for CENP-T$^{\Delta C}$-dCas9-EGFP or dCas9-EGFP transfected cells, showing EGFP negative and EGFP targeted cells separately. Dotted line = 120 min = cut-off for the longest time that most control cells spent in metaphase. Lines = mean ± SEM. $^{ns}P > 0.05$, ****$P < 0.001$ (One-way ANOVA with Šidák's multiple comparison correction).

Data information: Data in (G and H) are ≥ 20 cells per condition, combined from ≥1 experiment. Images in (A, B, D, F) are maximum intensity projections taken across the depth of the EGFP foci. Scale bars = 5 μm on large images, 1 μm on zooms. In (A, B) Orange bars = measured lengths in (C).

Additional putative guide target sites

Having successfully targeted endogenous repeats in human chromosomes 1 and 9, we were interested to expand the repertoire of targetable chromosomes and so set out to identify putative repeat sequences in the human genome for ectopic kinetochore seeding. Such analyses have previously been hindered by limited resolution of repetitive regions due to alignment and mapping difficulties; however, the newly released T2T consortium long-read sequencing-based reference genome (Nurk *et al*, 2022) allowed us to interrogate the genome for additional repetitive target sites (Fig 7A). Having determined that 1,400 bind sites were amply sufficient to nucleate an ectopic KT, whilst 44 were not, we chose to use a lower threshold of 500 guide bind sites to provide a wide range of target region sizes for future refinement of the number of dCas9-CENP-T molecules required to form a functional ectopic kinetochore. As we have not characterised the consequences of CENP-T$^{\Delta C}$ targeting to endogenous centromeres, we separated centromere-external and internal repeats. To ensure specificity of targeting, any guides that had a cluster of >40 binding sites in a nontarget chromosome were excluded. External to centromeres, we identified 23 putative target repeats distributed over 18 chromosomes with sgRNAs predicted to bind over 500 times (range 501–30,008 binding sites) (Figs 7B and C and EV5A). These repeats were all confined to pericentromeric and telomere-proximal regions of the chromosomes. We also identified 19 of 23 centromeres to be targetable with sgRNAs binding more than 500 times (Fig EV5B and C). Together, this provides a large resource (Dataset EV1) to target ectopic kinetochore formation to different chromosomes, and also to further refine the number of dCas9-CENP-T proteins required to nucleate a functioning ectopic kinetochore.

Discussion

In this study, we induced the mis-segregation of specific human chromosomes using dCas9-CENP-T$^{\Delta C}$ to nucleate ectopic kinetochores at endogenous repeat arrays. Large endogenous repetitive arrays in chromosomes 1 and 9 allowed the efficient recruitment of ectopic kinetochores that bound microtubules and caused elevated mis-segregation and aneuploidy rates of those chromosomes. In an accompanying manuscript (Truong *et al*, 2023; this issue of EMBO J), tethering of a plant kinesin using TetR repeats, or dCas9 similarly induced elevated mis-segregation of targeted chromosomes. Additionally, a recent study revealed the ability of dCas9-based recruitment of mutant KNL1 to centromeres to induce chromosome specific mis-segregation (Bosco *et al*, 2023). Together, these studies demonstrate the flexibility with which dCas9 fusion proteins can be used to interfere with chromosome segregation and provide an important step towards custom manipulation of mitosis to induce chromosome-specific mis-segregation and segmental aneuploidies.

Length and position of the target array are important factors for the formation of dCas9-based ectopic kinetochores

The smallest array (the *MUC4* locus on chromosome 3), despite recruiting CENP-T$^{\Delta C}$ to comparable levels at lower intensity endogenous centromeres, was unable to assemble an ectopic kinetochore. Instead, arrays providing upwards of 1,441 guide RNA-binding sites were able to form functional kinetochores, providing the boundaries for the length of target arrays required to assemble CENP-T-nucleated ectopic kinetochores, though we did not determine the precise threshold in this study. Reasons why near-endogenous levels of ectopic CENP-T$^{\Delta C}$ were insufficient to recruit KNL-1 are not clear but could include the lack of an endogenous CENP-A-CENP-C nucleation pathway (Hori *et al*, 2008; Rago *et al*, 2015) to support the CENP-T$^{\Delta C}$ kinetochore nucleation. This would imply a potential synergy between these two pathways that could have been obscured by the very high binding levels of ectopic CENP-T and CENP-C in previous studies. Nonetheless, it is surprising that KNL-1 was essentially undetectable at these MUC4-targeted CENP-T$^{\Delta C}$, potentially suggesting non-linear loading of KNL-1 to ectopic CENP-T$^{\Delta C}$. Proper phospho-regulation (Hara *et al*, 2018) of the ectopic CENP-T$^{\Delta C}$ may also be dysregulated, which could impair efficient KNL-1 loading (until the point at which this can be overcome by overloading CENP-T$^{\Delta C}$). Further investigation into the phosphorylation state of proteins at ectopic kinetochores would be very interesting and may also shed light on the incorrect regulation of Mad2 removal (see below).

In terms of positioning, we were able to provoke mis-segregation using both centromere-, and telomere-proximal sites for ectopic kinetochore formation. Initially, we had hypothesised that the longer the distance between the native and ectopic kinetochores, the greater the likelihood of that chromosome being mis-

© 2023 The Authors

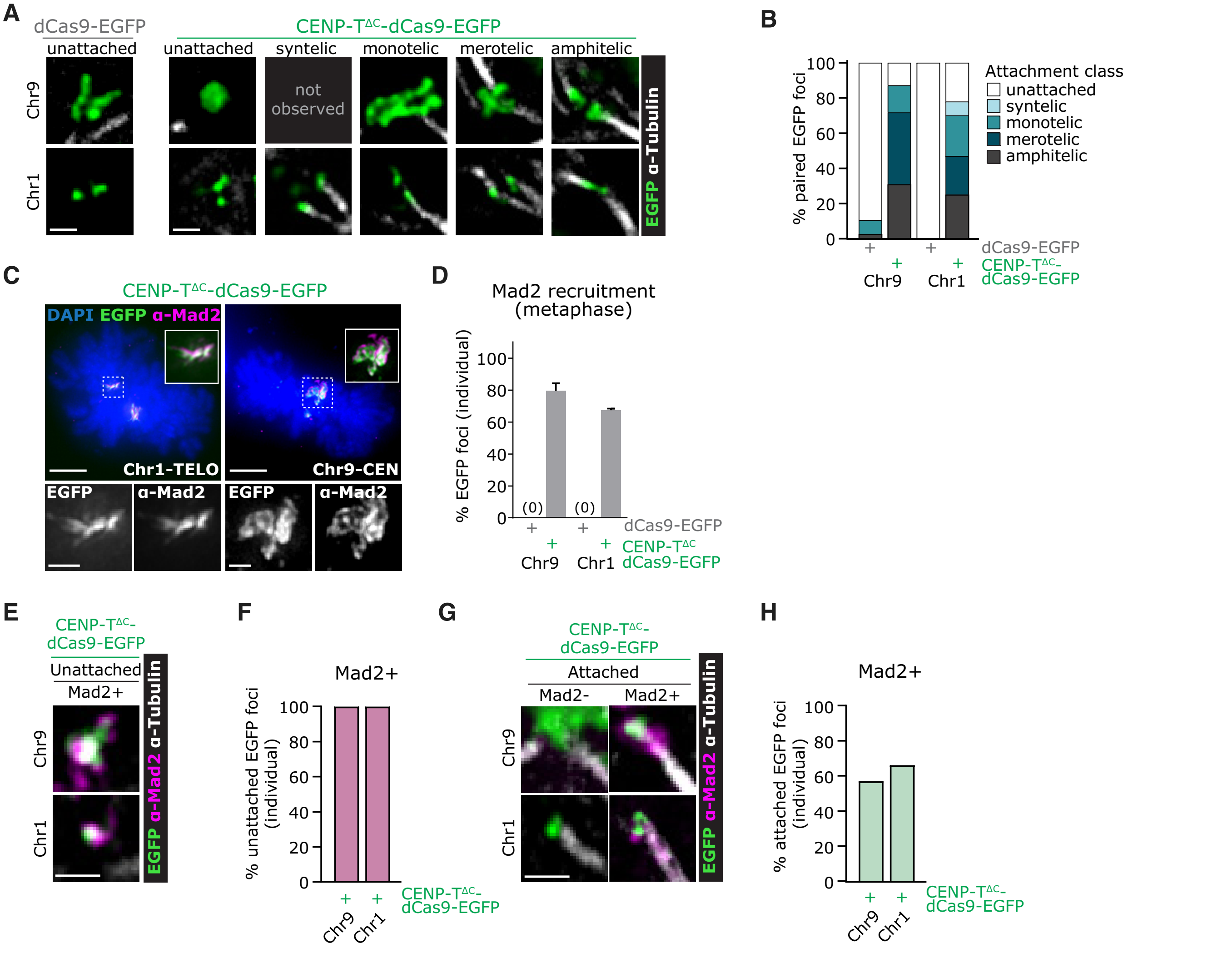

Figure 4. CENP-T$^{\Delta C}$-dCas9-nucleated ectopic kinetochores display a range of attachment types including an unusual Mad2-positive attached state.

Targeting of dCas9-EGFP or CENP-T$^{\Delta C}$-dCas9-EGFP to Chr9-CEN and Chr1-TELO in HEK293T cells.

A Immunofluorescence images of paired EGFP foci for each attachment status category, taken from cold-treated metaphase cells. Images were chosen where both the EGFP foci and any of their attachments lay in the same Z-plane for clarity of presentation.

B Percentage of paired EGFP foci showing each attachment status.

C Immunofluorescence images of CENP-T$^{\Delta C}$-dCas9-EGFP targeted cold-treated metaphase cells stained with antibodies against Mad2.

D Percentage of individual EGFP foci showing EGFP and Mad2 signal co-localisation, in metaphase cells with CENP-T$^{\Delta C}$-dCas9-targeting.

E–H (E, G) Immunofluorescence images from CENP-T$^{\Delta C}$-dCas9-EGFP targeted cold-metaphase cells stained with antibodies against Mad2 and alpha-Tubulin, showing examples of microtubule unattached (E) or attached (G) EGFP foci. (F, H) Percentage of individual EGFP foci with EGFP and Mad2 signal co-localisation, for microtubule unattached (F) or attached (H) EGFP foci.

Data information: Data in (B, D, F, H) are from ≥75 EGFP foci pairs from $n \geq 15$ metaphases per condition, 1 experiment for (B, F, H) and 2 experiments for (D). In (D) Bars = mean ± SD. Images in (A, E, G) are single Z-slices. Image in (C) is a maximum intensity projection taken across the depth of the EGFP signal. Scale bars = 1 µm on small images and zooms, 5 µm on larger images in (C).

segregated, due to a higher probability that a twist in the sister chromatid would allow attachment of the same sister chromatid to both centrosomes (Fig 1A). However, we discovered that in fact, these ectopic kinetochores were intrinsically prone to improper attachment regardless of their position relative to the endogenous centromere, meaning that this approach can be used to mis-segregate specific chromosomes independently of the positioning of the ectopic kinetochore.

CENP-T nucleated kinetochores are largely attached, but activate the mitotic checkpoint

Despite forming attachments to microtubules, and aligning at the metaphase plate, CENP-T$^{\Delta C}$-nucleated kinetochores could not readily satisfy the mitotic checkpoint. In addition to canonical mitotic checkpoint signalling from unattached ectopic kinetochores, it is likely that ongoing error correction of erroneous attachments also

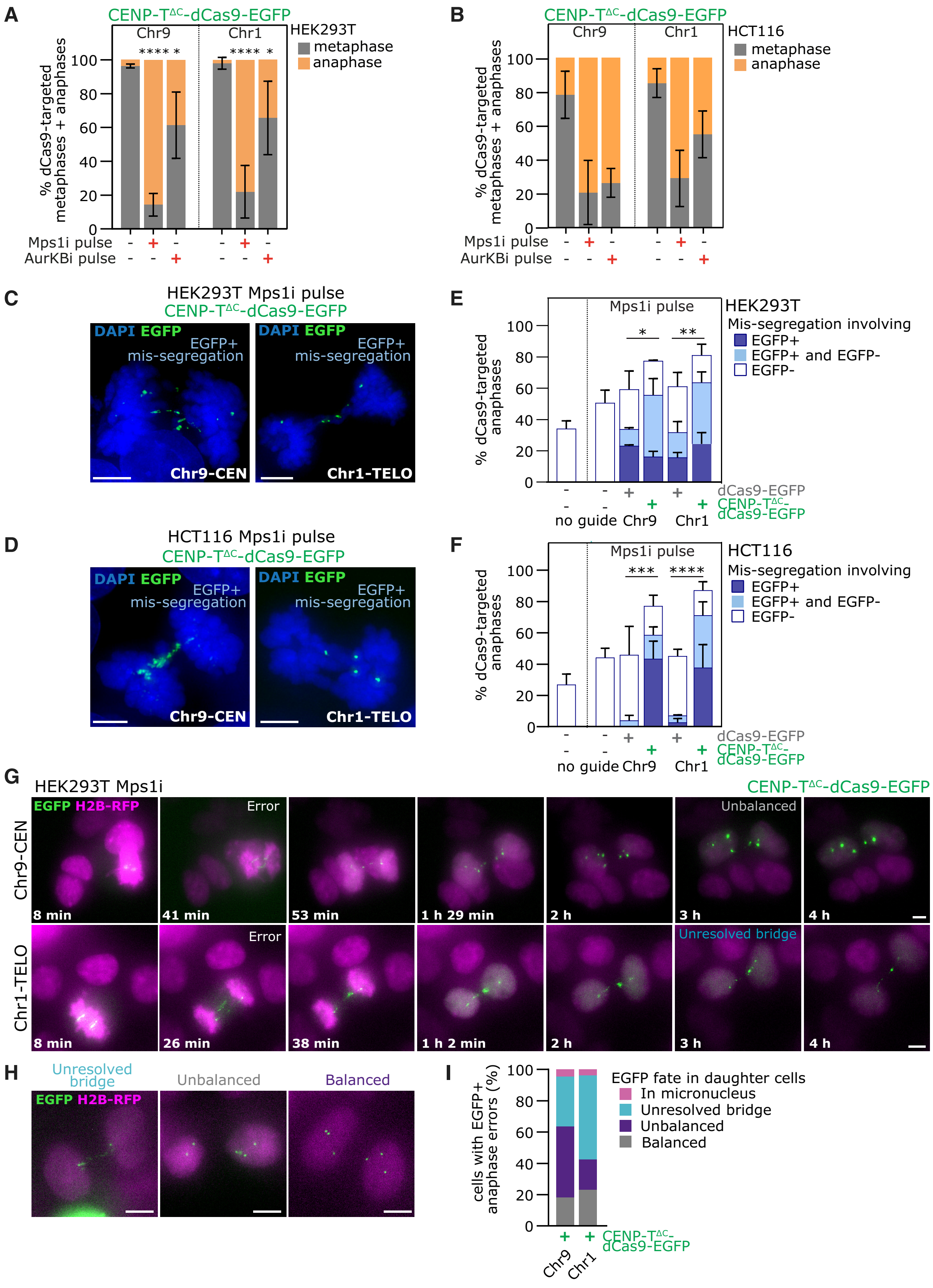

Figure 5.

© 2023 The Authors

Figure 5. Induction of specific chromosome mis-segregation by ectopic kinetochore strategy.

A, B Quantification of mitotic stage from fixed CENP-T$^{\Delta C}$-dCas9 targeted HEK293T (A) or HCT116 (B) cells following inhibition of Mps1 (Mps1i) or Aurora Kinase B (AurKBi). Bars = mean ± SD, 3 experiments for HEK293T, 2 for HCT116, each with ≥ 50 mitotic cells per condition.

C, D Example images of chromosome mis-segregation events involving the target locus (EGFP signal) in anaphase HEK293T (C) or HCT116 (D) cells with CENP-T$^{\Delta C}$-dCas9-EGFP targeting after Mps1i pulse.

E, F Mis-segregation rate in fixed dCas9-targeted HEK293T (E) or HCT116 (F) cells after Mps1i pulse, involving only EGFP+ chromosomes, only EGFP- chromosomes or both. Bars = mean ± SD, 3 experiments for each cell line, each with ≥25 anaphase cells per condition.

G, H Frames from live cell imaging of HEK293T H2B-RFP cells after treatment with Mps1i (t = 0), with CENP-T$^{\Delta C}$-dCas9-EGFP targeted to Chr9-CEN or Chr1-TELO. Frames are either across the duration of the 4 h movie (G) or show examples of the different categories of fate (H).

I Percentage of CENP-T$^{\Delta C}$-dCas9-EGFP targeted cells that made an EGFP+ error in anaphase, categorised by subsequent fate of EGFP signal in daughter cells 3 h after Mps1i addition. Assessed by live cell imaging. ≥ 22 cells with error per condition, combining data from ≥ 3 independent experiments.

Data information: Statistics in (A, E, and F) *$P < 0.05$, **$P < 0.01$, ***$P < 0.001$, ****$P < 0.0001$ (Oneway ANOVA with Šidák's multiple comparison correction, in (E/F) comparing the mis-segregation rate for any events involved EGFP). (C, D, G and H) Scale bars = 5 μm.

contributes to mitotic checkpoint activation; Aurora B inhibition partly overcame the mitotic arrest and approximately 30–40% of ectopic kinetochores displayed attachment states (e.g. merotelic and syntelic, Fig 4B) normally subject to Aurora B-mediated detachment and subsequent checkpoint activation by the resulting unattached kinetochore. Although Aurora B's role in establishment of the mitotic checkpoint *per se* (Hauf *et al*, 2003; Santaguida *et al*, 2011; Gurden *et al*, 2018) means this result should be interpreted with caution, the frequency of unattached ectopic kinetochores observed is not sufficient to explain the arrest seen without additional mitotic checkpoint activation from other sources.

In addition, Mad2-positivity status interestingly revealed that ~60% of attached kinetochores retained Mad2. This is a very unusual kinetochore state, though has been observed previously in conditions where Aurora Kinase B is relocalised away from the inner centromere to the kinetochore (Hengeveld *et al*, 2017; Hayward *et al*, 2022). We favour the possibility that the aberrant Mad2 retention at our ectopic kinetochore stems from an imbalance of kinetochore protein stoichiometry, or loss of normal spatial relationships. For instance, malfunction of Dynein-mediated stripping of Mad2, overloading of KNL1 as the catalytic platform for the mitotic checkpoint complex formation, or imbalances in phosphatase/kinase activity could all contribute to the phenotype that we observe. The abnormal Mad2 state observed at dCas9-nucleated ectopic kinetochores could therefore provide a model system to investigate requirements for proper coupling of Mad2 status to attachment status, and as such provides an ideal platform to study mitotic checkpoint regulation in future studies.

Sequencing reveals a range of subchromosomal alterations induced by ectopic kinetochore formation

To characterise the impact of targeted chromosome mis-segregation, we sequenced genomes of single daughter cells after a single round of mitosis. We observed mainly subchromosomal aneuploidies, suggesting that either only partial chromosomes are observed mis-segregating (Fig 5) or that lagging chromosomes are subject to damage during cell division that results in partial chromosome copy number changes in daughter cells. Indeed, our observations of EGFP chromatin bridges by live cell imaging would be consistent with the latter. Interestingly, however, we did not frequently observe breakpoints (copy number change points) within the guide locus, mainly noting them instead in regions between the target locus and the centromere. This suggests either the breaks indeed occur externally to the targeted region, or that we are unable to detect breaks within this region for technical reasons. These could include (i) low resolution of CNA detection (approximately <0.5 Mb lower bound; Shaikh *et al*, 2022) (ii) low number of cells sequenced, and events observed in general or (iii) the repetitive nature of guide locus hindering detection of focal CNAs.

Benefits and limitations of our approach compared with prior systems

Here, we set out to mimic specific chromosome mis-segregation events and the resulting aneuploidies. We were able to efficiently assemble ectopic kinetochores and induce specific chromosome mis-segregation in two cell lines. Compared with CRISPR-cutting-based approaches, our ectopic kinetochore-mediated mis-segregation rate was high (61–70% transfected cells were induced to mis-segregate the target chromosome (Fig 5C–F), compared to 7.5% for MN generation by CRISPR cutting; Leibowitz *et al*, 2021). Second, our approach could be considered an accurate model of cancer chromosome mis-segregation, by mimicking the formation of dicentric chromosomes (Gisselsson *et al*, 2000; Mackinnon & Campbell, 2011), and faulty kinetochore-microtubule attachments (Cimini *et al*, 2001; Thompson & Compton, 2008). Furthermore, our method provides a tuneable system to further investigate kinetochore assembly regulation, to complement previous studies (Gascoigne *et al*, 2011; Hori *et al*, 2013; Kuhl *et al*, 2020), for example allowing investigation of the impact of assembling ectopic kinetochores at differing locations or chromosomal contexts.

We also note some limitations of our approach. The major outcome of the induced segregation errors was segmental aneuploidies, rather than whole chromosome aneuploidies, potentially adding complexity to the study of downstream consequences of induced specific chromosome instability events. Additionally, a brief pulse of Mps1 inhibition was required to overcome the mitotic arrest. Since Mps1 inhibition itself can induce chromosome segregation errors, this treatment elevated background mis-segregation. We note, however, that this treatment caused only a moderate increase above the basal error rate in both the cell lines tested. Although only a low rate of micronucleus formation was observed, the majority of mis-segregating target chromosomes led to chromatin bridges that persisted for several hours. Further study is therefore required to determine their ultimate fate; for example, previous work has shown that chromatin material from anaphase bridges can form micronuclei in the second cell division (Umbreit *et al*, 2020).

© 2023 The Authors

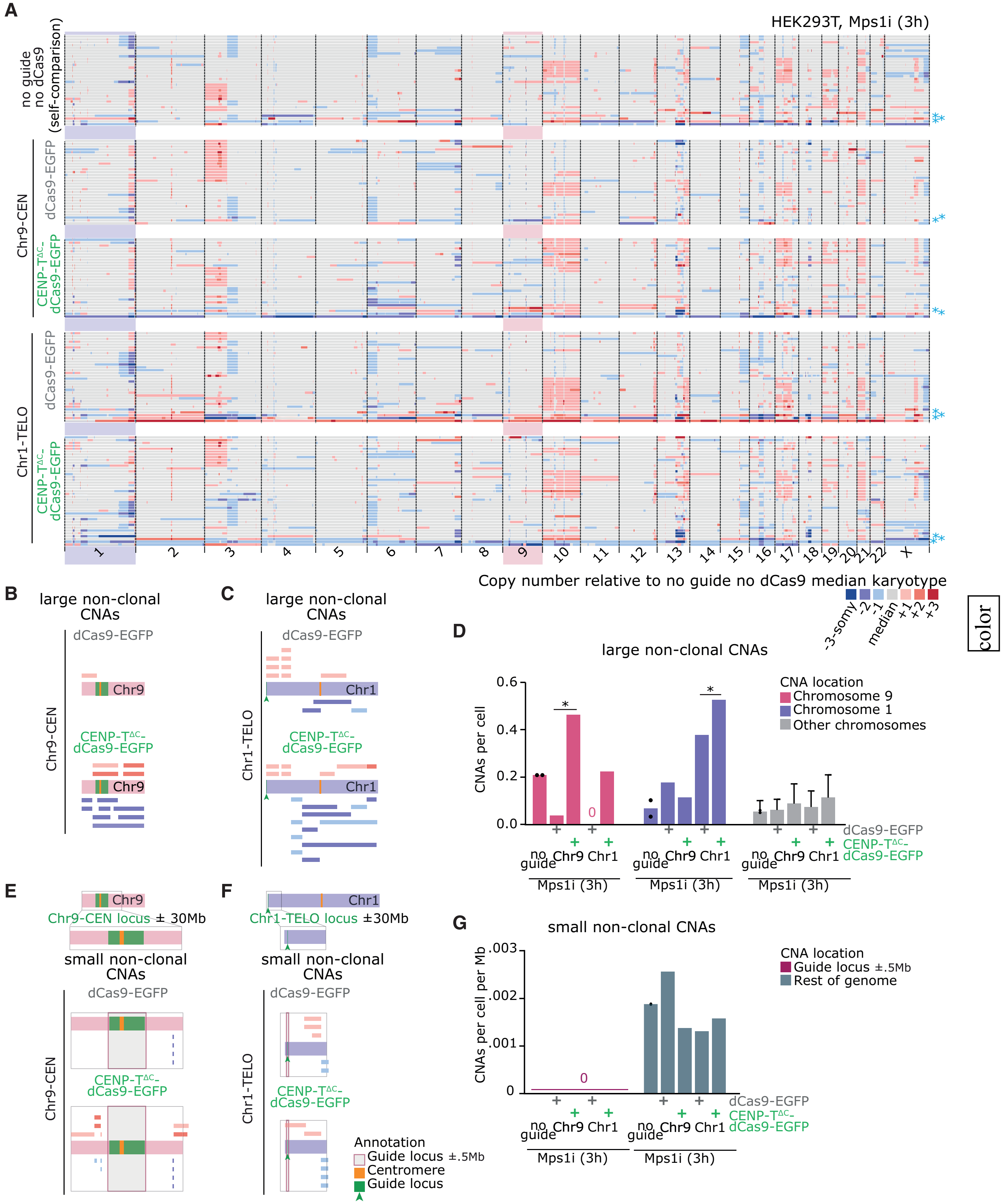

Figure 6.

© 2023 The Authors

Figure 6. Single cell sequencing reveals chromosome-specific aneuploidies induced by CENP-$T^{\Delta C}$-dCas9-nucleated kinetochores.

A Copy number calls from single cell sequencing data. Colours indicate copy number relative to the median karyotype of control HEK293T cells treated with Mps1i (no guide, no dCas9; top panel), in the conditions indicated. * = cells excluded from further analysis due to the presence of widespread aneuploidy relative to the median reference genome. Shaded boxes = target chromosomes.

B–G Assessment of nonclonal CNAs. See Fig EV4 and Materials and Methods for clonal and sub-clonal CNAs identification/removal details. (B, C) CNA pileups showing location of nonclonal large CNAs (> 20 Mb) in target chromosomes, for chromosome 9 (B) and chromosome 1 (C). (D) Frequency of nonclonal large CNAs (>20 Mb) per cell per chromosome, showing CNAs affecting chromosomes 9, 1, or other chromosomes. Bars = mean $\pm$ SD. Points = CNA rates for control removing clonal events from 2 different control sets (see Material and Methods; Fig EV4). *$P < 0.05$ (Chi-squared comparing the frequency of cells vs number of CNAs). (E, F) CNA pileups showing location of nonclonal small CNAs (< 20 Mb) in the guide target locus $\pm$30 Mb, for chromosome 9 (E) and chromosome 1 (F). (G) Frequency of nonclonal small CNAs (< 20 Mb) per cell, showing CNAs within the guide RNA locus $\pm$0.5 Mb vs the remainder of the genome.

Data information: Data in (A–G) is 28–39 cells sequenced per condition. Copy number state key applies to (A–C) and (E, F). In (B, C, E, F) orange lines = centromeres, green arrows/lines = guide RNA target locus. (E, F) Magenta box = guide locus $\pm$0.5 Mb window used for quantification in (G).

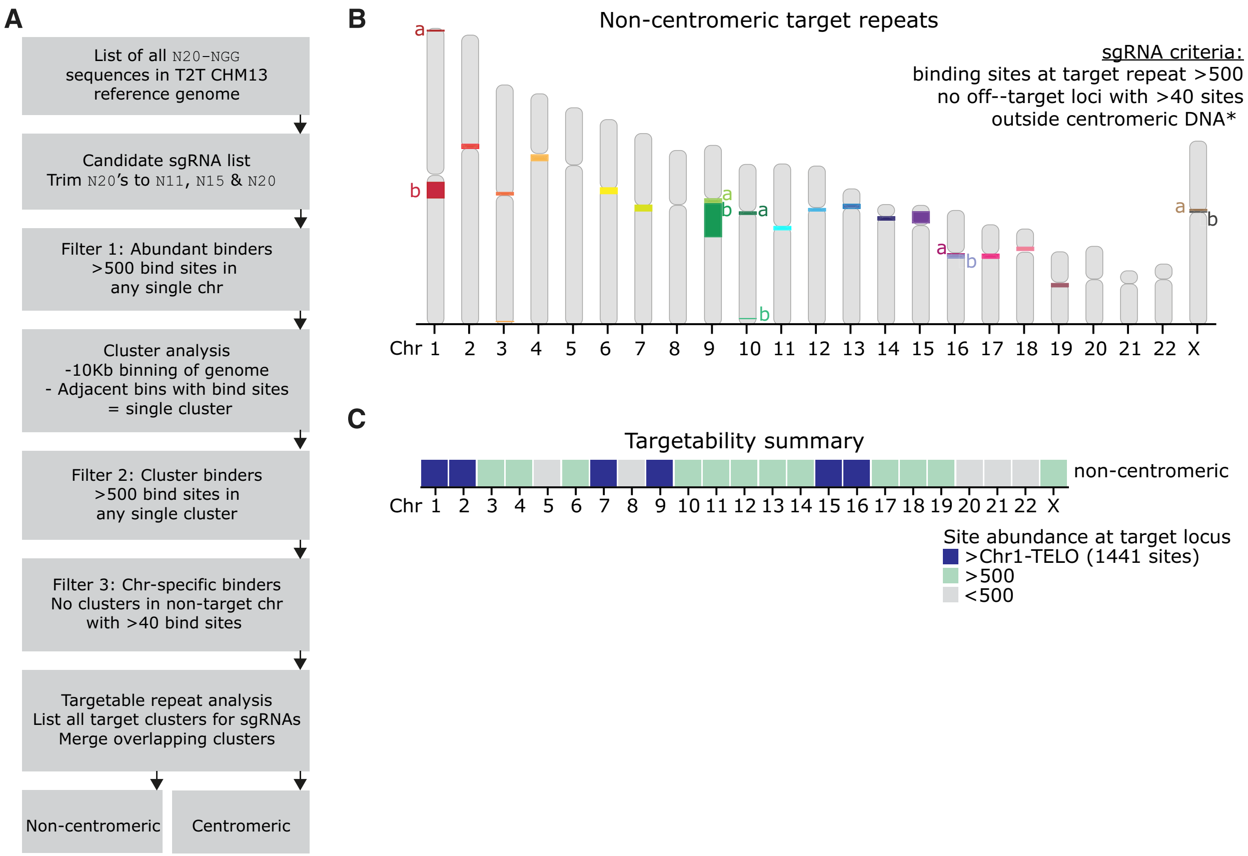

Figure 7. Putative guide RNAs for targeting noncentromeric repeats of 18 human chromosomes.

A Computational workflow for identification of guide RNAs. N, any nucleotide.

B Ideogram showing noncentromeric targetable repeats (i.e. with > 1 guide RNA meeting the listed criteria).

C Heatmap summarising the identified target repeats for each chromosome, coloured by site abundance for the guide RNA that has the highest number of binding sites.

However, we anticipate this could be a useful model to study the breakage of chromatin bridges, a common feature in cancer cells (Thompson & Compton, 2008; Bolhaqueiro *et al*, 2019; Tamura *et al*, 2020). Lastly, in our conditions, the dCas9 protein binding to repetitive regions during S-phase could have precipitated additional chromosomal alterations, even in the absence of CENP-$T^{\Delta C}$, as previously noted (Whinn *et al*, 2019), potentially explaining the increased target chromosome mis-segregation rates under this condition.

Future development of dCas9-based strategies to induce specific chromosome mis-segregation

Herein, we used transient transfections in HEK293T and HCT116 cells to allow rapid optimisation of the system, and to avoid the insertion of exogenous genomic material, therefore providing proof-of-principle of how specific chromosome segregation defects can be induced *ad hoc* in cell lines without any prior genetic editing required. However, we anticipate that generation of stable cell lines

© 2023 The Authors

with the capacity to create specifically targeted ectopic kinetochores at any locus, determined by the transfection with specific guide RNAs will provide a powerful model system to explore the cellular and genomic consequences of specific chromosome mis-segregation. Alternatively, flow-sorting of EGFP-positive cells, or baculoviral transduction of the constructs could be used to increase the efficiency in difficult to transfect cell lines (Hindriksen *et al*, 2017). To overcome the mitotic arrest, we took the approach of bypassing arrest using Mps1 inhibition. Herein, we used 1 μM, a dose that efficiently released arrest in HEK293T and HCT116 cells. This was the optimal dose balancing efficient release of arrest with lowest possible error rate. However, lower doses of Mps1i may be enough to release arrest in other cell types, which may reduce background error rates (Fig EV2G) so this should be determined in each cell line.

Materials and Methods

Cell culture and drug treatments

Cells were grown in DMEM (41966; Thermo Fisher Scientific) supplemented with 10% (v/v) Gibco™ Foetal Bovine Serum (FBS) (10500064; Thermo Fisher Scientific) and 1% (v/v) Penicillin–Streptomycin (#P4333; Sigma Aldrich) at 37°C and 5% CO_2. HEK293T cells were a gift from Prof. Victoria Sanz-Moreno, and HCT116 cells were a gift from Prof. Charles Swanton. Routine STR (Public Health England) and mycoplasma checks (#LT07-118; Lonza) were conducted to ensure cell line identification and mycoplasma-free status. For drug pulses (Figs 5 and EV2), cells were treated with 10 μM Aurora B inhibitor (ZM447439, Cayman chemical) or 1 μM Mps1 inhibitor (NMSP715; Sigma Aldrich) for 20 min for HCT116 and 30 min for HEK293T as was determined to be sufficient to allow metaphase arrested cells to reach anaphase. Mps1 inhibitor doses were lowered to 250 or 500 nM for titration experiments, using the same 20/30 min timings (Fig EV2F–H). For single cell sequencing (Figs 6 and EV4), a longer Mps1i treatment of 3 h was used. For microtubule depolymerisation experiments (Fig 3B), cells were treated with 100 ng/ml Nocodazole for 4 h.

Plasmids

For the generation of the CENP-T$^{\Delta C}$-dCas9-EGFP plasmid, the coding sequence for the CENP-T$^{\Delta C}$ fragment[6] was amplified from an existing plasmid (#45109; Addgene) for insertion into a dCas9-EGFP containing backbone (pHAGE-TO-dCas9-3XEGFP [#64107; Addgene]). In the amplification PCR for CENP-T$^{\Delta C}$, coding sequences for flexible linkers of 2xGlycine$_4$Serine were added on the primers such that they would flank CENP-T$^{\Delta C}$ in the fusion protein. For sgMUC4 and sgC9-CEN, a custom plasmid backbone was constructed by inserting an optimised guide RNA scaffold (Chen *et al*, 2013) under a U6 promoter into the mCherry-C1 plasmid (#54563; Addgene). Proceeding the guide RNA scaffold, a BsmBi-excisable sequence was included to allow for targeting sequence introduction as per the "Lentiviral CRISPR/Cas9 and single guide RNA" protocol from the Zhang laboratory (Joung *et al*, 2017). For live cell experiments using cells expressing H2B-RFP, sgC9-CEN was cloned into the lentiGuide-Puro plasmid that lacked the mCherry reporter (#52963; Addgene) by the same methodology. The plasmids generated in this study have been deposited in Addgene (product numbers to follow). sgChr1-TELO was a kind gift from Prof. Susanne Lens (Dumont *et al*, 2020). Targeting sequences for each guide RNA are detailed in Table 1.

Plasmid transfection

Plasmid expression was achieved by transient transfection of cells 24 h after plating with a dCas9:guide RNA plasmid ratio of 250:1,250 ng. The DNA solution and the transfection reagent (Lipofectamine 2000; Thermo Fisher Scientific) were separately prepared in Opti-MEM™ media (#31985062; Gibco) and mixed following the manufacturer's instructions. Transfection was performed by adding the transfection mix dropwise on the cell culture plates and cells harvested for further analysis 16–24 h later.

Immunofluorescence

Cells grown on glass slides or coverslips were fixed with PTEMF (0.2% Triton X-100, 0.02 M PIPES [pH 6.8], 0.01 M EGTA, 1 mM MgCl2, and 4% formaldehyde). After blocking with 3% BSA PBS for 1 h at 21°C, cells were incubated with primary antibodies in blocking buffer for 1 h at 21°C: 1:1,000 α-tubulin (118M4779V; Sigma Aldrich), 1:250 CREST (15-234-0001; Antibodies Incorporated), 1:500 CENP-T (ab220280; Abcam), 1:500 KNL-1 (ab222055; Abcam), 1:500 Mad2 (ab97777; Abcam), 1:500 Ndc80 (ma1-213308; Invitrogen). For Ndc80 staining, blocking was conducted instead in 5% Milk PBS. Secondary antibodies used were goat anti-human AF647 (109–606-088-JIR; Stratech), goat anti-rabbit AF594 (A11012; Invitrogen), goat anti-mouse AF594 (A11005; Invitrogen) and anti-mouse AF647 (115–605-003-JIR; Jackson Immunoresearch) at 1:250 dilution. DNA was stained with DAPI (Roche), and coverslips mounted in Vectashield (Vector H-1000; Vector Laboratories). Following each antibody incubation, cells were washed in PBS three times, each for 3 min.

Metaphase spreads

To enrich for mitotic cells, cells were treated with 0.1 μg/ml Colcemid (#15212012; ThermoFisher Scientific) for 2 h before conducting mitotic shake-off. Collected cells were pelleted and resuspended in 75 mM KCl hypotonic solution for 10–15 min depending on cell type, on ice. Cells were pelleted and resuspended in freshly prepared 3:1 methanol-glacial acetic acid a total of three times, before dropping onto slides.

Fluorescence *in situ* hybridization

Slides with freshly made metaphase spreads were put through an ethanol dehydration series and air dried. Cells and specific centromere probe (LPE 001R or LPE 009R; Cytocell) were denatured for 2 min at 75°C then incubated overnight at 37°C. The following day, slides were washed with 0.25X SSC at 72°C followed by a brief wash in 2X SSC, 0.05% Tween. DNA was stained for 6 min with 0.5 μg/ml DAPI (Roche) and coverslips mounted in Vectashield (Vector H-1000, Vector Laboratories).

Microscopy

Images were acquired using an Olympus DeltaVision RT microscope (Applied Precision, LLC) equipped with a Coolsnap HQ camera and

© 2023 The Authors

CO_2 controlled chamber. Three-dimensional image stacks were acquired at 0.2-μm intervals, using Olympus 100X (1.4 numerical aperture) UPlanSApo oil immersion objectives. Deconvolution of image stacks was performed with SoftWorxExplorer (Applied Precision, LLC). For live-cell imaging, HEK293T cells were seeded in four-well imaging dishes (Greiner Bio-one) and imaging begun 16 h following transfection. DNA was visualised either using Hoechst (Fig 3) or fluorescently labelled Histones (H2B-RFP; Fig 5). For Hoechst staining, cells were stained with 0.05 μg/ml Hoechst DNA staining solution (Thermo Fisher Scientific) for 10 min, then washed three times and imaged in freshly added media. For expression of H2B-RFP, cells were transduced with lentiviral preparations of the H2B-RFP plasmid (260001; Addgene) 24 h prior to transfection with the dCas9 and guide constructs. Cells were imaged using either an Olympus 40X 1.3 numerical aperture or Olympus 60X 1.4 numerical aperture UPlanSApo oil immersion objective, with image stacks at 2 μm intervals (10–12 images) taken every 3 min for 4–5 h. Analysis was performed using Softworx Explorer.

Analysis/measurements from EGFP foci

The intensity of the CENP-T and KNL-1 fluorescence signal (Figs 1 and 2) was measured using the Imaris software (Bitplane; version9.1/9.91). For each cell, the targeted EGFP signal and all endogenous centromeres were marked using automated detection, using spot-based detection based on anticentromere serum signal for each centromere, and surface-based detection for EGFP. Intensity measurements were taken for the channel of interest (either CENP-T or KNL-1) at these marked regions (targeted EGFP and endogenous centromeres). After subtracting average background intensity, intensity values were then normalised by dividing the average intensity at the EGFP focus by the average centromere intensity (using values from the same cell). EGFP stretch measurements (Fig 3C) were also conducted in Imaris, again using surface-based detection for EGFP and taking the Ellipsoid axis C metric as the length. Attachment status of ectopic kinetochores (Fig 4) was assessed using the Imaris software to allow for visualisation of microtubules in 3D.

Single-cell sequencing and analysis

Single G1 nuclei, as assessed by PI and Hoechst staining, were isolated by flow cytometric cell sorting into 96-well plates and pre-amplification-free single-cell whole-genome sequencing libraries prepared using a Bravo automated liquid handling platform (Agilent Technologies) as previously described (van den Bos *et al*, 2016, 2019). In brief, genomic DNA was fragmented using micrococcal nuclease followed by end-repair, A-tailing and Illumina PE forked adapter ligation. Upon AMPure XP bead clean-up, the adapter-containing DNA fragments were subjected to PCR amplification using multiplexing primers to incorporate library-specific barcodes. Pooled libraries were subsequently shallow sequenced on an Illumina NextSeq 500 sequencer (up to 77 cycles; single end) with ~ 1% genomic DNA coverage. The generated data were demultiplexed using library-specific barcodes and changed into fastq files using bcl2fastq (Illumina; version 1.8.4). Reads were afterwards aligned to the human reference genome (GRCh38/hg38) using Bowtie2 (version 2.2.4; Langmead & Salzberg, 2012). Duplicate reads were marked with BamUtil (version 1.0.3; Jun *et al*, 2015).

Copy number calls were made using AneuFinder (version1.14.036). To account for the high level of existing heterogeneity seen in controls, modal copy number calls were calculated by taking the median copy number per 500 kb bin across the individual cells (HEK293T, Mps1i (3 h), no guide no dCas9; Fig EV3A). Copy number states for the single cells from other conditions were then recalculated relative to this median reference (Fig 6A). For isolation of nonclonal CNAs, clonal (or subclonal) CNAs were identified from control conditions and removed from test conditions using ClonalMasker. To allow detection of rarer subclonal events, control CNA conditions were chosen to include all conditions in which the test guide RNA was not used, these were as follows: Chr1 (no guide/dCas9-EGFP Chr9/CENP- $T^{\Delta C}$-dCas9-EGFP Chr9), Chr 9 (no guide/dCas9-EGFP Chr1/CENP-$T^{\Delta C}$-dCas9-EGFP Chr1), no guide (either the controls used for Chr1, or Chr9 to allow for direct comparison vs those conditions). In ClonalMasker, a minimum overlap of 50% was utilised, and a fraction that secured only CNAs present in at least four cells was called (4/83 = 0.46). Pileup graphs were then created from this filtering and include only nonclonal CNAs that have breakpoints greater than 1 Mb apart (Figs 6B and EV4B–D). Analysis was carried out in Excel and R (version 4.1.1, R Foundation for Statistical Computing) using RStudio interface, including separation of CNAs based on size. Clonal CNA filtering scripts are available at https://github.com/MBoemo/clonalMasker.New.

Estimation of guide RNA-binding site abundancy

Guide RNA-binding site predictions were run using the command line version of Cas-OFFinder algorithm (Bae *et al*, 2014) against the T2T-CHM13 (version 1.1) reference genome (Nurk *et al*, 2022). For generation of plots, chromosome reference sequences were binned into 10Kbp lengths, and any adjacent bins with one or more binding sites predicted were then merged to form a single binding cluster. The number of predicted binding sites was then calculated per cluster, and the details of the cluster with the most binding sites reported (Table.1). Genome binning and cluster analysis were conducted in R (version 4.1.1, R Foundation for Statistical Computing) using RStudio interface.

Identification of targetable repeats in the human genome

All PAM sites (of the sequence NGG) in the T2T CHM13 v1.1 reference genome were identified using CRISPR-OR (Concordet & Haeussler, 2018). To allow for a range of guide RNA lengths, the corresponding guide RNA sequences (of the sequence N20-NGG) were trimmed to N11-NGG or N15-NGG. Guide RNAs were then put through three successive filter steps to retain: (i) guides with > 500 predicted bind sites in any one chromosome, (ii) guides with > 500 predicted bind sites in any single cluster and (iii) guides with no clusters in nontarget chromosomes with > 40 bind sites (i.e. chromosome specific). Clusters were identified as described for estimation of guide bind site abundance, considering only bind site predictions with no mis-matches or bulges. A guide RNAs target cluster was then defined as the site at which it had the most predicted bind sites. From the list of 2,471 guide RNAs, a list of all their target clusters was compiled, and any overlapping clusters were combined into a single targetable repeat. Targetable repeats were then classified as centromeric if the co-ordinates overlapped

with either the CHM13 centromere annotations, or the CENP-A binding peaks (Altemose *et al*, 2022).

Preparation of data and figures

All graphs were prepared, and statistical testing performed in the Prism software (version 9.0, GraphPad). Cropping of final images was performed in Photoshop (Adobe Photoshop CS6 2018, USA). All figures were assembled in Illustrator (Adobe Illustrator CS6 2018).

Data availability

All raw sequencing data are accessible via the European Nucleotide Archive with accession ID PRJEB59859 (https://www.ebi.ac.uk/ena/browser/view/PRJEB59859). All plasmids generated in this study have been made available on Addgene (empty sgRNA, sgC9cen, sgMUC4 and CENP-T$^{\Delta C}$-dCas9-EGFP plasmids; 198330, 198399, 198326 and 198325, respectively).

Expanded View for this article is available online.

Acknowledgements
We would like to thank Susanne Lens, My Anh Truong, Paula Cané-Gasull and Sippe de Vries for helpful discussions and sharing of unpublished data and reagents. We would also like to thank Iain Cheeseman for constructs (via Addgene) and constructive discussions, Steve Royle for discussions and advice and Victoria Sanz-Moreno for HEK293T cells. We also kindly thank Karen Miga for providing us with the list of PAM-containing sgRNA binding sites in the T2T Human genome reference. We would also like to dedicate this work to the memory of Norah Reed, founder of the Barry Reed Charity, whose generosity and genuine interest in the scientific findings and career progression of LT was an inspiration to us. LT was funded by Barry Reed PhD studentship and CRUK Pioneer award C35980/A27846. SCJ was funded by an MRC PhD studentship and BBSRC grant BB/V009605/1. AA was supported by a Bowel Cancer UK grant. MG was funded by an MRC PhD studentship. FF and RW were funded by the Dutch Cancer Society grant 2018-RUG-11457. LT, SCJ and SEM acknowledge funding from a Cancer Research UK Centre Grant C355/A25137.

Author contributions
Laura Tovini: Data curation; formal analysis; investigation; methodology; writing – original draft; writing – review and editing. **Sarah C Johnson:** Data curation; formal analysis; investigation; methodology; writing – original draft; writing – review and editing. **Molly A Guscott:** Formal analysis. **Alexander M Andersen:** Formal analysis. **Diana CJ Spierings:** Investigation. **René Wardenaar:** Formal analysis. **Floris Foijer:** Formal analysis; supervision. **Sarah E McClelland:** Conceptualization; formal analysis; supervision; funding acquisition; investigation; writing – original draft; project administration; writing – review and editing.

Disclosure and competing interests statement
The authors declare that they no conflict of interest.

References

Altemose N, Logsdon GA, Bzikadze AV, Sidhwani P, Langley SA, Caldas GV, Hoyt SJ, Uralsky L, Ryabov FD, Shew CJ *et al* (2022) Complete genomic and epigenetic maps of human centromeres. *Science* 376: eabl4178

Bae S, Park J, Kim JS (2014) Cas-OFFinder: a fast and versatile algorithm that searches for potential off-target sites of Cas9 RNA-guided endonucleases. *Bioinformatics* 30: 1473 – 1475

Baker D, Hirst AJ, Gokhale PJ, Juarez MA, Williams S, Wheeler M, Bean K, Allison TF, Moore HD, Andrews PW *et al* (2016) Detecting genetic mosaicism in cultures of human pluripotent stem cells. *Stem Cell Reports* 7: 998 – 1012

Binz RL, Tian E, Sadhukhan R, Zhou D, Hauer-Jensen M, Pathak R (2019) Identification of novel breakpoints for locus- and region-specific translocations in 293 cells by molecular cytogenetics before and after irradiation. *Sci Rep* 9: 10554

Bolhaqueiro ACF, Ponsioen B, Bakker B, Klaasen SJ, Kucukkose E, van Jaarsveld RH, Vivie J, Verlaan-Klink I, Hami N, Spierings DCJ *et al* (2019) Ongoing chromosomal instability and karyotype evolution in human colorectal cancer organoids. *Nat Genet* 51: 824 – 834

van den Bos H, Spierings DC, Taudt AS, Bakker B, Porubsky D, Falconer E, Novoa C, Halsema N, Kazemier HG, Hoekstra-Wakker K *et al* (2016) Single-cell whole genome sequencing reveals no evidence for common aneuploidy in normal and Alzheimer's disease neurons. *Genome Biol* 17: 116

van den Bos H, Bakker B, Taudt A, Guryev V, Colome-Tatche M, Lansdorp PM, Foijer F, Spierings DCJ (2019) Quantification of aneuploidy in mammalian systems. *Methods Mol Biol* 1896: 159 – 190

Bosco N, Goldberg A, Zhao X, Mays JC, Cheng P, Johnson AF, Bianchi JJ, Toscani C, Tommaso E, Katsnelson L *et al* (2023) KaryoCreate: a CRISPR-based technology to study chromosome-specific aneuploidy by targeting human centromeres. *Cell* https://doi.org/10.1016/j.cell.2023

Breems DA, Van Putten WL, De Greef GE, Van Zelderen-Bhola SL, Gerssen-Schoorl KB, Mellink CH, Nieuwint A, Jotterand M, Hagemeijer A, Beverloo HB *et al* (2008) Monosomal karyotype in acute myeloid leukemia: a better indicator of poor prognosis than a complex karyotype. *J Clin Oncol* 26: 4791 – 4797

Brinkley BR, Cartwright J Jr (1975) Cold-labile and cold-stable microtubules in the mitotic spindle of mammalian cells. *Ann N Y Acad Sci* 253: 428 – 439

Chen B, Gilbert LA, Cimini BA, Schnitzbauer J, Zhang W, Li GW, Park J, Blackburn EH, Weissman JS, Qi LS *et al* (2013) Dynamic imaging of genomic loci in living human cells by an optimized CRISPR/Cas system. *Cell* 155: 1479 – 1491

Chunduri NK, Menges P, Zhang X, Wieland A, Gotsmann VL, Mardin BR, Buccitelli C, Korbel JO, Willmund F, Kschischo M *et al* (2021) Systems approaches identify the consequences of monosomy in somatic human cells. *Nat Commun* 12: 5576

Cimini D, Howell B, Maddox P, Khodjakov A, Degrassi F, Salmon ED (2001) Merotelic kinetochore orientation is a major mechanism of aneuploidy in mitotic mammalian tissue cells. *J Cell Biol* 153: 517 – 528

Colombo R, Caldarelli M, Mennecozzi M, Giorgini ML, Sola F, Cappella P, Perrera C, Depaolini SR, Rusconi L, Cucchi U *et al* (2010) Targeting the mitotic checkpoint for cancer therapy with NMS-P715, an inhibitor of MPS1 kinase. *Cancer Res* 70: 10255 – 10264

Concordet J-P, Haeussler M (2018) CRISPOR: intuitive guide selection for CRISPR/Cas9 genome editing experiments and screens. *Nucleic Acids Res* 46: W242 – W245

Ditchfield C, Johnson VL, Tighe A, Ellston R, Haworth C, Johnson T, Mortlock A, Keen N, Taylor SS (2003) Aurora B couples chromosome alignment with anaphase by targeting BubR1, Mad2, and Cenp-E to kinetochores. *J Cell Biol* 161: 267 – 280

© 2023 The Authors

Dumont M, Gamba R, Gestraud P, Klaasen S, Worrall JT, De Vries SG, Boudreau V, Salinas-Luypaert C, Maddox PS, Lens SM *et al* (2020) Human chromosome-specific aneuploidy is influenced by DNA-dependent centromeric features. *EMBO J* 39: e102924

Gascoigne KE, Cheeseman IM (2013) Induced dicentric chromosome formation promotes genomic rearrangements and tumorigenesis. *Chromosome Res* 21: 407 – 418

Gascoigne KE, Takeuchi K, Suzuki A, Hori T, Fukagawa T, Cheeseman IM (2011) Induced ectopic kinetochore assembly bypasses the requirement for CENP-A nucleosomes. *Cell* 145: 410 – 422

Girish V, Lakhani AA, Scaduto CM, Thompson SL, Brown LM, Hagenson RA, Sausville EL, Mendelson BE, Lukow DA, Yuan ML *et al* (2023) Oncogene-like addiction to aneuploidy in human cancers. *bioRxiv* https://doi.org/10.1101/2023.01.09.523344 [PREPRINT]

Gisselsson D, Pettersson L, Höglund M, Heidenblad M, Gorunova L, Wiegant J, Mertens F, Dal Cin P, Mitelman F, Mandahl N (2000) Chromosomal breakage-fusion-bridge events cause genetic intratumor heterogeneity. *Proc Natl Acad Sci USA* 97: 5357 – 5362

Gurden MD, Anderhub SJ, Faisal A, Linardopoulos S (2018) Aurora B prevents premature removal of spindle assembly checkpoint proteins from the kinetochore: a key role for Aurora B in mitosis. *Oncotarget* 9: 19525 – 19542

Hara M, Ariyoshi M, Okumura EI, Hori T, Fukagawa T (2018) Multiple phosphorylations control recruitment of the KMN network onto kinetochores. *Nat Cell Biol* 20: 1378 – 1388

Hauf S, Cole RW, LaTerra S, Zimmer C, Schnapp G, Walter R, Heckel A, van Meel J, Rieder CL, Peters JM (2003) The small molecule Hesperadin reveals a role for Aurora B in correcting kinetochore-microtubule attachment and in maintaining the spindle assembly checkpoint. *J Cell Biol* 161: 281 – 294

Hayward D, Roberts E, Gruneberg U (2022) MPS1 localizes to end-on microtubule-attached kinetochores to promote microtubule release. *Curr Biol* 32: 5200 – 5208.e8

Hengeveld RCC, Vromans MJM, Vleugel M, Hadders MA, Lens SMA (2017) Inner centromere localization of the CPC maintains centromere cohesion and allows mitotic checkpoint silencing. *Nat Commun* 8: 15542

Hindriksen S, Bramer AJ, Truong MA, Vromans MJM, Post JB, Verlaan-Klink I, Snippert HJ, Lens SMA, Hadders MA (2017) Baculoviral delivery of CRISPR/Cas9 facilitates efficient genome editing in human cells. *PloS one* 12: e0179514

Hori T, Amano M, Suzuki A, Backer CB, Welburn JP, Dong Y, McEwen BF, Shang WH, Suzuki E, Okawa K *et al* (2008) CCAN makes multiple contacts with centromeric DNA to provide distinct pathways to the outer kinetochore. *Cell* 135: 1039 – 1052

Hori T, Shang WH, Takeuchi K, Fukagawa T (2013) The CCAN recruits CENP-A to the centromere and forms the structural core for kinetochore assembly. *J Cell Biol* 200: 45 – 60

Hutaff-Lee C, Cordeiro L, Tartaglia N (2013) Cognitive and medical features of chromosomal aneuploidy. *Handb Clin Neurol* 111: 273 – 279

Jacome A, Fernandez-Capetillo O (2011) Lac operator repeats generate a traceable fragile site in mammalian cells. *EMBO Rep* 12: 1032 – 1038

Joung J, Konermann S, Gootenberg JS, Abudayyeh OO, Platt RJ, Brigham MD, Sanjana NE, Zhang F (2017) Genome-scale CRISPR-Cas9 knockout and transcriptional activation screening. *Nat Protoc* 12: 828 – 863

Jun G, Wing MK, Abecasis GR, Kang HM (2015) An efficient and scalable analysis framework for variant extraction and refinement from population-scale DNA sequence data. *Genome Res* 25: 918 – 925

Klaasen SJ, Truong MA, van Jaarsveld RH, Koprivec I, Stimac V, de Vries SG, Risteski P, Kodba S, Vukusic K, de Luca KL *et al* (2022) Nuclear chromosome locations dictate segregation error frequencies. *Nature* 607: 604 – 609

Kooi IE, Mol BM, Massink MP, de Jong MC, de Graaf P, van der Valk P, Meijers-Heijboer H, Kaspers GJ, Moll AC, Te Riele H *et al* (2016) A meta-analysis of retinoblastoma copy numbers refines the list of possible driver genes involved in tumor progression. *PloS one* 11: e0153323

Krivega M, Stiefel CM, Karbassi S, Andersen LL, Chunduri NK, Donnelly N, Pichlmair A, Storchova Z (2021) Genotoxic stress in constitutive trisomies induces autophagy and the innate immune response via the cGAS-STING pathway. *Commun Biol* 4: 831

Kuhl LM, Makrantoni V, Recknagel S, Vaze AN, Marston AL, Vader G (2020) A dCas9-based system identifies a central role for Ctf19 in kinetochore-derived suppression of meiotic recombination. *Genetics* 216: 395 – 408

Langmead B, Salzberg SL (2012) Fast gapped-read alignment with bowtie 2. *Nat Methods* 9: 357 – 359

Leibowitz ML, Papathanasiou S, Doerfler PA, Blaine LJ, Sun L, Yao Y, Zhang CZ, Weiss MJ, Pellman D (2021) Chromothripsis as an on-target consequence of CRISPR-Cas9 genome editing. *Nat Genet* 53: 895 – 905

Ma H, Naseri A, Reyes-Gutierrez P, Wolfe SA, Zhang S, Pederson T (2015) Multicolor CRISPR labeling of chromosomal loci in human cells. *Proc Natl Acad Sci U S A* 112: 3002 – 3007

Mackinnon RN, Campbell LJ (2011) The role of dicentric chromosome formation and secondary centromere deletion in the evolution of myeloid malignancy. *Genet Res Int* 2011: 643628

Nollet S, Moniaux N, Maury J, Petitprez D, Degand P, Laine A, Porchet N, Aubert JP (1998) Human mucin gene MUC4: organization of its 5′-region and polymorphism of its central tandem repeat array. *Biochem J* 332: 739 – 748

Nurk S, Koren S, Rhie A, Rautiainen M, Bzikadze AV, Mikheenko A, Vollger MR, Altemose N, Uralsky L, Gershman A *et al* (2022) The complete sequence of a human genome. *Science* 376: 44 – 53

Passerini V, Ozeri-Galai E, de Pagter MS, Donnelly N, Schmalbrock S, Kloosterman WP, Kerem B, Storchova Z (2016) The presence of extra chromosomes leads to genomic instability. *Nat Commun* 7: 10754

Qin P, Parlak M, Kuscu C, Bandaria J, Mir M, Szlachta K, Singh R, Darzacq X, Yildiz A, Adli M (2017) Live cell imaging of low- and non-repetitive chromosome loci using CRISPR-Cas9. *Nat Commun* 8: 14725

Rago F, Gascoigne KE, Cheeseman IM (2015) Distinct organization and regulation of the outer kinetochore KMN network downstream of CENP-C and CENP-T. *Curr Biol* 25: 671 – 677

Santaguida S, Vernieri C, Villa F, Ciliberto A, Musacchio A (2011) Evidence that Aurora B is implicated in spindle checkpoint signalling independently of error correction. *EMBO J* 30: 1508 – 1519

Saunderson EA, Stepper P, Gomm JJ, Hoa L, Morgan A, Allen MD, Jones JL, Gribben JG, Jurkowski TP, Ficz G (2017) Hit-and-run epigenetic editing prevents senescence entry in primary breast cells from healthy donors. *Nat Commun* 8: 1450

Shaikh N, Mazzagatti A, De Angelis S, Johnson SC, Bakker B, Spierings DCJ, Wardenaar R, Maniati E, Wang J, Boemo MA *et al* (2022) Replication stress generates distinctive landscapes of DNA copy number alterations and chromosome scale losses. *Genome Biol* 23: 223

Stanyte R, Nuebler J, Blaukopf C, Hoefler R, Stocsits R, Peters JM, Gerlich DW (2018) Dynamics of sister chromatid resolution during cell cycle progression. *J Cell Biol* 217: 1985 – 2004

Tamura N, Shaikh N, Muliaditan D, Soliman TN, McGuinness JR, Maniati E, Moralli D, Durin MA, Green CM, Balkwill FR *et al* (2020) Specific

© 2023 The Authors

mechanisms of chromosomal instability indicate therapeutic sensitivities in high-grade serous ovarian carcinoma. *Cancer Res* 80: 4946 – 4959

Tanenbaum ME, Gilbert LA, Qi LS, Weissman JS, Vale RD (2014) A protein-tagging system for signal amplification in gene expression and fluorescence imaging. *Cell* 159: 635 – 646

Taylor AM, Shih J, Ha G, Gao GF, Zhang X, Berger AC, Schumacher SE, Wang C, Hu H, Liu J *et al* (2018) Genomic and functional approaches to understanding cancer aneuploidy. *Cancer Cell* 33: 676 – 689.e3

Thompson SL, Compton DA (2008) Examining the link between chromosomal instability and aneuploidy in human cells. *J Cell Biol* 180: 665 – 672

Tighe A, Staples O, Taylor S (2008) Mps1 kinase activity restrains anaphase during an unperturbed mitosis and targets Mad2 to kinetochores. *J Cell Biol* 181: 893 – 901

Truong MA, Cane-Gasull P, de Vries SG, Nijenhuis W, Wardenaar R, Kapitein LC, Foijer F, Lens SMA (2023) A kinesin-based approach for inducing chromosome-specific mis-segregation in human cells. *EMBO J* 42: e111559

Umbreit NT, Zhang CZ, Lynch LD, Blaine LJ, Cheng AM, Tourdot R, Sun L, Almubarak HF, Judge K, Mitchell TJ *et al* (2020) Mechanisms generating cancer genome complexity from a single cell division error. *Science* 368: eaba0712

Waters JC, Chen R-H, Murray AW, Salmon ED (1998) Localization of Mad2 to kinetochores depends on microtubule attachment, not tension. *J Cell Biol* 141: 1181 – 1191

Whinn KS, Kaur G, Lewis JS, Schauer GD, Mueller SH, Jergic S, Maynard H, Gan ZY, Naganbabu M, Bruchez MP *et al* (2019) Nuclease dead Cas9 is a programmable roadblock for DNA replication. *Sci Rep* 9: 13292

License: This is an open access article under the terms of the Creative Commons Attribution License, which permits use, distribution and reproduction in any medium, provided the original work is properly cited.

 © 2023 The Authors

Expanded View Figures

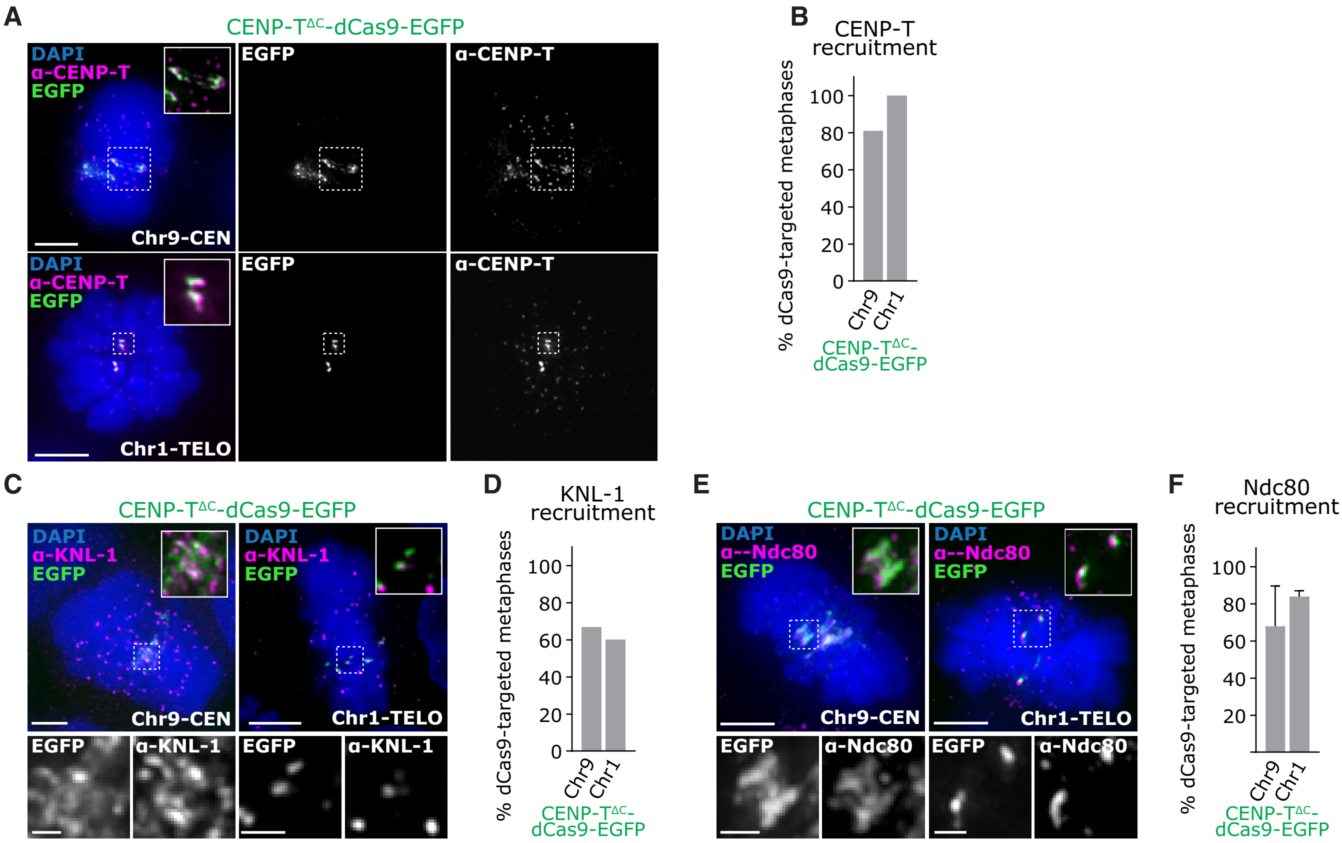

Figure EV1. related to Fig 2. CENP-T$^{\Delta C}$-dCas9 targeting recruits KNL-1 and Ndc80 to large repetitive chromosomal loci in HCT116 cells.

A Immunofluorescence images of HCT116 cells with CENP-T$^{\Delta C}$-dCas9-EGFP targeted to Chr9-CEN or Chr1-TELO, stained with antibodies against CENP-T.

B Percentage of metaphase cells showing EGFP and CENP-T signal co-localisation. ≥ 20 metaphases analysed per condition, 1 experiment.

C Immunofluorescence images of HCT116 cells with CENP-T$^{\Delta C}$-dCas9-EGFP targeted to Chr9-CEN or Chr1-TELO, stained with antibodies against KNL-1.

D Percentage of metaphase cells showing EGFP and KNL-1 signal co-localisation. ≥ 20 metaphases analysed per condition, 1 experiment.

E Immunofluorescence images of HCT116 cells with CENP-T$^{\Delta C}$-dCas9-EGFP targeted to Chr9-CEN or Chr1-TELO, stained with antibodies against Ndc80.

F Percentage of metaphase cells showing colocalization of EGFP with Ndc80. ≥ 20 metaphases counted per condition, in each of 2 experiments, Bars = mean ± SD.

Data information: Images in (A, C, E) are maximum intensity projections taken across the depth of the EGFP foci. Scale bars = 5 μm on large images, 1 μm on zooms.

© 2023 The Authors

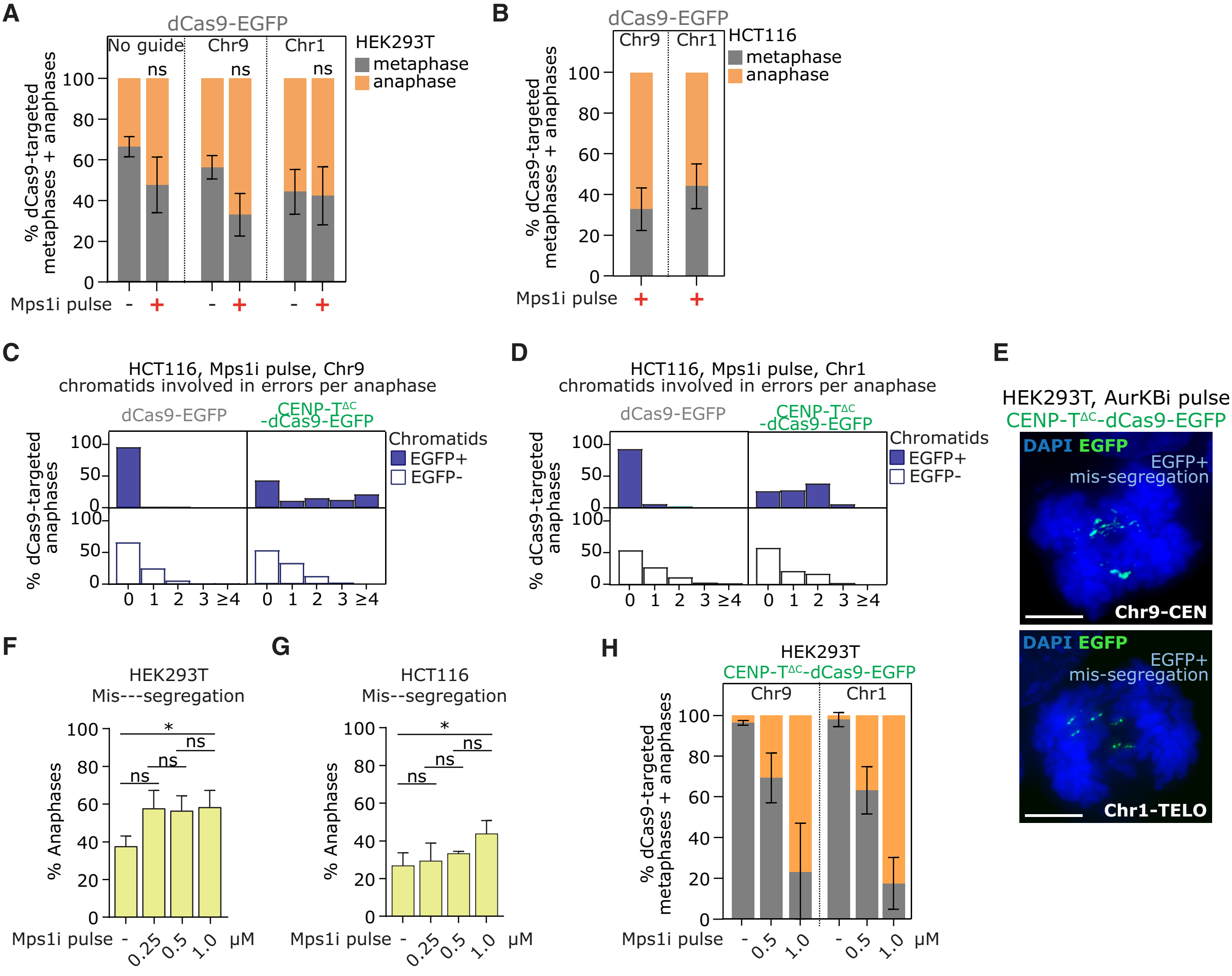

Figure EV2. related to Fig 5. Lowering Mps1 inhibitor dose does not improve off-target chromosome mis-segregation.

A, B Quantification of mitotic stage from fixed dCas9-EGFP targeted HEK293T (A) or HCT116 (B) cells following Mps1i pulse.

C, D Quantification of number of EGFP+ or EGFP- chromatids involved in segregation errors from HCT116 anaphases with dCas9 targeting.

E Immunofluorescence images of anaphase HEK293T cells with CENP-TΔC-dCas9 EGFP targeting after 30 min AurKBi treatment, showing examples of EGFP+ mis segregation. Maximum intensity projection taken across the depth of the EGFP foci. Scale bars = 5 μm.

F, G Mis-segregation rate in untransfected HEK293T (F) or HCT116 (G) cells following Mps1i pulse at 0.25, 0.5 or 1 μM. $^{ns}P > 0.05$, $^{*}P < 0.05$ (One way ANOVA with Šidák's multiple comparison correction).

H Quantification of mitotic stage from fixed CENP T$^{\Delta C}$-dCas9-EGFP targeted HEK293T cells after a pulse or Mps1i at 0.5 or 1 μM. ≥ 50 metaphases + anaphases analysed per condition, in each of 2 experiments.

Data information: Data in (A, B, H) are from ≥ 40 metaphase + anaphases per condition, in each of 3 experiments for (A, B) and 2 experiments for (H). $^{ns}P > 0.05$ (One-way ANOVA with Šidák's multiple comparison correction). Bars = mean ± SD. Data in (C, D, F, G) are from ≥ 25 anaphases analysed per condition, in each of ≥ 3 experiments. In (F, G) Bars = mean + SD.

© 2023 The Authors

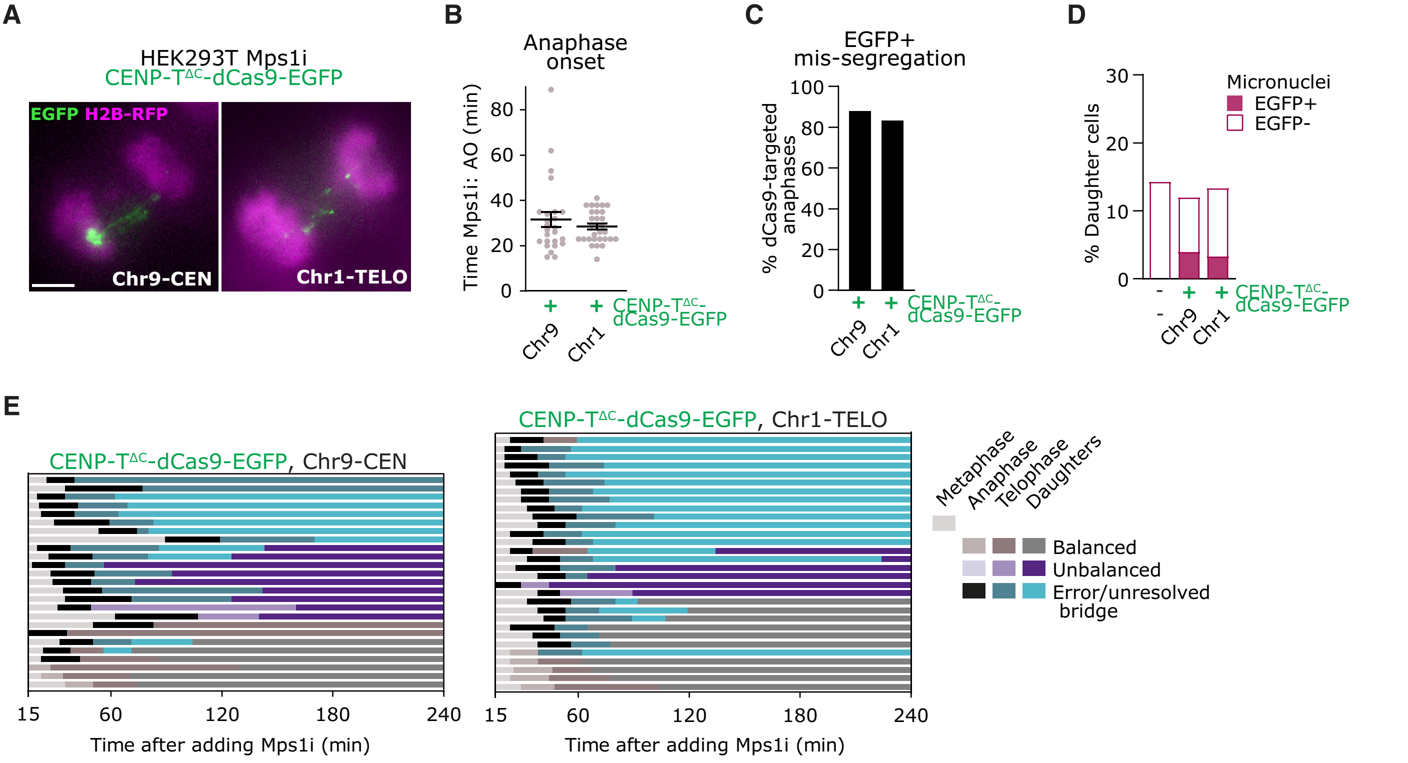

Figure EV3. related to Fig 5. Tracking the fate of mis-segregated target chromosomes by live cell imaging.

A Frames from live cell imaging of HEK293T H2B-RFP cells after treatment with Mps1i, with CENP-T$^{\Delta C}$-dCas9-EGFP targeted to Chr9-CEN or Chr1-TELO, showing examples of EGFP+ segregation errors. Scale bar = 5 μm.

B Time from addition of Mps1i to anaphase onset (AO), for CENPT$^{\Delta C}$dCas9-EGFP targeted HEK293T H2B-RFP cells that were in metaphase when Mps1i was added. Lines = mean ± SEM. Each point = 1 cell.

C Mis-segregation rate in CENP-T$^{\Delta C}$-dCas9-EGFP targeted HEK293T H2B-RFP daughter cells assessed by live cell imaging.

D Percentage of daughter cells with micronuclei, classified as EGFP+ or EGFP-.

E Cell cycle stages of cells with CENP-T$^{\Delta C}$-dCas9-EGFP targeting, by live-cell imaging (each line = 1 cell). Cell cycle stages have been sub-categorised based on EGFP state, ie. balanced/unbalanced distribution between daughter cells/anaphase or telophase fronts, and involvement in errors or unresolved bridges.

Data information: Data in (B, C, D, E) are from ≥ 25 cells tracked per condition, compiled from ≥3 independent experiments.

Figure EV4. related to Fig 6. Additional data from single cell sequencing experiments.

A Heatmap showing copy number alterations (CNAs) in single HEK293T cells treated with Mps1i. Heterogeneity is seen at the whole, and partial chromosome level between cells. Median ploidy was calculated across the genome to produce a median karyotype (lower heatmap).

B, C CNA pileups indicating the CNAs present in each condition, after filtering out any clonal or subclonal CNAs present in control conditions. Copy number gains are indicated in red (pale red = +1, red = +2, dark red = +3 copies above median reference) and losses in blue (pale blue = −1, blue = −2, dark blue = −3 copies below reference). Orange lines indicate centromeres and green lines indicate CENP-T$^{\Delta C}$ target sites.

D Nonclonal large CNAs (> 20 Mb) calculated per chromosome per cell for each condition indicated. Points shown for no guide condition = CNA rates after filtering sub-clonal events from the 2 different control sets.

▶

© 2023 The Authors

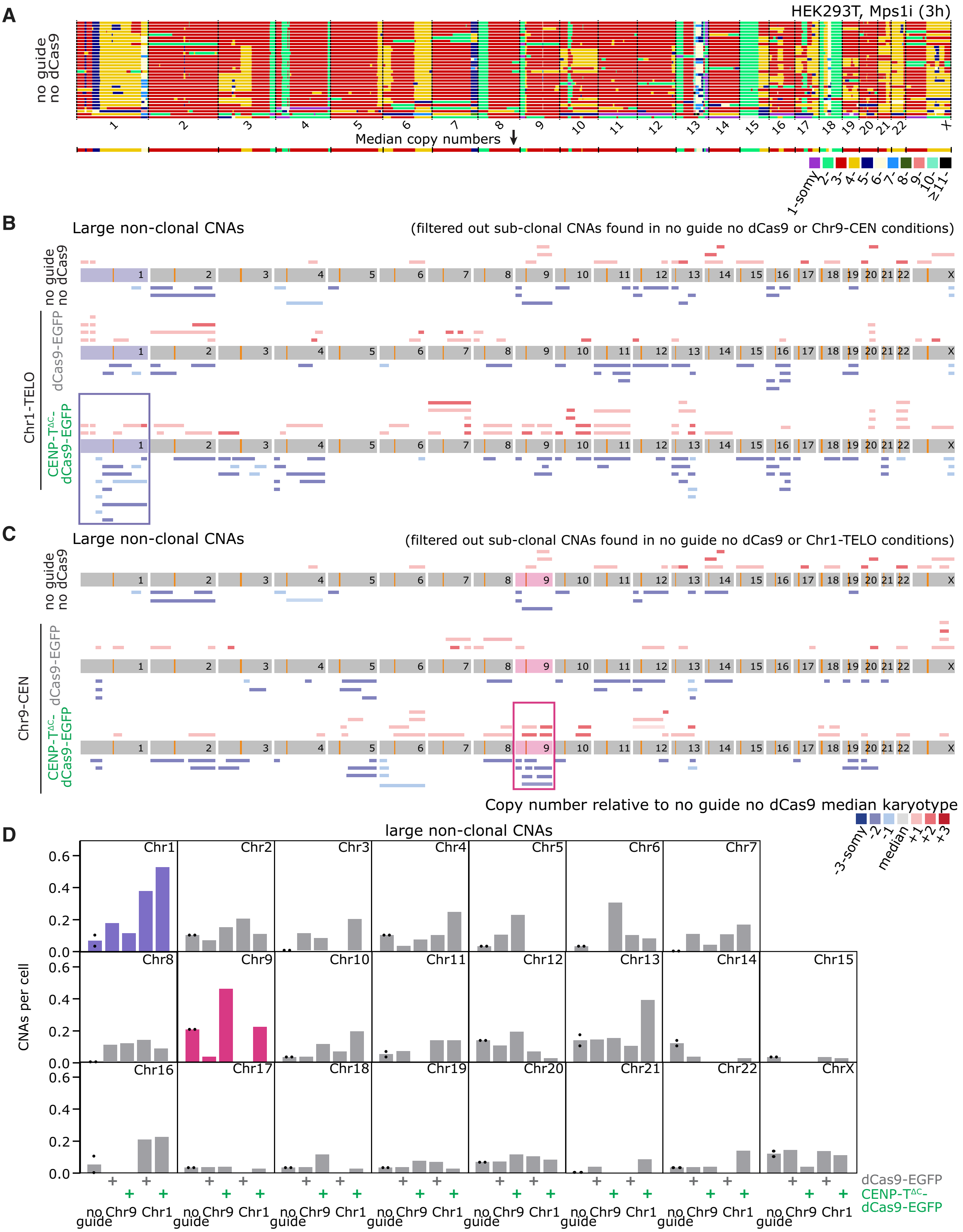

Figure EV4.

© 2023 The Authors

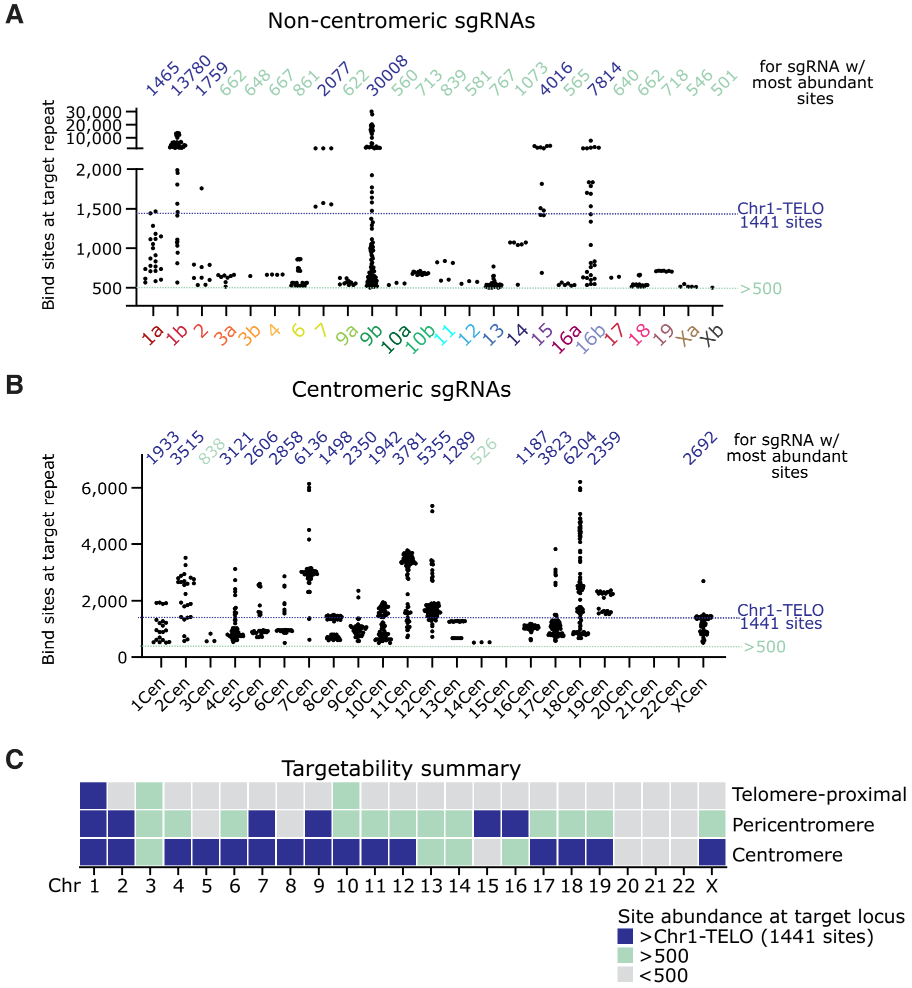

Figure EV5. related to Fig 7. Further details of putative guide RNAs for targeting repeats, including centromeres.

A, B Number of predicted binding sites at the target locus for each guide RNA. Each point = 1 guide RNA. Guides have been grouped by their target repeat and separated into those targeting noncentromeric (A) or centromeric (B) repeats. Numbers at the top of the plots = predicted binding sites at the target repeat for the guide RNA with the highest number of binding sites. Nomenclature for repeats is Cen = centromere, a, b = noncentromeric starting alphabetically at p-telomere.

C Heatmap summarising the identified target repeats for each chromosome, coloured by site abundance for the guide RNA that has the highest number of binding sites. Noncentromeric repeats have been further subcategorised into those proximal to the centromere (Pericentromere) or telomere (Telomere-proximal).

© 2023 The Authors
