# Supplementary material for: Targeted assembly of ectopic kinetochores to induce chromosome‐specific segmental aneuploidies
Source: EMBO J. 2023 Apr 17;42(10):e111587. doi: 10.15252/embj.2022111587 (PMC10183824; doi:10.15252/embj.2022111587)
Supplement: Supplementary file 6 [file EMBJ-42-e111587-s001.pdf]

THE EMBO JOURNAL

Targeted assembly of ectopic kinetochores to induce chromosome-specific segmental aneuploidies

Laura Tovini, Sarah Johnson, Molly Guscott, Alexander Andersen, Diana Spierings, René Wardenaar, Floris Foijer, and Sarah McClelland
DOI: 10.15252/embj.2022111587

Corresponding author(s): Sarah McClelland (S.McClelland@qmul.ac.uk)

Review Timeline:

Submission Date:	3rd May 22
Editorial Decision:	2nd Jun 22
Revision Received:	5th Feb 23
Editorial Decision:	28th Feb 23
Revision Received:	31st Mar 23
Accepted:	5th Apr 23

Editor: Hartmut Vodermaier

Transaction Report:

(Note: With the exception of the correction of typographical or spelling errors that could be a source of ambiguity, letters and reports are not edited. Depending on transfer agreements, referee reports obtained elsewhere may or may not be included in this compilation. Referee reports are anonymous unless the Referee chooses to sign their reports.)

Dr. Sarah E McClelland
Barts Cancer Institute
Centre for Cancer Genomics and Computational Biology
Queen Mary, University of London
London EC1M 6BQ
United Kingdom

2nd Jun 2022

Re: EMBOJ-2022-111587
Inducing Specific Chromosome Mis-Segregation in Human Cells

Dear Sarah,

Thank you for submitting your manuscript on generating chromosome-specific aneuploidy via ectopic kinetochores to The EMBO Journal. It has now been seen by three expert referees, in light of whose supportive comments (copied below) we shall be happy to consider this work further for publication, following adequate revisions in response to a number of constructive experimental and presentational criticisms raised in all three reports.

Since we usually aim for a single round of major revision, I would encourage you to get in contact with me already during its early stages with a tentative response/revision proposal, either in writing or by way of a brief online discussion that we might schedule. In case this should be helpful, we could also consider an extension of our default three-months revision period, during which our 'scooping protection' (meaning that competing work appearing elsewhere in the meantime will not affect our considerations of your study) would of course remain valid.

Further information on preparing and uploading a revised manuscript can be found below and in our Guide to Authors. Thank you again for the opportunity to consider this work for The EMBO Journal, and I look forward to hearing from you.

With kind regards,

Hartmut

Hartmut Vodermaier, PhD
Senior Editor, The EMBO Journal
h.vodermaier@embojournal.org

*** PLEASE NOTE: All revised manuscript are subject to initial checks for completeness and adherence to our formatting guidelines. Revisions may be returned to the authors and delayed in their editorial re-evaluation if they fail to comply to the following requirements (see also our Guide to Authors for further information):

1) Every manuscript requires a Data Availability section (even if only stating that no deposited datasets are included). Primary datasets or computer code produced in the current study have to be deposited in appropriate public repositories prior to resubmission, and reviewer access details provided in case that public access is not yet allowed. Further information: embopress.org/page/journal/14602075/authorguide#dataavailability

2) Each figure legend must specify
- size of the scale bars that are mandatory for all micrograph panels
- the statistical test used to generate error bars and P-values
- the type error bars (e.g., S.E.M., S.D.)
- the number (n) and nature (biological or technical replicate) of independent experiments underlying each data point
- Figures may not include error bars for experiments with n<3; scatter plots showing individual data points should be used instead.

3) Revised manuscript text (including main tables, and figure legends for main and EV figures) has to be submitted as editable text file (e.g., .docx format). We encourage highlighting of changes (e.g., via text color) for the referees' reference.

4) Each main and each Expanded View (EV) figure should be uploaded as individual production-quality files (preferably in .eps, .tif, .jpg formats). For suggestions on figure preparation/layout, please refer to our Figure Preparation Guidelines:

http://bit.ly/EMBOPressFigurePreparationGuideline

5) Point-by-point response letters should include the original referee comments in full together with your detailed responses to them (and to specific editor requests if applicable), and also be uploaded as editable (e.g., .docx) text files.

6) Please complete our Author Checklist, and make sure that information entered into the checklist is also reflected in the manuscript; the checklist will be available to readers as part of the Review Process File. A download link is found at the top of our Guide to Authors: embopress.org/page/journal/14602075/authorguide

7) All authors listed as (co-)corresponding need to deposit, in their respective author profiles in our submission system, a unique ORCiD identifier linked to their name. Please see our Guide to Authors for detailed instructions.

8) Please note that supplementary information at EMBO Press has been superseded by the 'Expanded View' for inclusion of additional figures, tables, movies or datasets; with up to five EV Figures being typeset and directly accessible in the HTML version of the article. For details and guidance, please refer to:
embopress.org/page/journal/14602075/authorguide#expandedview

9) Digital image enhancement is acceptable practice, as long as it accurately represents the original data and conforms to community standards. If a figure has been subjected to significant electronic manipulation, this must be clearly noted in the figure legend and/or the 'Materials and Methods' section. The editors reserve the right to request original versions of figures and the original images that were used to assemble the figure. Finally, we generally encourage uploading of numerical as well as gel/blot image source data; for details see: embopress.org/page/journal/14602075/authorguide#sourcedata

We realize that it is difficult to revise to a specific deadline. In the interest of protecting the conceptual advance provided by the work, we recommend a revision within 3 months (31st Aug 2022). Please discuss the revision progress ahead of this time with the editor if you require more time to complete the revisions. Use the link below to submit your revision:

Link Not Available

Referee #1:

In this manuscript, Tovini and Johnson et al. present a highly innovative tool to induce the mis-segregation of specific chromosomes in human cells. Their approach utilizes nuclease-dead Cas9 to achieve localization to specific genomic regions and, in conjunction with tethered CENP-T, form ectopic kinetochores. Further, to achieve mis-segregation the authors wash-in an MPS1 inhibitor to facilitate spindle assembly checkpoint satisfaction thus anaphase onset and ultimately chromosome mis-segregation. Indeed, the authors demonstrate specific mis-segregation of chromosomes 1 and 9. At this point, the authors' efforts to mis-segregate chromosome 3 and showing "negative" data deserve commendation.

Overall, this is a very important piece of work relevant to the field of aneuploidy and chromosomal instability where modeling individual chromosome missegregation is an important yet to date unattained goal. The authors characterize their system robustly from a cell biological point of view, however, questions about the efficiency of their system to specifically mis-segregate chromosomes with the ultimate aim "to create designer karyotypes" might perhaps need further exploration. We are highly supportive of publication of this work and have listed some constructive comments below:

Major points:
1. To mis-segregate chromosomes an MPS1 inhibitor treatment, albeit brief, is required to satisfy the spindle assembly checkpoint. This approach leads to an increase of mis-segregation events on its own yet, it seems that all mis-segregating chromosomes are EGFP+ as shown in Fig. 4E. This seems to be a HEK293T effect as it is not the case in HCT116 cells shown in Fig. 4F. It would be helpful to explore this effect a little further. Do difference cell lines require different reversine concentrations? Some additional characterization of the effect of reversine treatment (at different concentrations) on various cell lines would go a long way in ensuring wide applicability of this method.
2. There is some discrepancy between single-cell DNA sequencing data and the mis-segregation data in Fig. 4. The sequence data largely shows the generation of sub-chromosomal copy number changes. This is also indicated by the analysis which focuses of CNAs larger than 20Mb. Given the previous point regarding the difference between HEK293T and HCT116 cells, does this same observation hold true in HCT116 cells? It seems that the HCT116 data might be more robust as the authors did not observe as many EGFP+ mis-segregation events in the absence of CENP-T. Could this discrepancy be explained by the way the analysis was performed? How would this data look if an aneuploidy or heterogeneity score was calculated per chromosome as was done by Ippolito et al. (2021, Dev Cell)?
3. In line with the previous point, do the authors observe specific focal CNAs in the regions where dCas9 is targeted to?
4. What is the fate of the mis-segregated chromosomes? Lagging chromosomes are known to be encapsulated in micronuclei at

varying rates which could be a limitation of this method if the mis-segregated chromosome undergoes additional genomic changes (mainly chromothripsis) other than just generating aneuploidy.
5. It would be helpful to expand on the additional benefit gained from this compared to prior methods which use CRISPR based techniques to induce chromosome arm losses (ie Leibowitz, M.L., Papathanasiou, S., Doerfler, P.A. et al. Chromothripsis as an on-target consequence of CRISPR-Cas9 genome editing. Nat Genet 53, 895-905 (2021)

Minor points:
1. Please, double-check the spelling of the DNA dye Hoechst in Fig. 3E.
2. Please check the resolution of figures, for instance the text in Figure 3
3. Figure 4F why is the missegregation of EGFP- chromosomes reduced in CENP-TdeltaC-dCAS9-EGFP cells compared to the dCAS9-EGFP controls? One would expect them to be the same?
4. Figure S3A, please label the chromosomes.

Referee #2:

Reviewer overview:
This is an interesting resource article by Tovini, Johnson, and colleagues that presents an innovative approach to induce chromosome-specific mis-segregations in human cells. To induce chromosome-specific mis-segregation the authors came up with an attractive idea to generate pseudo-dicentric chromosomes by nucleating ectopic kinetochores on specific genomic loci of certain chromosomes. Such ectopic kinetochores would be presumably prone to erroneous attachments of kinetochores to microtubules leading to unbalanced chromosome segregation. Authors first successfully mediated dCas9 recruitment of CENP-T C to an ectopic site on chromosome 3, but interestingly this approach was not able to recruit downstream kinetochore component KNL-1, and thus functional kinetochore that could bind microtubules. Therefore, authors recruited CENP-T C-dCas9 to a larger chromosome locus including pericentromeric region of chromosome 9, and subtelomeric region of chromosome 1, where they predicted much larger numbers of sgRNA binding sites. This approach yielded much higher overall intensities of CENP-T on ectopic centromeres, and successfully recruited large amounts of KNL-1 and Ndc80, thus enabling recruitment of microtubules to functional ectopic kinetochores. The authors also followed ectopic kinetochores by live-cell imaging but observed substantial mitotic delays in cells with ectopic kinetochores, a phenotype that was relieved by inhibition of Mps1 kinase, and to a smaller degree by inhibition of Aurora B kinase. To study consequences of ectopic kinetochore strategy they inhibited Mps1 in cells with ectopic kinetochores and observed increase in number of mis-segregations of targeted chromosomes. Finally, the authors used single cell sequencing approach to show that chromosome specific aneuploidies are observed in cells with ectopic kinetochores on particular chromosome, especially clear for chromosome 9, that further led to an increase in the proportion of chromosome breaks close to the region where ectopic kinetochore was localized on the chromosome.

I think this is a quality study that will be of interest to researchers working on mechanisms of kinetochore and centromere formation and function, causes and consequences of chromosome mis-segregation, aneuploidy and chromosomal instability. Prior studies that attempted to resolve immediate effects of chromosome mis-segregation have been hampered by the complexity of the experimental systems used to induce chromosome mis-segregation. Currently, inhibitors of SAC are most common strategy used to induce chromosome mis-segregations and study immediate consequences of aneuploidy. However, it has proven difficult to experimentally control for both chromosome identity and the degree of chromosome mis-segregation that one induces in cells by using such approaches, and both have been shown to be important for the cellular response to aneuploidy. In addition, many groundbreaking approaches have relied on different cell lines that have already adapted to some specific aneuploidy, which hinders the study of immediate effects of aneuploidy. In that regard, I congratulate the authors on this creative idea that I see as an important step towards end goal of inducing chromosome-specific mis-segregations in human cells. However, the presented approach is limited to aneuploidies of chromosome arms presumably through chromosome breakage events that are known to induce highly different cellular response when compared to whole chromosome aneuploidies (Santaguida et al., 2017, Dev Cell; Hintzen et al., 2021, bioRxiv, Soto et al., 2017, Cell Rep). Furthermore, currently presented approach is limited by the usage of Mps1 inhibitor to induce mis-segregation that has some clear limitations that I mentioned above, especially since one of the goals of the presented work is to replace such experimental approaches with a more suitable one.

The manuscript is well-written, and Results, Materials and methods parts are clean and clear for the most part except for a few instances emphasized in the comments below. Although I think that the manuscript presents interesting approach that could allow for more detailed follow-up studies on cellular consequences of enriching aneuploidies associated with the specific arm of certain chromosomes, authors should revise manuscript to better reflect certain results, as per comments below, and introduce one paragraph explaining limitation of the presented approach within the Discussion. Furthermore, I think that authors should discuss more implications of presented results, and analyze movies from live-cell imaging approach that for some unknown reason were not analyzed in the presented manuscript. If authors would succeed in presenting the stronger view of main implications and limitations of the approach, my opinion is that the manuscript would be at the EMBO Journal level.

Major comments:
1) In the Introduction part of the manuscript (page 2, line 63) the authors state that the presented approach could be fine-tuned

to allow for 'provoked mis-segregation of any specific chromosome, in any given cell line...'. However, authors should discuss more on prevalence of endogenous repetitive arrays on human chromosomes and if every chromosome is equally appropriate for this approach as authors themselves show that an endogenous repetitive array must be of certain minimal size that is characterized by minimal size of sgRNA binding sites. Furthermore, results on HEK cells are much less convincing then results on HCT cells, and results on chromosome 9 in HEK cells are much cleaner than those on chromosome 1 (see comments below). Authors should discuss this more, and incorporate possible limitations of the study in a separate paragraph of the Discussion section.
2) I completely missed the reason why the authors did not score mis-segregations from live-cell movies, as those could be seen both in presented movie and in Figure 3E. Authors could exclude the cells that did not enter anaphase during the imaging time and score only cells that entered anaphase. Alternatively, the authors should present clear reasons why they omitted this analysis.
3) Data presented on Figure 4E is really puzzling to me. First, overall chromosome mis-segregation rate is lower in Mps1-treated then in untreated cells. I do not know of any paper that reported similar phenotype, and this should be discussed. Furthermore, the authors should comment on the observed base difference between mis-segregation rates of chromosome 9 and chromosome 1 in HEK cells upon Mps1 inhibition. Chromosome 9 seems to mis-segregate much more often than chromosome 1 in the case of dCas9-EGFP Mps1-treated cells, but they mis-segregate to a similar degree in CENP-T C-dCas9-EGFP Mps1-treated cells. The observation that chromosome 9 mis-segregates more in HEK dCas9-EGFP cells treated with Mps1 is at odds with the similar data presented on Figure 5 where frequency of non-clonal CNAs per cell is much more prominent for chromosome 1 then for chromosome 9 in dCas9 and no guide conditions. Also, it is puzzling how Mps1 inhibition did not increased mis-segregations of EGP- chromosomes after Mps1 inhibition in HEK cells, because it expectedly managed to do so in HCT cells. Authors should discuss these issues.
4) The authors should discuss more the puzzling data presented for Chromosome 1 on Figure 5E, and implications of such observations for the usage of Cas9 approaches in chromosome-specific labelling. If a understood the graph correctly, higher aneuploidy score for chromosome 1 is observed even when dCas9-EGFP is targeted to chromosome 9, and to a rather similar degree when dCas9-EGFP is targeted to the chromosome 1, or when CENP-T C-dCas9-EGFP is targeted on either chromosome 9 or chromosome 1. The possible reasons and implications should be discussed. Also, the authors should cite the work that showed how incomplete replication of the chromosomal repeats could be caused by binding of Cas9 to those repeats during S phase (Whinn et al., 2019, Sci Rep).

Minor comments:
1) It would be interesting if authors could measure interkinetochore distances as a proxy for microtubule attachment and tension across ectopic kinetochores in different conditions including dCas9-EGFP, CENP-T C-dCas9-EGFP, with and without nocodazole, at least in situations where this is possible due to the bar-like and rather fragmented shape of ectopic kinetochores.
2) As it was shown that SAC response scales with number of unattached kinetochores (Dick&Gerlich, 2013, Nat Cell Biol), it would be interesting for authors to check if substantial mitotic delays are due to the presence of unattached kinetochores by performing Mad labelling of cells with ectopic kinetochores. The authors said in the Discussion part that they '...did not notice the presence of obviously unattached kinetochores herein' but without presenting any data for this claim. Authors should in any way discuss more the possible reasons why would ectopic kinetochores induce substantial mitotic delays, as it is not obvious that every improper attachment would result in SAC activation, as authors state.
3) The conclusion presented on page 9, line 212, is a bit strange as Aurora B inhibition is known to affect SAC response per se, as the authors nicely point out in the Discussion part of the manuscript (see also Gurden et al., 2018, Oncotarget; Santaguida et al., 2011, EMBO J). I would avoid such unclear conclusions in the Results section.
4) On page 10, line 222, the authors classified ectopic kinetochore mis-segregation as any event where lagging chromosome appeared in the center of the segregating DNA masses. However, due to usage of fixed cell analysis, authors cannot tell if such lagging structures would resolve later during anaphase, as shown nicely by a series of recent publications implicating Aurora B in error correction of lagging chromosomes during early anaphase (Orr et al., 2021, Curr Biol; Sen et al., 2021, Dev Cell).
5) The authors should calculate and comment on statistical significance between data presented on Figures 5C and 5E.
6) One of the main predications of the study is that generation of ectopic kinetochores would induce erroneous kinetochore microtubule attachments, but the authors do not present any evidence that attachments on ectopic kinetochores are indeed erroneous during mitosis. Authors should at least discuss why would such kinetochores be more prone to formation of erroneous attachments, and what types of attachments they expect.
7) Authors should acknowledge that whole chromosome aneuploidies are rarely observed in this study, and emphasize more the limits of Mps1 inhibitor usage in the Discussion part of the manuscript.
8) I would suggest the authors to change the manuscript title to one that is more specific, reflecting the exact approach that was used.

Additional comments:
9) On Figure 1C in the dCas9-GFP subpanel I see 4 dots of EGFP signal in the inset, but in CENP-T C I see only two dots, and in EGFP panel I can see multiple CENP-T signals of varying intensities. Could authors explain better the number of dots of CENP-T we should expect to see?
10) On page 7, line 166, the authors say that 'Such fibers are rarely observed in EGFP-negative...', but do not give any data on this claim. Authors should present IF data on this or delete this sentence.
11) In the caption for Figure 4E and F authors state that 'red line = mean error rate in untreated cells' but it is unclear to me from where they acquired these numbers, as they are not presented in the manuscript.

12) On page 10, line 230, the authors state that: 'This was true for both chromosome 9 and 1, and in both HEK293T and HCT116 cells'. However, chromosome 1 data in HEK cells is not significant when comparing dCas9-EGFP and CENP-T C-dCas9-EGFP MPS1-treated cells. Authors should also discuss more the possible reasons for differences observed between HEK and HCT cells.
13) Are the microscopy images presented in figures maximal projections or a single z-planes?
14) I recommend that the authors label the q and p arms, and gains and losses on the CAN pileups to ease their reading.
15) Color shades presented in CAN pileups are explained only in Figure S3, and not in Figure 5.
16) The authors should speculate more on reasons why ectopic kinetochores do not assemble on ectopic site when smallest array of MUC4 locus on chromosome 3 was used, as it is currently unclear.

Referee #3:

Comments to the authors

Recurrent aneuploidy patterns have been observed in various cancer types. The origin of these aneuploidy patterns and how do they evolve remains unknown. The challenge has been the lack of a strategy to induce specific chromosome aneuploidies in specific cell types so that the immediate cellular responses can be evaluated and eventually the later adaptive responses during clonal expansion examined. The approaches available so far are limited because either rely on clonal expansion upon an initial karyotype change or generate karyotype changes randomly. In this study, Tovoni et al. describe a novel strategy to mis-segregate specific chromosomes in different human cell lines. The strategy consists on targeting the kinetochore-nucleating domain of centromere protein CENP-T to assemble ectopic kinetochores in chromosome-specific endogenous repeats (chromosomes 1, 3 and 9). Ectopic kinetochores in chromosomes 1 and 9 induced elevated mis-segregation and aneuploidy on the targeted chromosomes. The strategy still faces limitations but provides an exciting basis for further improvement. The mitotic checkpoint activation by the ectopic kinetochores implies the use of checkpoint inhibitors which induce mis-segregation of non-targeted chromosomes. Moreover, the strategy primarily induces segmental vs. whole chromosomal aneuploidies, which might lead to different cellular responses. Nevertheless, an important step towards the generation of specific aneuploidies was given, using elegant challenging methodologies. I am supportive of the publication provided the authors address the concerns below.

Major comments

1. The title should perhaps be more specific, e.g., 'Targeted assembly of ectopic kinetochores to induce chromosome-specific segmental aneuploidies in human cells'.

2. I reviewed the related manuscript by Truong et al., also submitted to this journal, in which a motor-based approach is used to induce chromosome-specific mis-segregations. I consider that both manuscripts, while under a common goal and similar experimental approaches, provide complementary data.

2.1. Here, Tovoni et al. provide new insights into ectopic kinetochore biology, namely on the size and position of the target array required for ectopic kinetochore formation. Details of guide RNAs used to target 3 chromosomes and their binding sites are adequately described in Table 1. An approximate threshold of >1441 guide RNA binding sites required to assemble CENP-T-nucleated ectopic KTs could be inferred. It would be useful if the authors could extend their analysis into other chromosomes exhibiting targetable endogenous repeats (localization and size) to infer the extrapolation of the strategy to other chromosomes.

2.2. CENP-TdC-nucleated KTs were shown to induce the mitotic spindle assembly checkpoint - mitotic delay via Aurora B activity. Could the authors include (similarly to Truong et al.) any immunostaining for a checkpoint protein?

2.3. The mitotic delay induced by CENP-TdC-nucleated KTs is disadvantageous in comparison to the motor-based approach described by Truong et al. since it compels the use of checkpoint inhibitors to induce chromosome mis-segregation. The authors circumvented the mitotic arrest by using pulses of Mps1i and AurkBi. Whereas mis-segregation of non-EGFP (non-targeted) chromosomes was not apparent in HEK293T cells (but these cells have high error rate in untreated controls, Fig.4E), it was significant in HCT116 cells despite their lower error rate (Fig.4F). Still, dCas9-EGFP-CENP-TdC induced mis-segregation of EGFP+ chromosomes much more than non-EGFP chromosomes and vs. dCas9-EGFP control.

2.3.1. Did the authors do a similar analysis to Fig. 4F but using the AurkBi (that worked efficiently in HCT116 cells, Fig. 4B)? Perhaps AurkBi would induce less mis-segregation events in non-EGFP chromosomes.

2.3.2. Why did the authors use HEK293T Mps1i for single-cell sequencing if the HCT116 Mps1i (or AurkBi) appear to exhibit better EGFP+ mis-segregation (induction of Chr1 mis-segregation is not striking in HEK293T cells (Fig. 4E), further complicated by the fact that Chr1 exhibited a high frequency of aneuploidies across most conditions, Fig.5A,E)?

2.4. Here, Tovoni et al. provide deeper analysis of breakpoints enrichment in chromosomes 1 and 9 in comparison to Truong et al. (elegant Figs.4B,D), although Truong et al. motor-based strategy appeared to work more efficiently in the induction of mis-

segregation events (perhaps because RPE1 cells were used in single-cell sequencing analysis). This shows that the position of target array can dictate the arm-level aneuploidy generation. It is interesting that ectopic kinetochore assembly worked better in Chr9-cen vs. Chr1-tel (Tovoni et al.), whereas motor-based approach worked better in Chr1-tel vs. Chr9-cen (Truong et al.) (dCas9-GFP control also induced Chr9 mis-segregation in this study).

3. The authors should tone down all over the manuscript that the strategy tested induced segmental (not whole) chromosome-specific aneuploidies.

4. The authors mention in the discussion, the generation of stable cell lines with the capacity to create targeted ectopic KTs at any locus. But as described in Truong et al. clonal expansion to derive the dCas9-Kin14VIb cell line ended up generating an X trisomy. This somehow limits the efficacy of the strategy to study immediate effects of induced chromosome-specific aneuploidies. How do the authors envision to circumvent this?

5. Since the strategy described ends up requiring the use of Mps1i, the authors should argue about the advantage of dCas9-EGFP-CENP-TdC targeting + Mps1i pulse to study the immediate consequences of specific chromosome aneuploidies vs. a strategy using dCas9-EGFP (only for specific chromosome visualization) + Mps1i (Mps1i has been used to generate karyotype changes randomly that could be tagged if using dCas9-EGFP targeting to chromosome-specific repeats). Would this facilitate the induction of whole vs. segmental specific aneuplodies?

Response to reviewers for “Inducing specific chromosome mis-segregation in human cells”

(new title: “Targeted assembly of ectopic kinetochores to induce chromosome-specific segmental aneuploidies”)

Overview of major changes:

We greatly thank all three reviewers for their supportive comments on our manuscript, and their constructive and helpful suggestions to improve our manuscript. We have addressed all queries in full. Text edits in the manuscript are in red text, and the main alterations are as follows:

1. Amendment of the title to better reflect our findings.
2. Reanalysed and presented targeted chromosome segregation error rates, background Mps1i error rates, and new experiments titrating Mps1i concentrations to attempt to reduce background error rates.
3. Reanalysed single cell sequencing data and specific analysis of focal CNAs.
4. Tracking the fate of mis-segregated/lagging target chromosomes into daughter cells or micronuclei using live imaging.
5. Analysis of additional suitable repetitive target sites in the T2T reference genome revealing the putative ability to target at least 18 of the 22 autosomes.
6. Incorporation of additional discussion sections addressing limitations and advantages of our approach over prior approaches, and clarification throughout the manuscript of the impact of our approach on the target chromosomes (segmental vs. whole aneuploidies).

Response to Reviewer 1

In this manuscript, Tovini and Johnson et al. present a highly innovative tool to induce the mis-segregation of specific chromosomes in human cells. Their approach utilizes nuclease-dead Cas9 to achieve localization to specific genomic regions and, in conjunction with tethered CENP-T, form ectopic kinetochores. Further, to achieve mis-segregation the authors wash-in an MPS1 inhibitor to facilitate spindle assembly checkpoint satisfaction thus anaphase onset and ultimately chromosome mis-segregation. Indeed, the authors demonstrate specific mis-segregation of chromosomes 1 and 9. At this point, the authors' efforts to mis-segregate chromosome 3 and showing "negative" data deserve commendation. Overall, this is a very important piece of work relevant to the field of aneuploidy and chromosomal instability where modeling individual chromosome missegregation is an important yet to date unattained goal. The authors characterize their system robustly from a cell biological point of view, however, questions about the efficiency of their system to specifically mis-segregate chromosomes with the ultimate aim "to create designer karyotypes" might perhaps need further exploration. We are highly supportive of publication of this work and have listed some constructive comments below.
> We thank the reviewer for their careful assessment of our manuscript and their overall positive response and greatly appreciate the constructive comments which we have addressed in full below.

Major points:
1. To mis-segregate chromosomes an MPS1 inhibitor treatment, albeit brief, is required to satisfy the spindle assembly checkpoint. This approach leads to an increase of mis-segregation events on its own yet, it seems that all mis-segregating chromosomes are EGFP+ as shown in Fig. 4E. This seems to be a HEK293T effect as it is not the case in HCT116 cells shown in Fig. 4F. It would be helpful to explore this effect a little further.
> We apologise for any misunderstanding due to our original format of Figure 4e (any cell with an EGFP+ chromosome could also have had a co-mis-segregating EGFP-negative chromosome but this was not

clear from our original presentation). We have now re-presented this data (**new Figure 5e,f, also shown below**) to indicate cells with (i) EGFP-negative only, (ii) EGFP+ only or (iii) both EGFP+ and EGFP-negative chromosomes mis-segregating. We have also performed additional repeats of these experiments to increase cell numbers and improve the robustness of this analysis, since we noticed that one experiment was an outlier that contributed to the confusing original data. With increased cell numbers analysed, and this clearer presentation we hope it is now more evident that indeed Mps1 inhibition *per se* elevates segregation errors both in the untransfected conditions (first two bars) and the dCas9 conditions.

Figure 5e and f:

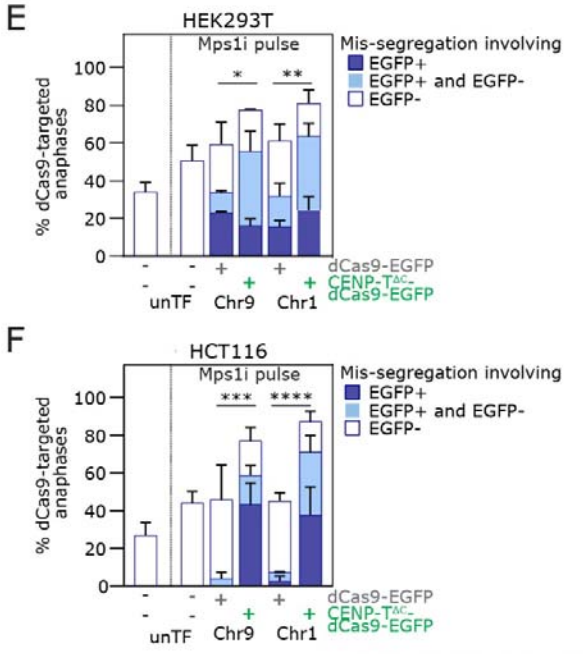

Do difference cell lines require different reversine concentrations? Some additional characterization of the effect of reversine treatment (at different concentrations) on various cell lines would go a long way in ensuring wide applicability of this method.

> We previously showed that RPE1 cells (non-transformed retinal pigment epithelial cells) required only 250 nM Reversine to induce 50% segregation errors (Worrall et al, Cell Reports 2018) suggesting that indeed different cell types may require fine-tuning to obtain optimal conditions. We think testing different reversine concentrations is a very good suggestion since it has been previously shown that titrating down Mps1 inhibitor concentrations can lower mis-segregation rates in RPE1 and HCT116 cells (Sansregret et al, Cancer Discovery, 2017). To test this whether we could see a similar effect, we assessed chromosome segregation error rates in both HEK293T and HCT116 cells upon three different concentrations of Mps1i (new **Figure EV2f-g, shown below**). We observed a mild reduction in the chromosome segregation error rate with lower Mps1i concentrations in HCT116 cells (new **Figure EV2g)**.

The reduction was not apparent in the HEK293 cells however it is possible that in ectopic kinetochore conditions this lower Mps1i concentration could reduce background error rates. Therefore, we also tested whether these lower Mps1i concentrations were also able to release a mitotic arrest caused by ectopic kinetochore formation, which would be critical in our intended use. In HEK293 cells these lower concentrations were able to partially release metaphase arrest caused by ectopic kinetochore formation, however at a reduced rate of efficiency (new **Figure EV2h, also shown below**).

Figure EV2f-h:

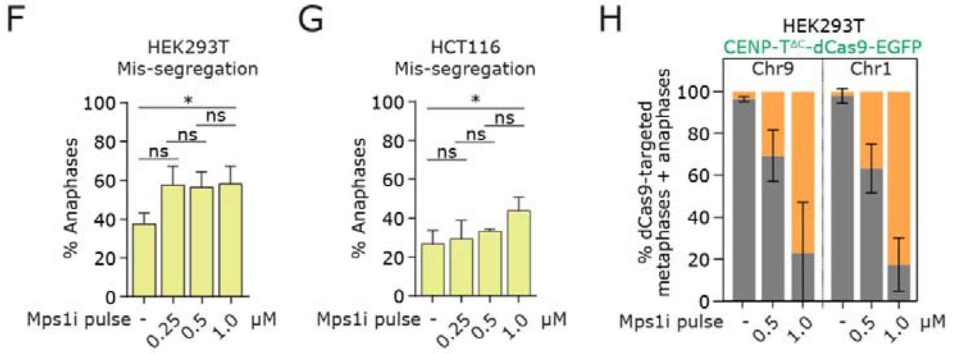

Given the overall relatively small increase in error rates caused by our original Mps1i concentration (~15% increase), and the lower efficiency of mitotic arrest release upon titrating Mps1i down, we conclude that in these cell lines 1µM Mps1i is probably the most effective concentration but that this should be empirically determined in other labs and cell lines. This is now included in the discussion:

*"To overcome the mitotic arrest we took the approach of bypassing arrest using Mps1 inhibition. Herein we used 1 µM, a dose that efficiently released arrest in HEK293 and HCT116 cells. This was the optimal dose balancing efficient release of arrest with lowest possible error rate. However lower doses of Mps1i may be enough to release arrest in other cell types which may reduce background error rates (**Figure EV2g**) so this should be determined in each cell line."*

2. There is some discrepancy between single-cell DNA sequencing data and the mis-segregation data in Fig. 4. The sequence data largely shows the generation of sub-chromosomal copy number changes. This is also indicated by the analysis which focuses of CNAs larger than 20Mb. Given the previous point regarding the difference between HEK293T and HCT116 cells, does this same observation hold true in HCT116 cells? It seems that the HCT116 data might be more robust as the authors did not observe as many EGFP+ mis-segregation events in the absence of CENP-T. Could this discrepancy be explained by the way the analysis was performed? How would this data look if an aneuploidy or heterogeneity score was calculated per chromosome as was done by Ippolito et al. (2021, Dev Cell)?

> We think the reviewer refers to a potential discrepancy between the observation of chromosome mis-segregation events at anaphase, and the ultimate manifestation of aneuploidy as sub-chromosomal alterations, whereas mis-segregation at anaphase could be expected to result in whole chromosomal aneuploidies in daughter cells. We thank the reviewer for pointing out this potential confusion. We think there is not likely to be a discrepancy here for two reasons; first, the microscopy images cannot easily distinguish between a whole, or a partial chromosomal material lagging at anaphase. Secondly, whole chromosome laggards may themselves undergo subsequent damage and breakage during anaphase which would also lead to segmental aneuploidies in daughter cells. We hope this clarifies the reviewer's concern and have made these points clearer in the manuscript when describing the analysis of chromosome mis-segregation events:

"Note that given the resolution of the microscopy we cannot determine whether mis-segregating chromatin was composed of whole intact chromosomes, or partial and/or damaged chromosomes."

And:

"The observation of mainly sub-chromosomal aneuploidies from the sequencing data suggests that either only partial chromosomes are observed mis-segregating in Figure 5, or that lagging chromosomes are subject to damage during cell division that results in partial chromosome copy number changes in daughter cells."

We agree that analysis of SCS from HCT116 cells would have been informative, however this was unfortunately not experimentally feasible due to the lower transfection efficiency in this line which would have required an extremely costly analysis of much larger numbers of cells to be sequenced. As the method progresses we expect to be able to perform more extensive single cell sequencing analysis of downstream aneuploidy events caused by ectopic kinetochore formation, and we agree it would be interesting to examine whether there could be cell type specific differences in the conversion of induced chromosome mis-segregation events to aneuploidy.

Regarding the analysis of the single cell sequencing data, this was challenging against the background heterogeneity of the HEK293 cell line. We did (in collaboration with the Foijer lab) perform a similar heterogeneity analysis per chromosome, or chromosome arm, as Ippolito et al (please see below), however it was difficult to see a clear difference for the target chromosomes because of the background heterogeneity and the low incidence rate, therefore we did not include this in the original submission.

Heterogeneity analysis:

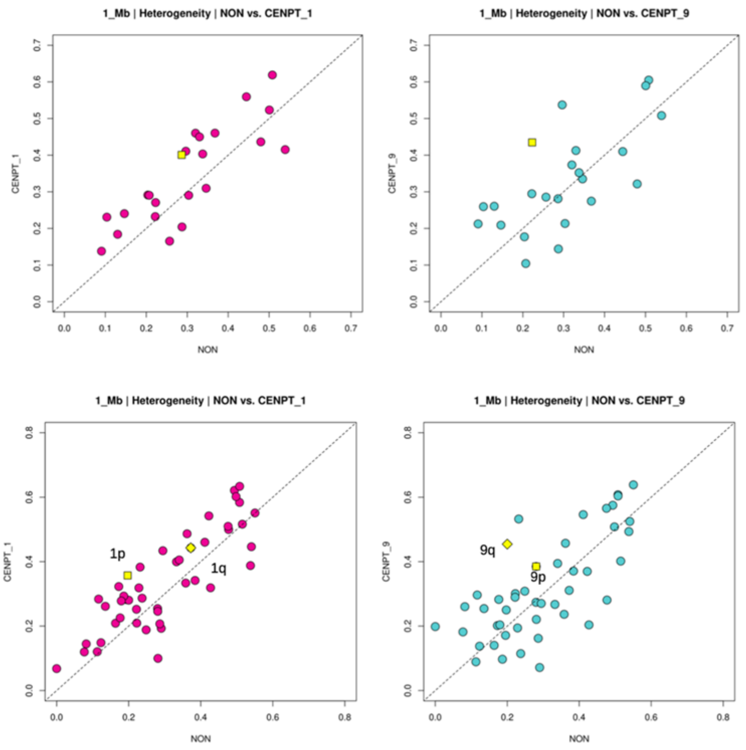

We have performed a minor reanalysis of the single cell sequencing data to improve the removal of background subclonal CNAs which we hope helps the clarity of this data (now **Figure 6**).

3. In line with the previous point, do the authors observe specific focal CNAs in the regions where dCas9 is targeted to?

> We have now analysed the SCS data specifically to look for breakpoints in the vicinity of the dCas9 target sites but we do not observe any (**now Figure 6e-g, also shown below**). It will be interesting however to track the longer term fate and genomic alterations in cells that suffered ectopic kinetochore-induced mis-segregation which we plan to do as part of future studies.

Figure 6e-g:

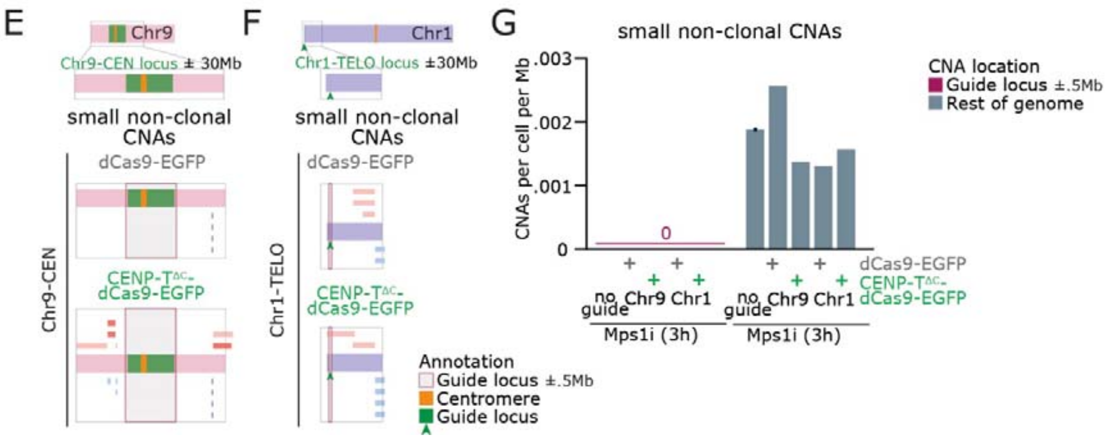

4. What is the fate of the mis-segregated chromosomes? Lagging chromosomes are known to be encapsulated in micronuclei at varying rates which could be a limitation of this method if the mis-segregated chromosome undergoes additional genomic changes (mainly chromothripsis) other than just generating aneuploidy.

> We agree this is an important question, and have now assessed this using live cell microscopy to follow the fate of the mis-segregated target chromosomes. We imaged H2B-RFP-labelled HEK293T cells for 4 hours following Mps1 inhibitor addition. For both chromosomal targets we observed only low rates of incorporation into MN (<5%). Instead, the most common fate was an unresolved bridge that is also likely to undergo subsequent processing and/or breakage. This new data is included in Figure 5g-i (formerly Figure 4) shown below. We also include a discussion of this point:

Lastly, although only a low rate of micronucleus formation was observed, the majority of mis-segregating target chromosomes led to chromatin bridges that persisted for several hours. Further study is therefore required to determine their ultimate fate, however we anticipate this could be a useful model to study the breakage of chromatin bridges, a common feature in cancer cells.

Figure 5g-i:

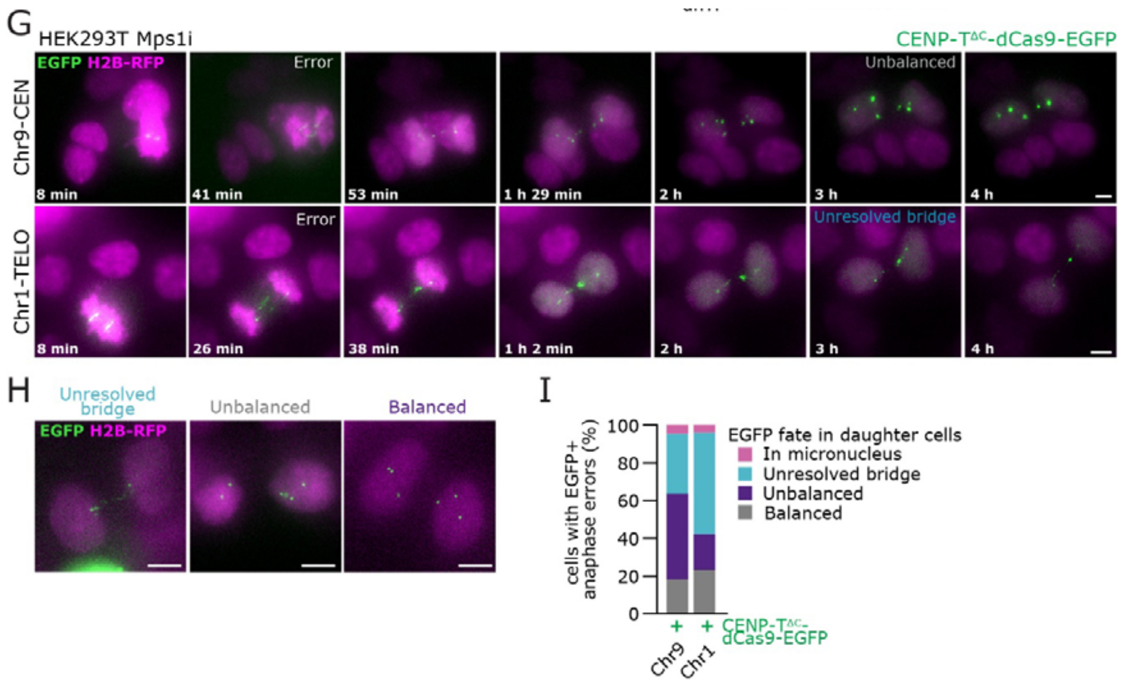

5. It would be helpful to expand on the additional benefit gained from this compared to prior methods which use CRISPR based techniques to induce chromosome arm losses (ie Leibowitz, M.L., Papathanasiou, S., Doerfler, P.A. et al. Chromothripsis as an on-target consequence of CRISPR-Cas9 genome editing. Nat Genet 53, 895-905 (2021).

> We appreciate the reviewer's suggestion and have included an additional section in the Discussion: "Benefits of our approach over prior systems":

*"Here we set out to mimic specific chromosome mis-segregation events and the resulting aneuploidies. We were able to efficiently assemble ectopic kinetochores and induce specific chromosome mis-segregation in two cell lines. Compared to CRISPR-cutting based approaches our ectopic kinetochore-mediated mis-segregation rate was high (55-75% transfected cells were induced to mis-segregate the target chromosome (**Figure 5c-f**), compared to 7.5% for MN generation by CRISPR cutting[1,2]. Secondly, our approach might be considered a better model of cancer chromosome mis-segregation, by mimicking the formation of dicentric chromosomes[3,4], and faulty kinetochore-microtubule attachments[5,6]. Furthermore, our method provides a tuneable system to further investigate kinetochore assembly regulation, to complement previous studies[7-9] for example allowing investigation of the impact of assembling ectopic kinetochores at differing locations or chromosomal contexts."*

Minor points:

Please, double-check the spelling of the DNA dye Hoechst in Fig. 3E

> We have now corrected the misspelling of labeling in Figure 3e.

Please check the resolution of figures, for instance the text in Figure 3

> Thank you, this has now been corrected.

Figure 4F why is the missegregation of EGFP- chromosomes reduced in CENP-TdeltaC-dCAS9-EGFP cells compared to the dCAS9-EGFP controls? One would expect them to be the same?
> We apologize for the lack of clarity of this figure. We have now better organized the data in Figure 4F (now **Figure 5f**), and separated errors involving only EGFP+ chromatids vs those where EGFP- chromatids also mis-segregate alongside EGFP+ for clarity.

Figure S3A, please label the chromosomes.
> Figure EV4A now includes chromosome labels, we apologize for missing this in the first version of the manuscript.

Response to Reviewer 2

This is an interesting resource article by Tovini, Johnson, and colleagues that presents an innovative approach to induce chromosome-specific mis-segregations in human cells. To induce chromosome-specific mis-segregation the authors came up with an attractive idea to generate pseudo-dicentric chromosomes by nucleating ectopic kinetochores on specific genomic loci of certain chromosomes. Such ectopic kinetochores would be presumably prone to erroneous attachments of kinetochores to microtubules leading to unbalanced chromosome segregation. Authors first successfully mediated dCas9 recruitment of CENP-TDC to an ectopic site on chromosome 3, but interestingly this approach was not able to recruit downstream kinetochore component KNL-1, and thus functional kinetochore that could bind microtubules. Therefore, authors recruited CENP-TDC-dCas9 to a larger chromosome locus including pericentromeric region of chromosome 9, and subtelomeric region of chromosome 1, where they predicted much larger numbers of sgRNA binding sites. This approach yielded much higher overall intensities of CENP-T on ectopic centromeres, and successfully recruited large amounts of KNL-1 and Ndc80, thus enabling recruitment of microtubules to functional ectopic kinetochores. The authors also followed ectopic kinetochores by live-cell imaging but observed substantial mitotic delays in cells with ectopic kinetochores, a phenotype that was relieved by inhibition of Mps1 kinase, and to a smaller degree by inhibition of Aurora B kinase. To study consequences of ectopic kinetochore strategy they inhibited Mps1 in cells with ectopic kinetochores and observed increase in number of mis-segregations of targeted chromosomes. Finally, the authors used single cell sequencing approach to show that chromosome specific aneuploidies are observed in cells with ectopic kinetochores on particular chromosome, especially clear for chromosome 9, that further led to an increase in the proportion of chromosome breaks close to the region where ectopic kinetochore was localized on the chromosome.
I think this is a quality study that will be of interest to researchers working on mechanisms of kinetochore and centromere formation and function, causes and consequences of chromosome mis-segregation, aneuploidy and chromosomal instability. Prior studies that attempted to resolve immediate effects of chromosome mis-segregation have been hampered by the complexity of the experimental systems used to induce chromosome mis-segregation. Currently, inhibitors of SAC are most common strategy used to induce chromosome mis-segregations and study immediate consequences of aneuploidy. However, it has proven difficult to experimentally control for both chromosome identity and the degree of chromosome mis-segregation that one induces in cells by using such approaches, and both have been shown to be important for the cellular response to aneuploidy. In addition, many groundbreaking approaches have relied on different cell lines that have already adapted to some specific aneuploidy, which hinders the study of immediate effects of aneuploidy. In that regard, I congratulate the authors on this creative idea that I see as an important step towards end goal of inducing chromosome-specific mis-segregations in human cells. However, the presented approach is limited to aneuploidies of chromosome arms presumably through chromosome breakage events that are known to induce highly different cellular response when compared to whole chromosome aneuploidies (Santaguida et al., 2017, Dev Cell; Hintzen et al., 2021, bioRxiv, Soto et al., 2017, Cell Rep). Furthermore, currently presented approach is limited by the usage of Mps1 inhibitor to induce mis-

segregation that has some clear limitations that I mentioned above, especially since one of the goals of the presented work is to replace such experimental approaches with a more suitable one.

The manuscript is well-written, and Results, Materials and methods parts are clean and clear for the most part except for a few instances emphasized in the comments below. Although I think that the manuscript presents interesting approach that could allow for more detailed follow-up studies on cellular consequences of enriching aneuploidies associated with the specific arm of certain chromosomes, authors should revise manuscript to better reflect certain results, as per comments below, and introduce one paragraph explaining limitation of the presented approach within the Discussion. Furthermore, I think that authors should discuss more implications of presented results, and analyze movies from live-cell imaging approach that for some unknown reason were not analyzed in the presented manuscript. If authors would succeed in presenting the stronger view of main implications and limitations of the approach, my opinion is that the manuscript would be at the EMBO Journal level.
> We are pleased that the reviewer recognized the quality of the study and its potential interest to researchers working on mechanisms of kinetochore and centromere formation and function, causes and consequences of chromosome mis-segregation, aneuploidy and chromosomal instability. We also agree on the need to expand the Discussion section with the limitations of the presented approach. The new manuscript provides an updated Discussion section entitled “Benefits and limitations of the current approach”.

Major comments:
1) In the Introduction part of the manuscript (page 2, line 63) the authors state that the presented approach could be fine-tuned to allow for 'provoked mis-segregation of any specific chromosome, in any given cell line...'. However, authors should discuss more on prevalence of endogenous repetitive arrays on human chromosomes and if every chromosome is equally appropriate for this approach as authors themselves show that an endogenous repetitive array must be of certain minimal size that is characterized by minimal size of sgRNA binding sites. Furthermore, results on HEK cells are much less convincing then results on HCT cells, and results on chromosome 9 in HEK cells are much cleaner than those on chromosome 1 (see comments below). Authors should discuss this more, and incorporate possible limitations of the study in a separate paragraph of the Discussion section.
> We agree with the reviewer on the lack of discussion regarding how our approach could be extended to other chromosomes. The recent release of the T2T assembly[10] allowed us to extrapolate information on the repetitive regions endogenously present in the human genome. To assess this, we searched for putative sgRNA target sites present at >500 copies in a specific chromosome, and identified targetable centromere-external repeats on 18/23 human chromosomes (**new Figure 7b,c**). More complete information is presented in the new **Figure 7** and **Figure EV5** and all accompanying sgRNA information has been provided in Table EV 1.

Figure 7b-c:

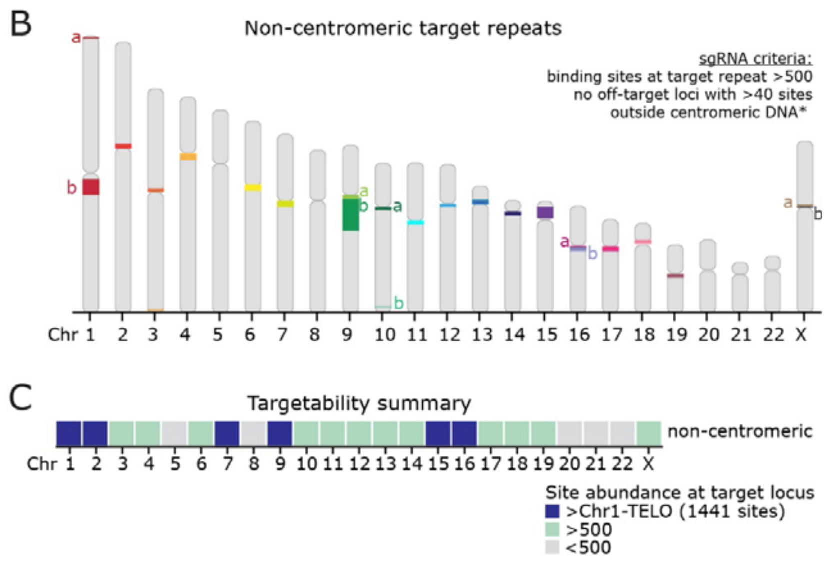

2) I completely missed the reason why the authors did not score mis-segregations from live-cell movies, as those could be seen both in presented movie and in Figure 3E. Authors could exclude the cells that did not enter anaphase during the imaging time and score only cells that entered anaphase. Alternatively, the authors should present clear reasons why they omitted this analysis.

> We apologise for any confusion regarding this point. The live cell imaging presented in the initial manuscript was in the absence of Mps1 inhibitor treatment. Thus most cells arrested in metaphase for the duration of the movie. Those cells that did exit metaphase and proceed through anaphase did exhibit mis-segregating chromatin as noted by the reviewer. However, this was not frequent enough to quantify error rates, hence our decision to use Mps1 inhibitors to score the impact of ectopic kinetochore targeting on mis-segregation frequencies.

However, we have now performed a set of live imaging experiments using Mps1 inhibitor pulses to complement the fixed cell imaging we originally presented. Imaging mis-segregation rates after a brief pulse of Mps1 using live cell imaging is technically challenging; ectopic KT containing cells had to be identified and points set prior to addition of Mps1i, and followed by continued imaging of the same points. However we have been able to analyse the impact of target chromosome mis-segregation in ≥25 cells per condition from ≥ 3 movies, for both Chr1-TELO and Chr9-CEN, and these new data are now presented in **Figure 5g-i (shown below)** as well as more detail in new **Figure EV3**.

Figure 5g-i:

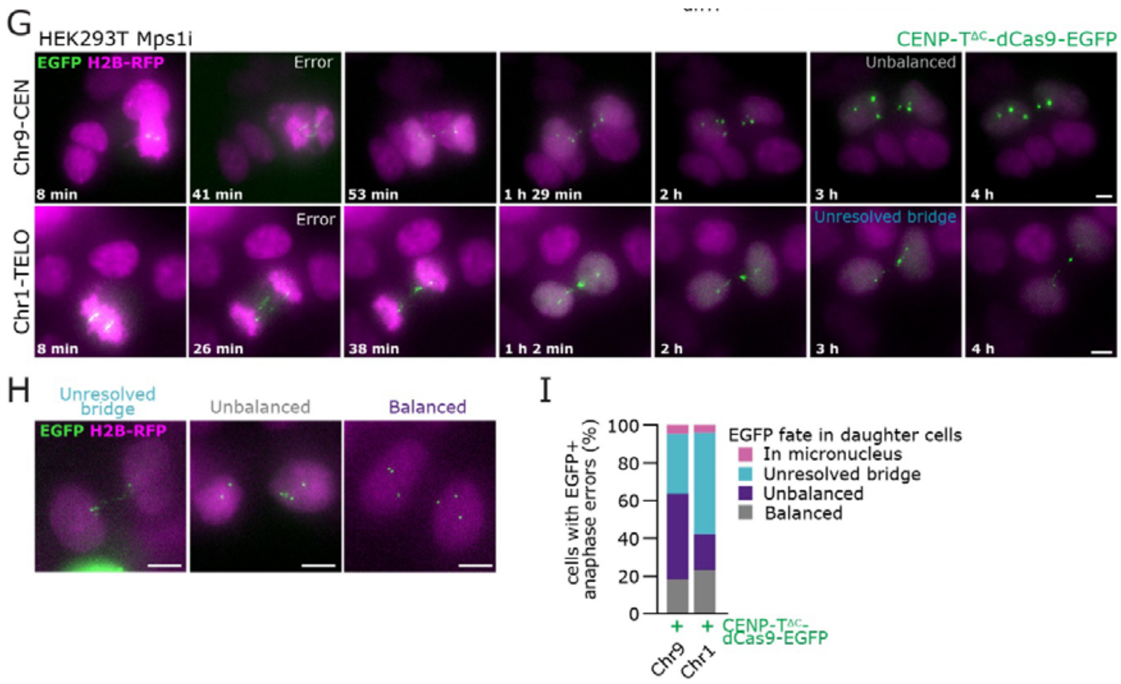

3) Data presented on Figure 4E is really puzzling to me. First, overall chromosome mis-segregation rate is lower in Mps1-treated then in untreated cells. I do not know of any paper that reported similar phenotype, and this should be discussed. Furthermore, the authors should comment on the observed base difference between mis-segregation rates of chromosome 9 and chromosome 1 in HEK cells upon Mps1 inhibition. Chromosome 9 seems to mis-segregate much more often than chromosome 1 in the case of dCas9-EGFP Mps1-treated cells, but they mis-segregate to a similar degree in CENP-TDC-dCas9-EGFP Mps1-treated cells. The observation that chromosome 9 mis-segregates more in HEK dCas9-EGFP cells treated with Mps1 is at odds with the similar data presented on Figure 5 where frequency of non-clonal CNAs per cell is much more prominent for chromosome 1 then for chromosome 9 in dCas9 and no guide conditions. Also, it is puzzling how Mps1 inhibition did not increased mis-segregations of EGP-chromosomes after Mps1 inhibition in HEK cells, because it expectedly managed to do so in HCT cells. Authors should discuss these issues.

> To improve the robustness of this analysis and address the issues the reviewer (and other reviewers) noted, we have repeated these experiments and increased cell numbers for the HEK293 cell line which indeed originally gave slightly confusing results. We have also included data on the untransfected cells with and without Mps1 treatment for both HCT116 and HEK293 cells. We have also now re-presented this data to indicate cells with EGFP-negative only, EGFP+ only or both EGFP+ and EGFP-negative chromosomes mis-segregating. With increased cell numbers and experiments analysed, and clearer presentation we hope it is now more evident that indeed Mps1 inhibition *per se* elevates segregation errors. We have also included a direct comparison of error rates in untreated vs Mps1i treated cells in **Figure EV2f-g** (also shown below) as part of our Mps1i titration experiments.

Figure 5e-f:

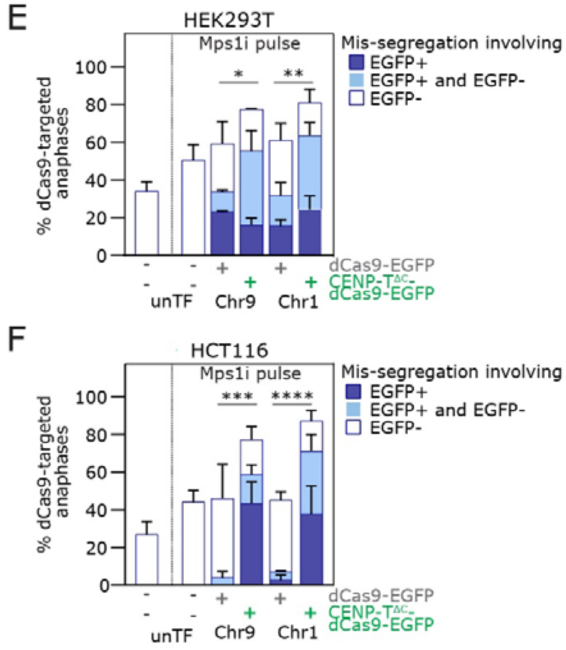

Figure EV2f-g:

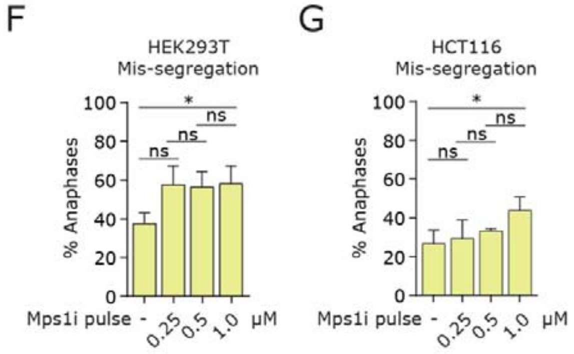

In terms of the base difference between dCas9-EGFP controls for chromosome 9 and 1, with more cells analysed and the outlier experiment replaced there is no longer a large difference in basal mis-segregation rates between C1 and C9 targeted cells in dCas9-EGFP conditions.

Resolving any potential discrepancies between chromosome 9 and 1 in mis-segregation rates and aneuploidy in the form of non-clonal CNAs is somewhat complicated by the fact that it is not possible to entirely remove all putative subclonal events from the SCS data without losing real events. To better account for this we have performed a reanalysis of the sequencing data to improve the efficiency of subclonal CNA removal: We now remove any subclonal CNA seen in any condition in which the guide RNA tested is not present eg. for Chr1 conditions we used 1) untransfected, 2) dCas9 Chr9, and 3) CENPT Chr9 conditions. This gives a slightly clearer analysis which we also explain in more detail in the text:

*"ClonalMasker[11] (see methods) was used to remove clonal and subclonal aneuploidies (present in identical positions across more than one cell) present in the relevant non-targeted controls from each additional condition, and report back only unique aneuploidies (see pileups in **Figure EV4b,c**)."*

In our adjusted analysis non-clonal CNA rates for chromosomes 9 and 1 under CENP-T $^{\Delta C}$ targeting are now very similar to one another (**Figure 6d, shown below**), and fit better with the observed ~60% mis-segregation rates in **Figure 5e** when we consider the rate of error resolution after anaphase (**Figure 5i**).

Figure 6d:

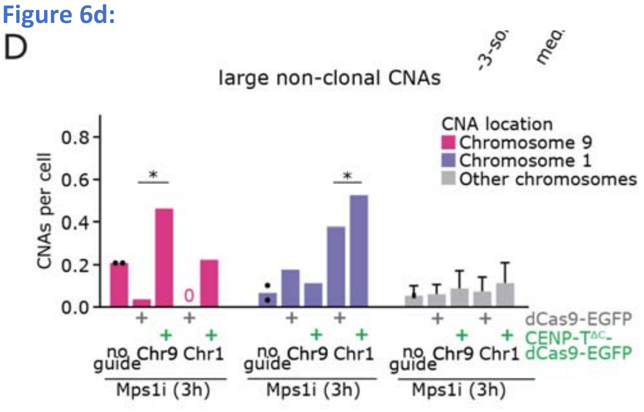

We hope that the reviewer finds the revised and improved data and explanations clearer.

4) The authors should discuss more the puzzling data presented for Chromosome 1 on Figure 5E, and implications of such observations for the usage of Cas9 approaches in chromosome-specific labelling. If a understood the graph correctly, higher aneuploidy score for chromosome 1 is observed even when dCas9-EGFP is targeted to chromosome 9, and to a rather similar degree when dCas9-EGFP is targeted to the chromosome 1, or when CENP-TDC-dCas9-EGFP is targeted on either chromosome 9 or chromosome 1. The possible reasons and implications should be discussed. Also, the authors should cite the work that showed how incomplete replication of the chromosomal repeats could be caused by binding of Cas9 to those repeats during S phase (Whinn et al., 2019, Sci Rep).

> We agree the original analysis and presentation suggested that there were high rates of aneuploidy caused by control conditions. As we stated in the original manuscript, these data are likely confounded by difficulties removing all subclonal CNAs from the data (whilst still retaining likely real dCas9-induced events). Our adjusted sequencing analysis (detailed above for point 3) gave a clearer analysis for Chr9 in particular, and we also now specifically note this in the text:

*"Interestingly the dCas9-EGFP control targeting to chromosome 1 also induced segmental aneuploidies of chromosome 1 although we interpret this result with caution since chromosome 1 exhibited a high frequency of alteration across most conditions likely due to additional sub-clonal aneuploidies present in the parental line (**Figure 6e**)"*

We have also cited the suggested article in the Discussion:

"Lastly, in our conditions, the dCas9 protein binding to repetitive regions during S-phase could have precipitated additional chromosomal alterations, even in the absence of CENP-T$^{\Delta C}$, as previously noted[12], potentially explaining the increased target chromosome mis-segregation rates under this condition."

We hope that the reviewer finds the revised and improved data and explanations clearer.

Minor comments:

1) It would be interesting if authors could measure interkinetochore distances as a proxy for microtubule attachment and tension across ectopic kinetochores in different conditions including dCas9-EGFP, CENP-TDC-dCas9-EGFP, with and without nocodazole, at least in situations where this is possible due to the bar-like and rather fragmented shape of ectopic kinetochores.

> We agree this would be a useful proxy measurement. As it was difficult to accurately measure interkinetochore distances we instead measure *intra*-kinetochore stretching by measuring the length of the EGFP+ focus under different conditions (new **Figure 3a-c also shown below**). Moreover, guided by additional revision experiments, we have now performed a direct assessment of ectopic kinetochore microtubule attachment status, including the simultaneous detection of Mad2 (please see point 2 below).

Figure 3a-c:

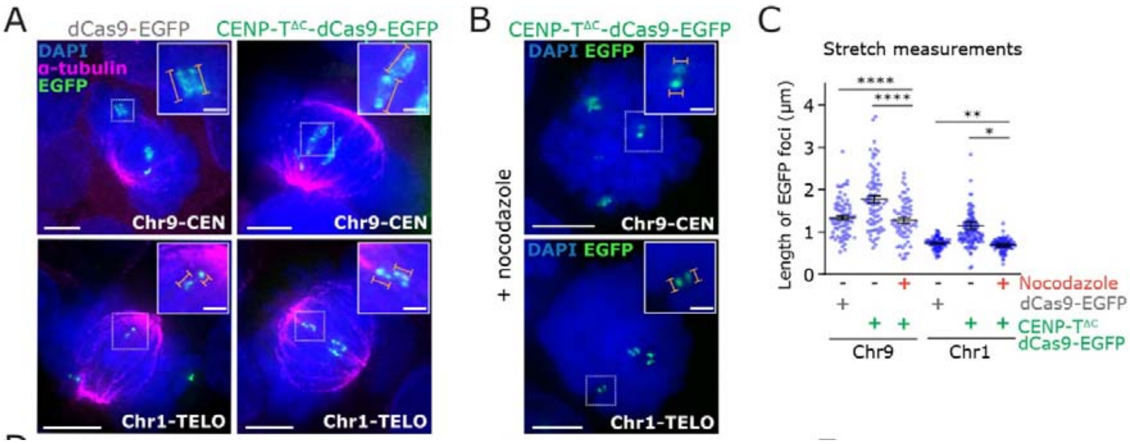

2) As it was shown that SAC response scales with number of unattached kinetochores (Dick&Gerlich, 2013, Nat Cell Biol), it would be interesting for authors to check if substantial mitotic delays are due to the presence of unattached kinetochores by performing Mad labelling of cells with ectopic kinetochores. The authors said in the Discussion part that they '...did not notice the presence of obviously unattached kinetochores herein' but without presenting any data for this claim. Authors should in any way discuss more the possible reasons why would ectopic kinetochores induce substantial mitotic delays, as it is not obvious that every improper attachment would result in SAC activation, as authors state.

> We agree, and have now performed an analysis of ectopic kinetochore attachment status with Mad2 immunofluorescence which revealed an array of attachment statuses including a large proportion of attached ectopic kinetochores with clear Mad2 staining (new **Figure 4**). We have included this as an additional results section, and have expanded in our discussion related to this point:

"In addition, Mad2-positivity status interestingly revealed that ~60% of attached kinetochores retained Mad2. This is a very unusual kinetochore state, though has been observed previously in conditions where Aurora Kinase B is re-localised away from the inner centromere to the kinetochore[13,14]. We favour the possibility that the aberrant Mad2 retention at our ectopic kinetochore stems from an imbalance of kinetochore protein stoichiometry, or loss of normal spatial relationships. For instance, malfunction of Dynein-mediated stripping of Mad2, overloading of KNL-1 as the catalytic platform for MCC formation, or created imbalances in phosphatase/kinase activity could all contribute to the phenotype that we observe. The abnormal Mad2 state observed at dCas9-nucleated ectopic

kinetochores could therefore provide a model system to investigate requirements for proper coupling of Mad2 status to attachment status, and as such provides an ideal platform to study mitotic checkpoint regulation in future studies."

3) The conclusion presented on page 9, line 212, is a bit strange as Aurora B inhibition is known to affect SAC response per se, as the authors nicely point out in the Discussion part of the manuscript (see also Gurden et al., 2018, Oncotarget; Santaguida et al., 2011, EMBO J). I would avoid such unclear conclusions in the Results section.

> We agree that interpreting this result is with caution given Aurora B's roles in the mitotic checkpoint. We also thank the reviewer for the additional references suggestions which are now also included in the Discussion. With the addition of the new analysis of ectopic kinetochore attachment status we observed a notable proportion of attachment types consistent with a role for Aurora B error correction in contributing to the metaphase arrest (see point above). We have now included this new data in the results section (page 9) and moved more speculative statements to the Discussion:

"In addition to canonical mitotic checkpoint signalling from the observed unattached ectopic kinetochores, it is likely that ongoing error correction of erroneous attachments also contributes to mitotic checkpoint activation; Aurora B inhibition partly overcame the mitotic arrest and approximately 30-40% of ectopic kinetochores displayed attachment states (eg. merotelic and syntelic, ***Figure 5b****) normally subject to Aurora B mediated detachment and subsequent checkpoint activation by the resulting unattached kinetochore. Although Aurora B's role in establishment of the mitotic checkpoint per se*[15-17] *means this result should be interpreted with caution, the frequency of unattached ectopic kinetochores observed does not appear to be sufficient to explain the arrest seen without additional SAC activation from other sources."*

4) On page 10, line 222, the authors classified ectopic kinetochore mis-segregation as any event where lagging chromosome appeared in the center of the segregating DNA masses. However, due to usage of fixed cell analysis, authors cannot tell if such lagging structures would resolve later during anaphase, as shown nicely by a series of recent publications implicating Aurora B in error correction of lagging chromosomes during early anaphase (Orr et al., 2021, Curr Biol; Sen et al., 2021, Dev Cell).

> This is a very good point, and we agree that the lagging rate is not a direct read out of mis-segregation or aneuploidy. To assess this, we followed cells using live cell imaging for 4 hours after an Mps1i pulse and scored whether daughter pairs displayed evidence of mis-segregation. Since it was not always possible to fully resolve individual foci we scored whether cells displayed 'balanced' or 'unbalanced' distribution of GFP foci. We observed that the majority of mis-segregating EGFP-positive chromatin generated daughter cells with unbalanced EGFP focus distribution, or unresolved EGFP-positive bridges (new **Figure 5g-i, also shown below**).

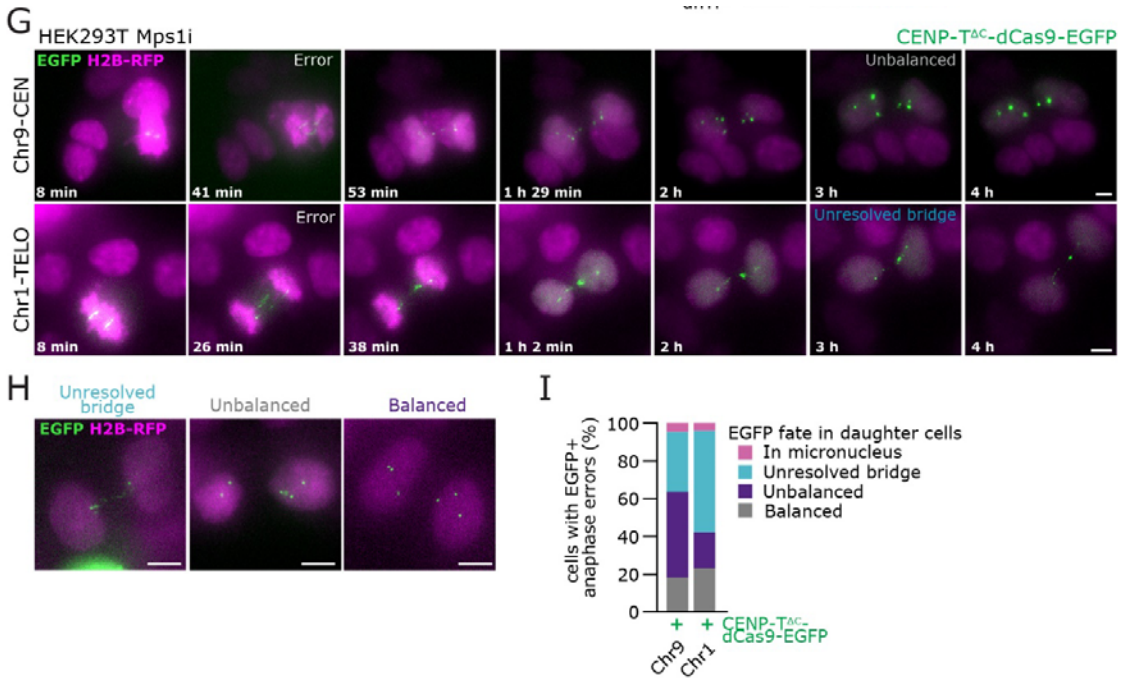

5) The authors should calculate and comment on statistical significance between data presented on Figures 5C and 5E.

> We thank the reviewer for pointing this out. Since this was a single experiment we were constrained to using a Chi^2 test comparing a frequency table of the number of cells vs number of CNAs. When comparing dCas9 vs CENP-T$^{\Delta C}$ dCas9 conditions, we found both to be statistically significant at $p < 0.05$ as has now been added to both the graph (**Figure 6d**) and text.

*"Nonetheless, with CENP-T$^{\Delta C}$ targeting to chromosome 1 and 9 we were able to detect a statistically significant enrichment in partial aneuploidies affecting the target chromosomes (**Figure 6d**)."*

6) One of the main predications of the study is that generation of ectopic kinetochores would induce erroneous kinetochore microtubule attachments, but the authors do not present any evidence that attachments on ectopic kinetochores are indeed erroneous during mitosis. Authors should at least discuss why would such kinetochores be more prone to formation of erroneous attachments, and what types of attachments they expect.

> We have now included a new analysis of ectopic kinetochore attachment status in combination with Mad2 detection (new **Figure 4**; see point above) which clearly supports our conclusion that ectopic kinetochores are prone to erroneous attachments.

7) Authors should acknowledge that whole chromosome aneuploidies are rarely observed in this study, and emphasize more the limits of Mps1 inhibitor usage in the Discussion part of the manuscript.

> We agree with the reviewer and we now include a "Limitations of the current approach" paragraph in the Discussion:

"We also note some limitations of our approach. The major outcome of the induced segregation errors was segmental aneuploidies, rather than whole chromosome aneuploidies, potentially adding complexity to the study of downstream consequences of induced specific chromosome instability events. Additionally, a brief pulse of Mps1 inhibition was required to overcome the mitotic arrest. Since Mps1 inhibition itself can induce chromosome segregation errors this treatment elevated background mis-segregation. We note, however, that this treatment

caused only a moderate increase over the basal error rate in both the cell lines tested. Although only a low rate of micronucleus formation was observed, the majority of mis-segregating target chromosomes led to chromatin bridges that persisted for several hours. Further study is therefore required to determine their ultimate fate, however we anticipate this could be a useful model to study the breakage of chromatin bridges, a common feature in cancer cells. Lastly, in our conditions, the dCas9 protein binding to repetitive regions during S-phase could have precipitated additional chromosomal alterations, even in the absence of CENP-T$^{\Delta C}$, as previously noted[12], potentially explaining the increased target chromosome mis-segregation rates under this condition."

8) I would suggest the authors to change the manuscript title to one that is more specific, reflecting the exact approach that was used.
> We agree, and have changed to title of the manuscript to a more suitable one that better reflects the generation of sub-chromosomal aneuploidies.

Additional comments:
9) On Figure 1C in the dCas9-GFP subpanel I see 4 dots of EGFP signal in the inset, but in CENP-TDC I see only two dots, and in EGFP panel I can see multiple CENP-T signals of varying intensities. Could authors explain better the number of dots of CENP-T we should expect to see?
> We apologise for the lack of clarity for the dots number in the figures, this is because variable z-stack projections are provided depending on what was being visualised. We have now provided additional comments in the figure legends to clarify this.

10) On page 7, line 166, the authors say that 'Such fibers are rarely observed in EGFP-negative...', but do not give any data on this claim. Authors should present IF data on this or delete this sentence.
> The sentence has now been deleted.

11) In the caption for Figure 4E and F authors state that 'red line = mean error rate in untreated cells' but it is unclear to me from where they acquired these numbers, as they are not presented in the manuscript.
> We have provided the untreated error rates in full in new **Figure 5e,f**:

E

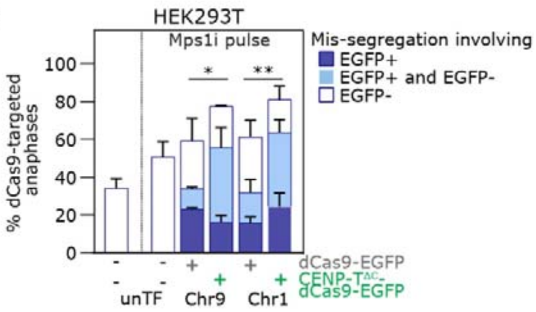

F

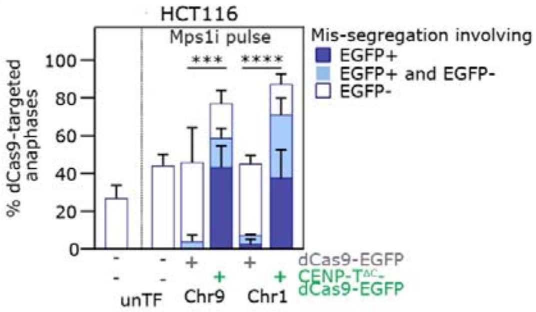

12) On page 10, line 230, the authors state that: 'This was true for both chromosome 9 and 1, and in both HEK293T and HCT116 cells'. However, chromosome 1 data in HEK cells is not significant when comparing dCas9-EGFP and CENP-TDC-dCas9-EGFP MPS1-treated cells. Authors should also discuss more the possible reasons for differences observed between HEK and HCT cells.

> Following the addition of additional repeat analyses and removal of one outlier experiment, these comparisons are now all significant (please see **Figure 5e,f** above).

13) Are the microscopy images presented in figures maximal projections or a single z-planes?

> We apologise for the lack of image information. The number of z-stacks shown in each figure are now included in the legends for the microscopy figures.

14) I recommend that the authors label the q and p arms, and gains and losses on the CAN pileups to ease their reading.

> We agree this could be helpful however this figure became too complex to add these labels.

15) Color shades presented in CAN pileups are explained only in Figure S3, and not in Figure 5.

> We thank the reviewer for pointing this out and have now included the colour codes in both figures (**now Figure 6 and EV4**), as well as clarification in the legend of Figure 6 as to which panels the key refers.

16) The authors should speculate more on reasons why ectopic kinetochores do not assemble on ectopic site when smallest array of MUC4 locus on chromosome 3 was used, as it is currently unclear.

> A speculation on these results is now included in the Discussion:

"Reasons why near-endogenous levels of ectopic CENP-T$^{\Delta C}$ were insufficient to recruit KNL1 are not clear but could include the lack of an endogenous CENP-A-CENP-C nucleation pathway to support the CENP-T$^{\Delta C}$ kinetochore nucleation. This would imply a potential synergy between these two pathways that could have been obscured by the very high binding levels of ectopic CENP-T and CENP-C in previous studies. Nonetheless, it is surprising that KNL1 was essentially undetectable at these MUC4-targeted CENP-T$^{\Delta C}$, potentially suggesting non-linear loading of

KNL1 to ectopic CENP-T$^{\Delta C}$. Proper phospho-regulation of the ectopic CENP-T may also be dysregulated which could impair efficient KNL1 loading (until the point at which this can be overcome by overloading CENP-T). Further investigation into the phosphorylation state of proteins at ectopic kinetochores would be very interesting and may also shed light on the incorrect regulation of Mad2 removal (see below).”

Response to Reviewer 3

Recurrent aneuploidy patterns have been observed in various cancer types. The origin of these aneuploidy patterns and how do they evolve remains unknown. The challenge has been the lack of a strategy to induce specific chromosome aneuploidies in specific cell types so that the immediate cellular responses can be evaluated and eventually the later adaptive responses during clonal expansion examined. The approaches available so far are limited because either rely on clonal expansion upon an initial karyotype change or generate karyotype changes randomly. In this study, Tovini et al. describe a novel strategy to mis-segregate specific chromosomes in different human cell lines. The strategy consists on targeting the kinetochore-nucleating domain of centromere protein CENP-T to assemble ectopic kinetochores in chromosome-specific endogenous repeats (chromosomes 1, 3 and 9). Ectopic kinetochores in chromosomes 1 and 9 induced elevated mis-segregation and aneuploidy on the targeted chromosomes. The strategy still faces limitations but provides an exciting basis for further improvement. The mitotic checkpoint activation by the ectopic kinetochores implies the use of checkpoint inhibitors which induce mis-segregation of non-targeted chromosomes. Moreover, the strategy primarily induces segmental vs. whole chromosomal aneuploidies, which might lead to different cellular responses. Nevertheless, an important step towards the generation of specific aneuploidies was given, using elegant challenging methodologies. I am supportive of the publication provided the authors address the concerns below.
> We thank the reviewer for the positive overall comments and for supporting its publication.

Major comments

1. The title should perhaps be more specific, e.g., 'Targeted assembly of ectopic kinetochores to induce chromosome-specific segmental aneuploidies in human cells'.
> We agree with the reviewer, and appreciate the title suggestion which we have used.

2. I reviewed the related manuscript by Truong et al., also submitted to this journal, in which a motor-based approach is used to induce chromosome-specific mis-segregations. I consider that both manuscripts, while under a common goal and similar experimental approaches, provide complementary data.
> We thank the reviewer for positively supporting both manuscripts.

2.1. Here, Tovini et al. provide new insights into ectopic kinetochore biology, namely on the size and position of the target array required for ectopic kinetochore formation. Details of guide RNAs used to target 3 chromosomes and their binding sites are adequately described in Table 1. An approximate threshold of >1441 guide RNA binding sites required to assemble CENP-T-nucleated ectopic KTs could be inferred. It would be useful if the authors could extend their analysis into other chromosomes exhibiting targetable endogenous repeats (localization and size) to infer the extrapolation of the strategy to other chromosomes.
> We agree with the reviewer that it would be complete to provide an extended analysis on other possible target sites. The recent release of the T2T assembly[10] allowed us to extrapolate information on the repetitive regions endogenously present in the human genome. To assess this, we searched for

putative sgRNA target sites present at >500 copies in a specific chromosome, and identified targetable centromere-external repeats on 18/23 human chromosomes (**new Figure 7b,c**). More complete information is presented in the new Figure 7 and all accompanying sgRNA information has been provided in **Table EV1**.

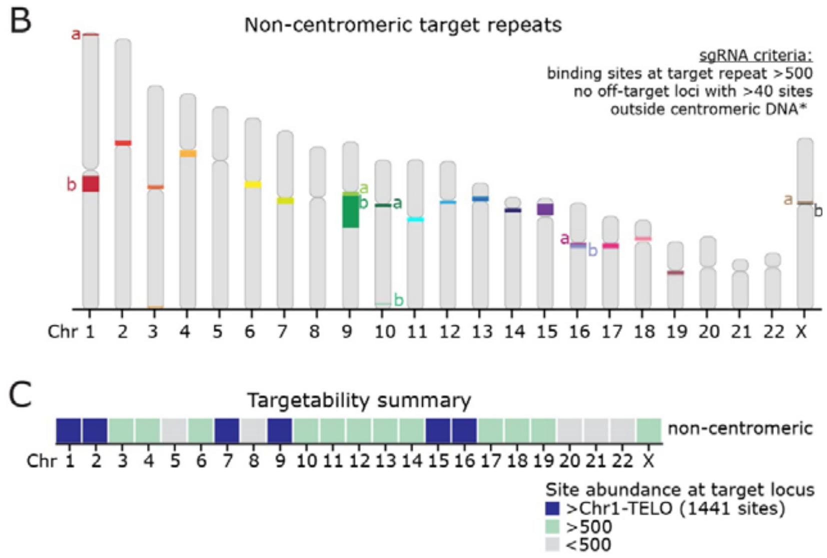

2.2. CENP-TdC-nucleated KTs were shown to induce the mitotic spindle assembly checkpoint - mitotic delay via Aurora B activity. Could the authors include (similarly to Truong et al.) any immunostaining for a checkpoint protein?

> We now include data on Mad2 staining and attachment status as a new results section (new **Figure 4**), showing recruitment of Mad2 to a large proportion of ectopic kinetochores (which comprise a range of attachment types, see new **Figure 4**), in line with the mitotic arrest observed.

2.3. The mitotic delay induced by CENP-TdC-nucleated KTs is disadvantageous in comparison to the motor-based approach described by Truong et al. since it compels the use of checkpoint inhibitors to induce chromosome mis-segregation. The authors circumvented the mitotic arrest by using pulses of Mps1i and AurkBi. Whereas mis-segregation of non-EGFP (non-targeted) chromosomes was not apparent in HEK293T cells (but these cells have high error rate in untreated controls, Fig.4E), it was significant in HCT116 cells despite their lower error rate (Fig.4F). Still, dCas9-EGFP-CENP-TdC induced mis-segregation of EGFP+ chromosomes much more than non-EGFP chromosomes and vs. dCas9-EGFP control.

> We agree that ideally no metaphase arrest would be caused for induction of aneuploidy, and this is now discussed in a new section of the Discussion "Limitations of the current approach". Though, as the reviewer points out, we agree that, despite some background errors, the major impact is mis-segregation of the target chromosome. In order to mitigate against the impact of Mps1i on background (non-target) mis-segregation, we attempted to titrate Mps1 inhibitor concentrations, however this failed to significantly reduce errors rates and furthermore was not capable of a full mitotic arrest release (**Figure EV2f-h**, also shown below) . However we note that Mps1i caused only a moderate increase over the basal error rate in both the cell lines tested:

Figure EV2f-h:

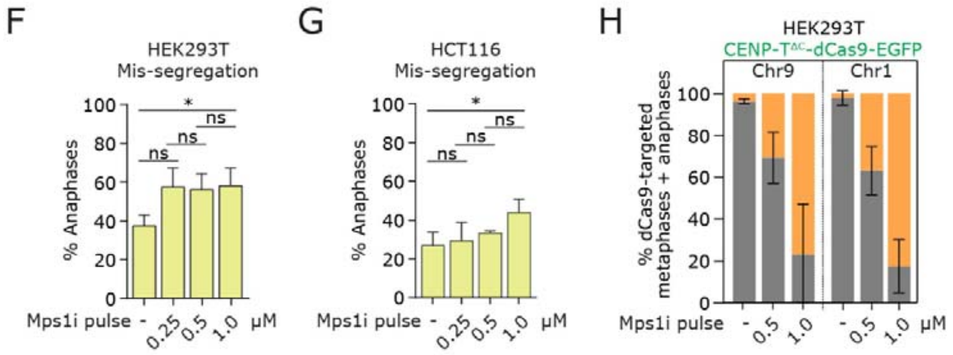

2.3.1. Did the authors do a similar analysis to Fig. 4F but using the AurkBi (that worked efficiently in HCT116 cells, Fig. 4B)? Perhaps AurkBi would induce less mis-segregation events in non-EGFP chromosomes.

> We thank the reviewer for the comment, and agree with the possibility that AurkBi would result in a lower chromosome mis-segregation rate. We attempted to analyse segregation errors under this condition, however due to the major impact on anaphase caused by Aurora B inhibition we were not able to determine error rates from these anaphase cells.

2.3.2. Why did the authors use HEK293T Mps1i for single-cell sequencing if the HCT116 Mps1i (or AurkBi) appear to exhibit better EGFP+ mis-segregation (induction of Chr1 mis-segregation is not striking in HEK293T cells (Fig. 4E), further complicated by the fact that Chr1 exhibited a high frequency of aneuploidies across most conditions, Fig.5A,E)?

> We agree that using HCT116 cells for the single cell sequencing of daughters would have been informative from this point of view. Our ultimate choice to use HEK293 cells for this analysis was driven by the fact that the transfection efficiency of this cell line is much higher than in HCT116 cells, therefore increasing the probability of having sufficient numbers of cells subjected to targeted mis-segregation to allow detection by (very expensive) single cell sequencing of the whole transfected population. In the future we hope to be able to improve the system to allow its application and assessment of aneuploidy in near-diploid or diploid lines to address this issue.

2.4. Here, Tovini et al. provide deeper analysis of breakpoints enrichment in chromosomes 1 and 9 in comparison to Truong et al. (elegant Figs.4B,D), although Truong et al. motor-based strategy appeared to work more efficiently in the induction of mis-segregation events (perhaps because RPE1 cells were used in single-cell sequencing analysis). This shows that the position of target array can dictate the arm-level aneuploidy generation. It is interesting that ectopic kinetochore assembly worked better in Chr9-cen vs. Chr1-tel (Tovini et al.), whereas motor-based approach worked better in Chr1-tel vs. Chr9-cen (Truong et al.) (dCas9-GFP control also induced Chr9 mis-segregation in this study).

> This is an interesting point, that we decided to include in our Discussion:

"Customising the location of ectopic kinetochore formation would therefore theoretically allow the modelling of specific cancer-associated events, such as arm-level aneuploidy generation, improper kinetochore-microtubule attachments, and dicentric chromosomes."

3. The authors should tone down all over the manuscript that the strategy tested induced segmental (not whole) chromosome-specific aneuploidies.

> We agree with the reviewer and we carefully revised the wording of the manuscript to reflect this point.

4. The authors mention in the discussion, the generation of stable cell lines with the capacity to create targeted ectopic KTs at any locus. But as described in Truong et al. clonal expansion to derive the dCas9-Kin14VIb cell line ended up generating an X trisomy. This somehow limits the efficacy of the strategy to study immediate effects of induced chromosome-specific aneuploidies. How do the authors envision to circumvent this?
> This is a good point. When generating stable cell lines we tend to isolate and characterize several clones to counteract potential clone-clone variability. Our primary idea to circumvent this potential issue would be to screen several resulting clones for any newly acquired stable aneuploidies.

5. Since the strategy described ends up requiring the use of Mps1i, the authors should argue about the advantage of dCas9-EGFP-CENP-TdC targeting + Mps1i pulse to study the immediate consequences of specific chromosome aneuploidies vs. a strategy using dCas9-EGFP (only for specific chromosome visualization) + Mps1i (Mps1i has been used to generate karyotype changes randomly that could be tagged if using dCas9-EGFP targeting to chromosome-specific repeats). Would this facilitate the induction of whole vs. segmental specific aneuplodies?
> We appreciate the constructive comment of the reviewer. We agree that using the dCas9-GFP as a tracking system for randomly generated aneuploid cells analysis could indeed be a useful tool. Our primary aim here was to create a population enriched for a specific chromosomal alteration to facilitate downstream assays that could not be performed on single cells. However, we have also had many thoughts about tracking aneuploidy events in single cells. Our new analysis of repetitive regions, coupled to the recent preprint from the Davoli lab providing centromeric repeats[18], provides a wealth of opportunity for such approaches and we hope to capitalize on this in the future.

1 Leibowitz, M. L. *et al.* Chromothripsis as an on-target consequence of CRISPR-Cas9 genome editing. *Nature genetics* **53**, 895-905, doi:10.1038/s41588-021-00838-7 (2021).

2 Girish, V. *et al.* Oncogene-like addiction to aneuploidy in human cancers. *bioRxiv*, 2023.2001.2009.523344, doi:10.1101/2023.01.09.523344 (2023).

3 Gisselsson, D. *et al.* Chromosomal breakage-fusion-bridge events cause genetic intratumor heterogeneity. *Proceedings of the National Academy of Sciences* **97**, 5357-5362, doi:doi:10.1073/pnas.090013497 (2000).

4 Mackinnon, R. N. & Campbell, L. J. The role of dicentric chromosome formation and secondary centromere deletion in the evolution of myeloid malignancy. *Genet Res Int* **2011**, 643628, doi:10.4061/2011/643628 (2011).

5 Cimini, D. *et al.* Merotelic Kinetochore Orientation Is a Major Mechanism of Aneuploidy in Mitotic Mammalian Tissue Cells. *Journal of Cell Biology* **153**, 517-528, doi:10.1083/jcb.153.3.517 (2001).

6 Thompson, S. L. & Compton, D. A. Examining the link between chromosomal instability and aneuploidy in human cells. *The Journal of cell biology* **180**, 665-672, doi:10.1083/jcb.200712029 (2008).

7 Gascoigne, K. E. *et al.* Induced ectopic kinetochore assembly bypasses the requirement for CENP-A nucleosomes. *Cell* **145**, 410-422, doi:10.1016/j.cell.2011.03.031 (2011).

8 Hori, T., Shang, W. H., Takeuchi, K. & Fukagawa, T. The CCAN recruits CENP-A to the centromere and forms the structural core for kinetochore assembly. *The Journal of cell biology* **200**, 45-60, doi:10.1083/jcb.201210106 (2013).

9 Kuhl, L. M. *et al.* A dCas9-Based System Identifies a Central Role for Ctf19 in Kinetochore-Derived Suppression of Meiotic Recombination. *Genetics* **216**, 395-408, doi:10.1534/genetics.120.303384 (2020).
10 Nurk, S. *et al.* The complete sequence of a human genome. *Science* **376**, 44-53, doi:10.1126/science.abj6987 (2022).
11 Shaikh, N. *et al.* Replication stress generates distinctive landscapes of DNA copy number alterations and chromosome scale losses. *Genome Biol* **23**, 223, doi:10.1186/s13059-022-02781-0 (2022).
12 Whinn, K. S. *et al.* Nuclease dead Cas9 is a programmable roadblock for DNA replication. *Sci Rep* **9**, 13292, doi:10.1038/s41598-019-49837-z (2019).
13 Hayward, D., Roberts, E. & Gruneberg, U. MPS1 localizes to end-on microtubule-attached kinetochores to promote microtubule release. *Current biology : CB* **32**, 5200-5208 e5208, doi:10.1016/j.cub.2022.10.047 (2022).
14 Hengeveld, R. C. C., Vromans, M. J. M., Vleugel, M., Hadders, M. A. & Lens, S. M. A. Inner centromere localization of the CPC maintains centromere cohesion and allows mitotic checkpoint silencing. *Nature communications* **8**, 15542, doi:10.1038/ncomms15542 (2017).
15 Hauf, S. *et al.* The small molecule Hesperadin reveals a role for Aurora B in correcting kinetochore-microtubule attachment and in maintaining the spindle assembly checkpoint. *The Journal of cell biology* **161**, 281-294, doi:10.1083/jcb.200208092 (2003).
16 Gurden, M. D., Anderhub, S. J., Faisal, A. & Linardopoulos, S. Aurora B prevents premature removal of spindle assembly checkpoint proteins from the kinetochore: A key role for Aurora B in mitosis. *Oncotarget* **9**, 19525-19542, doi:10.18632/oncotarget.10657 (2018).
17 Santaguida, S., Vernieri, C., Villa, F., Ciliberto, A. & Musacchio, A. Evidence that Aurora B is implicated in spindle checkpoint signalling independently of error correction. *The EMBO journal* **30**, 1508-1519, doi:10.1038/emboj.2011.70 (2011).
18 Bosco, N. *et al.* KaryoCreate: a new CRISPR-based technology to generate chromosome-specific aneuploidy by targeting human centromeres. *bioRxiv*, 2022.2009.2027.509580, doi:10.1101/2022.09.27.509580 (2022).

Dr. Sarah E McClelland
Barts Cancer Institute
Centre for Cancer Genomics and Computational Biology
Queen Mary, University of London
London EC1M 6BQ
United Kingdom

28th Feb 2023

Re: EMBOJ-2022-111587R
Targeted assembly of ectopic kinetochores to induce chromosome-specific segmental aneuploidies

Dear Sarah,

Thank you again for submitting your revised manuscript. Two of the original referees have now re-assessed it, and given that they are both largely satisfied with the revisions, we shall be happy to accept the study for EMBO Journal publication, following incorporation of a few presentational changes requested by referee 2 in their below-copied re-review.

At this stage, please also address the following still open editorial points:

- Pre-acceptance checks by our data editors have raised several queries with the data descriptors in the figure legends, which you will find as comments in the attached edited/commented Word document with activated "Track changes" option. I would appreciate if you incorporated the requested final text modifications and answered the Figure legend queries directly in this version (and modified figures where necessary), uploading the edited main text document upon resubmission with changes/additions still highlighted via the "Track changes" option, to facilitate our final checking.

- Please adjust the format of the reference list and of the in-text citations according to EMBO Journal format (alphabetical order, author name et al + year, up to 10 authors listed before 'et al' in the bibliography)

- Important: please complete the information in the Data Availability section, including at this stage a direct link to the deposited datasets and making sure that they are now publicly available.

- On the abstract page of the manuscript, please include 4-5 general keyword terms to enhance searchability.

- As we are switching from a free-text author contribution statement towards a more formal statement based on Contributor Role Taxonomy (CRediT) terms, please remove the present Author Contribution section and instead specify each author's contribution(s) directly in the Author Information page of our submission system during upload of the final manuscript. See https://casrai.org/credit/ for more information.

- Please add a brief title and legend for Dataset EV1 in a new, separate tab of the XLSX file. Also, please check the text whether various citations to a non-uploaded Table 1 should actually refer to Dataset EV1

- Please check figure panel references in the text for completeness and correct order of callout; e.g. callout for Figure 1B should be after 1A, 5C after 5B; 6G after 6F, EV2E after EV2D; and callout for Figure EV2E appears to be missing.

- Please cut the EV movie legends from the main text, instead placing each one into one separate legend text file per EV movie; then move each legend file together with the respective movie file into a separate ZIP archive before re-uploading as "Movie EV1/2"

- In Fig EV1C, the dashed highlight boxes are slightly offset from the areas in the zoom box images. Please adjust accordingly. In Fig EV1E, please add dashed highlight boxes indicating from where the zoomed images were taken.

- Finally, please provide suggestions for a short 'blurb' text prefacing and summing up the conceptual aspect of the study in two sentences (max. 250 characters), followed by 3-5 one-sentence 'bullet points' with brief factual statements of key results of the paper; they will form the basis of an editor-written 'Synopsis' accompanying the online version of the article. Please also upload a synopsis image, which can be used as a "visual title" for the synopsis section of your paper. The image should be in PNG or JPG format, and please make sure that it remains in the modest dimensions of (exactly) 550 pixels wide and 300-600 pixels high.

I am therefore returning the manuscript to you for a final round of minor revision, to allow you to make these adjustments and upload all modified files. Once we will have received them, we should be ready to swiftly proceed with formal acceptance and

production of the manuscript.

With kind regards,

Hartmut

Hartmut Vodermaier, PhD
Senior Editor, The EMBO Journal
h.vodermaier@embojournal.org

*** PLEASE NOTE: All revised manuscript are subject to initial checks for completeness and adherence to our formatting guidelines. Revisions may be returned to the authors and delayed in their editorial re-evaluation if they fail to comply to the following requirements (see also our Guide to Authors for further information):

1) Every manuscript requires a Data Availability section (even if only stating that no deposited datasets are included). Primary datasets or computer code produced in the current study have to be deposited in appropriate public repositories prior to resubmission, and reviewer access details provided in case that public access is not yet allowed. Further information: embopress.org/page/journal/14602075/authorguide#dataavailability

2) Each figure legend must specify
- size of the scale bars that are mandatory for all micrograph panels
- the statistical test used to generate error bars and P-values
- the type error bars (e.g., S.E.M., S.D.)
- the number (n) and nature (biological or technical replicate) of independent experiments underlying each data point
- Figures may not include error bars for experiments with n<3; scatter plots showing individual data points should be used instead.

3) Revised manuscript text (including main tables, and figure legends for main and EV figures) has to be submitted as editable text file (e.g., .docx format). We encourage highlighting of changes (e.g., via text color) for the referees' reference.

4) Each main and each Expanded View (EV) figure should be uploaded as individual production-quality files (preferably in .eps, .tif, .jpg formats). For suggestions on figure preparation/layout, please refer to our Figure Preparation Guidelines: http://bit.ly/EMBOPressFigurePreparationGuideline

5) Point-by-point response letters should include the original referee comments in full together with your detailed responses to them (and to specific editor requests if applicable), and also be uploaded as editable (e.g., .docx) text files.

6) Please complete our Author Checklist, and make sure that information entered into the checklist is also reflected in the manuscript; the checklist will be available to readers as part of the Review Process File. A download link is found at the top of our Guide to Authors: embopress.org/page/journal/14602075/authorguide

7) All authors listed as (co-)corresponding need to deposit, in their respective author profiles in our submission system, a unique ORCiD identifier linked to their name. Please see our Guide to Authors for detailed instructions.

8) Please note that supplementary information at EMBO Press has been superseded by the 'Expanded View' for inclusion of additional figures, tables, movies or datasets; with up to five EV Figures being typeset and directly accessible in the HTML version of the article. For details and guidance, please refer to: embopress.org/page/journal/14602075/authorguide#expandedview

9) Digital image enhancement is acceptable practice, as long as it accurately represents the original data and conforms to community standards. If a figure has been subjected to significant electronic manipulation, this must be clearly noted in the figure legend and/or the 'Materials and Methods' section. The editors reserve the right to request original versions of figures and the original images that were used to assemble the figure. Finally, we generally encourage uploading of numerical as well as gel/blot image source data; for details see: embopress.org/page/journal/14602075/authorguide#sourcedata

At EMBO Press, we ask authors to provide source data for the main manuscript figures. Our source data coordinator will contact you to discuss which figure panels we would need source data for and will also provide you with helpful tips on how to upload and organize the files.

Further information is available in our Guide For Authors:

https://www.embopress.org/page/journal/14602075/authorguide

In the interest of ensuring the conceptual advance provided by the work, we recommend submitting a revision within 3 months (29th May 2023). Please discuss the revision progress ahead of this time with the editor if you require more time to complete the revisions. Use the link below to submit your revision:

Link Not Available

Referee #1:

The authors have satisfactorily addressed my critiques. I have very much enjoyed reading this revised manuscript and believe it will be an important contribution to the field.

Referee #2:

The revised manuscript now entitled "Targeted assembly of ectopic kinetochores to induce chromosome-specific segmental aneuploidies" by Tovini et al. is, in my opinion, a substantial improvement over the first version of the manuscript originally submitted to the journal. The authors responded to the reviewers' comments by introducing a completely new set of experimental data, of which I personally adored live cell imaging experiments the most, especially since I know how challenging that approach could be. Therefore, I congratulate the authors on taking this path. Also, the perplexing results on Mad2 on ectopic kinetochores, presented by the authors in the revised manuscript, only strengthen the manuscript in my opinion, as they could be useful for studies of checkpoint response, as the authors nicely point out by themselves. Furthermore, the authors have performed an analysis of putative repeat sequences in the human genome for ectopic kinetochore seeding, which could be a valuable resource for a larger community. The authors also reanalyzed the existing data, performed more replicates of the experiments presented earlier to make a clearer picture of the data, reorganized the figure panels, and significantly improved the discussion of the pros and cons of the presented method. Together, the new data and improved figure and text presentations strengthen the main conclusions of the manuscript.

In my opinion, the manuscript presented in this way is a valuable contribution to the field and therefore I am a passionate advocate for the publication of the manuscript. I have only a few minor comments to make to the authors to improve the final clarity of the manuscript.

Minor comments:
1) When discussing the surprisingly low ratio of micronucleation few hours after induction of chromosome mis-segregation via formation of chromosome bridge the authors should acknowledge the work from the Pellman lab that showed micronucleation is a major downstream consequence of chromosome bridge formation and breakage, but only after the division of the daughter cells (Umbreit et al., 2020, Science).
2) When discussing non-random aneuploidies in cancer cell types (Introduction section, first paragraph), the authors should acknowledge the recent work from the Kops lab that showed that the content of naturally occurring micronuclei in various human cancer cell lines is biased toward certain chromosomes and is surprisingly recurrent between different cell types (Klaasen et al., 2022, Nature).
3) I would still like the authors to discuss in more detail the logic that ectopic kinetochores are supposed per se to be more prone to erroneous attachment. I understand that the authors presented nice data on this topic, but the authors could still discuss this issue in the Discussion part of the manuscript. Related, the authors state: "For example, CEN9-targeting might interfere with the endogenous chromosome 9 centromere function, potentially mimicking merotelic attachment, while TELO1-targeting might be more likely to create a canonical pseudo-dicentric chromosome." Could the authors discuss these predictions in a clearer way? Why would near-centromere and near-telomere targeting behave so differently and why would CEN-targeting interfere with centromere function? Also related to this issue, could the authors comment more on the fact that unattached ectopic kinetochores are still aligned at a metaphase plate as this does not happen in control human cells where unattached kinetochores are rarely found in the metaphase plate. Is this due to attachments generated on the native centromeres? Lastly, do the authors observe an increase in erroneous attachments on native kinetochores assembled on centromeres of chromosomes in which ectopic kinetochores were assembled?
4) On page 6 of the manuscript the authors state: "In some cells however, inhibition of Aurora B was insufficient to overcome the metaphase arrest (particularly in HEK293T cells)." I would recommend citing some figures after this sentence.
5) On page 6 of the manuscript, the authors state: "Since the use of Mps1i induces some EGFP-negative errors...". I would recommend citing some figures after this part of the sentence.
6) On page 7, the authors cite Figure EV3c in the following sentence: "We also wondered whether mis-segregated EGFP-positive chromatin might become incorporated into micronuclei, however this was observed only rarely, in fewer than 5% of cells (Figure 5i; Figure EV3c; Movie EV2)". Did the authors intend to cite here EV3d? Additionally, I did not find any citations for EV3e.
7) On page 10, there is a typo, 'that' is written two times in a row: "This suggests that that either the breaks indeed occur externally to the targeted region, or that we are unable to detect breaks within this region for technical reasons."

Response to reviewers for "Targeted assembly of ectopic kinetochores to induce chromosome-specific segmental aneuploidies"

Overview of changes:

We thank all reviewers for their assessment of our manuscript and supportive, constructive comments throughout the review process. We have addressed the newly raised comments on the revised manuscript in full below.

Response to Reviewer 1

The authors have satisfactorily addressed my critiques. I have very much enjoyed reading this revised manuscript and believe it will be an important contribution to the field.

> We thank the reviewer for their supportive comments on our revised manuscript.

Response to Reviewer 2

The revised manuscript now entitled "Targeted assembly of ectopic kinetochores to induce chromosome-specific segmental aneuploidies" by Tovini et al. is, in my opinion, a substantial improvement over the first version of the manuscript originally submitted to the journal. The authors responded to the reviewers' comments by introducing a completely new set of experimental data, of which I personally adored live cell imaging experiments the most, especially since I know how challenging that approach could be. Therefore, I congratulate the authors on taking this path. Also, the perplexing results on Mad2 on ectopic kinetochores, presented by the authors in the revised manuscript, only strengthen the manuscript in my opinion, as they could be useful for studies of checkpoint response, as the authors nicely point out by themselves. Furthermore, the authors have performed an analysis of putative repeat sequences in the human genome for ectopic kinetochore seeding, which could be a valuable resource for a larger community. The authors also reanalyzed the existing data, performed more replicates of the experiments presented earlier to make a clearer picture of the data, reorganized the figure panels, and significantly improved the discussion of the pros and cons of the presented method. Together, the new data and improved figure and text presentations strengthen the main conclusions of the manuscript.

In my opinion, the manuscript presented in this way is a valuable contribution to the field and therefore I am a passionate advocate for the publication of the manuscript. I have only a few minor comments to make to the authors to improve the final clarity of the manuscript.

> We thank the reviewer for their careful assessment of our revised manuscript and the constructive suggestions which we have addressed in full below.

1) When discussing the surprisingly low ratio of micronucleation few hours after induction of chromosome mis-segregation via formation of chromosome bridge the authors should acknowledge the work from the Pellman lab that showed micronucleation is a major downstream consequence of chromosome bridge formation and breakage, but only after the division of the daughter cells (Umbreit et al., 2020, Science).

>We agree this is an important comparison to make and have now discussed this point in the text of the discussion section, citing this work.

"Although only a low rate of micronucleus formation was observed, the majority of mis-segregating target chromosomes led to chromatin bridges that persisted for several hours. Further study is

therefore required to determine their ultimate fate, for example previous work has shown that chromatin material from anaphase bridges can form micronuclei in the second cell division (Umbreit et al., 2020)."

2) When discussing non-random aneuploidies in cancer cell types (Introduction section, first paragraph), the authors should acknowledge the recent work from the Kops lab that showed that the content of naturally occurring micronuclei in various human cancer cell lines is biased toward certain chromosomes and is surprisingly recurrent between different cell types (Klaasen et al., 2022, Nature).

>We appreciate the reviewer's suggestion and agree it is important to acknowledge this and have now made this point in the text of the introduction section.

"Aneuploidy in cancer is also non-random, with individual cancer types exhibiting characteristic aneuploidy and somatic copy number alteration (SCNA) landscapes[3], and displaying non-random chromosome mis-segregation(Klaasen *et al*, 2022)."

3) I would still like the authors to discuss in more detail the logic that ectopic kinetochores are supposed per se to be more prone to erroneous attachment. I understand that the authors presented nice data on this topic, but the authors could still discuss this issue in the Discussion part of the manuscript.

We agree this point was not entirely clear. We did not initially expect the ectopic kinetochores to be prone to improper attachment per se. Rather, we expected that amphitelic attachment at ectopic and endogenous kinetochores could create a situation where a single chromatid is attached by microtubules originating from both spindle poles; one to the endogenous kinetochore, and the other to the ectopic kinetochore. We have adjusted our diagram in Figure 1a, and added a sentence to the introduction to reflect this.

Figure 1a:

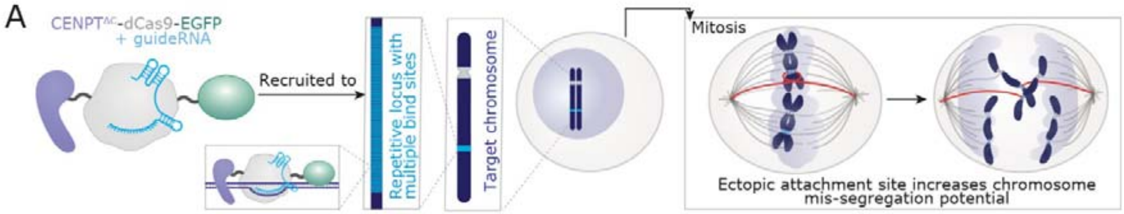

Introduction text:

"This prompted us to test whether dCas9 could also nucleate the formation of functional kinetochores at ectopic loci in human cells, similar to what has been shown in S. cerevisiae (Kuhl et al, 2020). This would create a pseudo-dicentric chromosome that would be more susceptible to mis-segregation due to simultaneous attachment to both centrosomes (Figure 1a)."

Related, the authors state: "For example, CEN9-targeting might interfere with the endogenous chromosome 9 centromere function, potentially mimicking merotelic attachment, while TELO1-targeting might be more likely to create a canonical pseudo-dicentric chromosome." Could the authors discuss these predictions in a clearer way? Why would near-centromere and near-telomere targeting behave so differently and why would CEN-targeting interfere with centromere function?

We decided to remove this speculative statement following our new data showing the ectopic kinetochores seeded on either telomere or centromere proximal loci are prone to improper attachments, seemingly irrelevant of proximity to the endogenous centromere. We have amended the discussion to reflect this:

Discussion text:

*"In terms of positioning, we were able to provoke mis-segregation using both centromere-, and telomere-proximal sites for ectopic kinetochore formation. Initially we had hypothesised that the longer the distance between the native and ectopic kinetochores, the greater the likelihood of that chromosome being mis-segregated, due to a higher probability that a twist in the sister chromatid would allow attachment of the same sister chromatid to both centrosomes (**Figure 1a**). However, we discovered that in fact these ectopic kinetochores were intrinsically prone to improper attachment (see below), regardless of their position relative to the endogenous centromere, meaning that this approach can be used to mis-segregate specific chromosomes independently of the positioning of the ectopic kinetochore."*

Also related to this issue, could the authors comment more on the fact that unattached ectopic kinetochores are still aligned at a metaphase plate as this does not happen in control human cells where unattached kinetochores are rarely found in the metaphase plate. Is this due to attachments generated on the native centromeres? Lastly, do the authors observe an increase in erroneous attachments on native kinetochores assembled on centromeres of chromosomes in which ectopic kinetochores were assembled?

As the reviewer notes, we believe attachments at native centromeres would be sufficient to bring the target chromosome into the metaphase plate, explaining the localisation of unattached ectopic kinetochores here. Consistent with this, in our control condition of targeted dCas9-EGFP, the signal also often localises to the metaphase plate as seen in Figures 1c and 2c,f,i.

Regarding the attachment status at the endogenous centromere, this is a very interesting question, however we are not able to address this here because we did not include a marker for the endogenous centromeres in our analysis due to 4-channel limitations (DAPI, GFP, Mad2, Tubulin).

4) On page 6 of the manuscript the authors state: "In some cells however, inhibition of Aurora B was insufficient to overcome the metaphase arrest (particularly in HEK293T cells)." I would recommend citing some figures after this sentence.

> We have added a citation for Figure 5a,b here.

5) On page 6 of the manuscript, the authors state: "Since the use of Mps1i induces some EGFP-negative errors...". I would recommend citing some figures after this part of the sentence.

> Figures 5e,f and EV2c,d where we report EGFP-negative segregation error rates under Mps1 inhibition have now been cited here.

6) On page 7, the authors cite Figure EV3c in the following sentence: "We also wondered whether

mis-segregated EGFP-positive chromatin might become incorporated into micronuclei, however this was observed only rarely, in fewer than 5% of cells (Figure 5i; Figure EV3c; Movie EV2)". Did the authors intend to cite here EV3d? Additionally, I did not find any citations for EV3e.

> We have now amended this citation to Figure EV3d, and added in a citation for Figure EV3e.

7) On page 10, there is a typo, 'that' is written two times in a row: "This suggests that that either the breaks indeed occur externally to the targeted region, or that we are unable to detect breaks within this region for technical reasons."

> Thank you, this has now been corrected.

Dr. Sarah E McClelland
Barts Cancer Institute
Centre for Cancer Genomics and Computational Biology
Queen Mary, University of London
London EC1M 6BQ
United Kingdom

5th Apr 2023

Re: EMBOJ-2022-111587R1
Targeted assembly of ectopic kinetochores to induce chromosome-specific segmental aneuploidies

Dear Sarah,

Thank you for submitting your final revised manuscript for our consideration. I am pleased to inform you that we have now accepted it for publication in The EMBO Journal.

Your article will be processed for publication in The EMBO Journal by EMBO Press and Wiley, who will contact you with further information regarding production/publication procedures and license requirements. You will also be provided with page proofs after copy-editing and typesetting of main manuscript and expanded view figure files.

Should you be planning a Press Release on your article, please get in contact with embojournal@wiley.com as early as possible, in order to coordinate publication and release dates.

Thank you again for this contribution to The EMBO Journal and congratulations on a successful publication! Please consider us again in the future for your most exciting work.

With kind regards,

Hartmut

Hartmut Vodermaier, PhD
Senior Editor, The EMBO Journal
h.vodermaier@embojournal.org

EMBO Press Author Checklist

Corresponding Author Name: Sarah E McClelland	**USEFUL LINKS FOR COMPLETING THIS FORM**
Journal Submitted to: EMBO Journal	The EMBO Journal - Author Guidelines
Manuscript Number: EMBOJ-2022-111587R	EMBO Reports - Author Guidelines
	Molecular Systems Biology - Author Guidelines
	EMBO Molecular Medicine - Author Guidelines

Reporting Checklist for Life Science Articles (updated January
This checklist is adapted from Materials Design Analysis Reporting (MDAR) Checklist for Authors. MDAR establishes a minimum set of requirements in transparent reporting in the life sciences (see Statement of Task: 10.31222/osf.io/9sm4x). Please follow the journal's guidelines in preparing your
Please note that a copy of this checklist will be published alongside your article.

Abridged guidelines for figures
1. Data
The data shown in figures should satisfy the following conditions:
- the data were obtained and processed according to the field's best practice and are presented to reflect the results of the experiments in an accurate and unbiased manner.
- ideally, figure panels should include only measurements that are directly comparable to each other and obtained with the same assay.
- plots include clearly labeled error bars for independent experiments and sample sizes. Unless justified, error bars should not be shown for technical
- if n<5, the individual data points from each experiment should be plotted. Any statistical test employed should be justified.
- Source Data should be included to report the data underlying figures according to the guidelines set out in the authorship guidelines on Data

2. Captions
Each figure caption should contain the following information, for each panel where they are relevant:
- a specification of the experimental system investigated (eg cell line, species name).
- the assay(s) and method(s) used to carry out the reported observations and measurements.
- an explicit mention of the biological and chemical entity(ies) that are being measured.
- an explicit mention of the biological and chemical entity(ies) that are altered/varied/perturbed in a controlled manner.
- the exact sample size (n) for each experimental group/condition, given as a number, not a range;
- a description of the sample collection allowing the reader to understand whether the samples represent technical or biological replicates (including how many animals, litters, cultures, etc.).
- a statement of how many times the experiment shown was independently replicated in the laboratory.
- definitions of statistical methods and measures:
  - common tests, such as t-test (please specify whether paired vs. unpaired), simple χ2 tests, Wilcoxon and Mann-Whitney tests, can be unambiguously identified by name only, but more complex techniques should be described in the methods section;
  - are tests one-sided or two-sided?
  - are there adjustments for multiple comparisons?
  - exact statistical test results, e.g., P values = x but not P values < x;
  - definition of 'center values' as median or average;
  - definition of error bars as s.d. or s.e.m.

Please complete ALL of the questions below.
Select "Not Applicable" only when the requested information is not relevant for your study.

Materials

Newly Created Materials	Information included in the manuscript?	In which section is the information available? (Reagents and Tools Table, Materials and Methods, Figures, Data Availability Section)
New materials and reagents need to be available; do any restrictions apply?	Yes	Materials and Methods

Antibodies	Information included in the manuscript?	In which section is the information available? (Reagents and Tools Table, Materials and Methods, Figures, Data Availability Section)
For **antibodies** provide the following information:<br>- Commercial antibodies: RRID (if possible) or supplier name, catalogue number and or/clone number<br>- Non-commercial: RRID or citation	Yes	Materials and Methods

DNA and RNA sequences	Information included in the manuscript?	In which section is the information available? (Reagents and Tools Table, Materials and Methods, Figures, Data Availability Section)
**Short novel DNA or RNA including primers, probes**: provide the sequences.	Not Applicable	

Cell materials	Information included in the manuscript?	In which section is the information available? (Reagents and Tools Table, Materials and Methods, Figures, Data Availability Section)
**Cell lines:** Provide species information, strain. Provide accession number in repository **OR** supplier name, catalog number, clone number, and/**OR** RRID.	Yes	Materials and Methods
**Primary cultures:** Provide species, strain, sex of origin, genetic modification status.	Not Applicable	
Report if the cell lines were recently **authenticated** (e.g., by STR profiling) and tested for mycoplasma contamination.	Yes	Materials and Methods

Experimental animals	Information included in the manuscript?	In which section is the information available? (Reagents and Tools Table, Materials and Methods, Figures, Data Availability Section)
**Laboratory animals or Model organisms:** Provide species, strain, sex, age, genetic modification status. Provide accession number in repository **OR** supplier name, catalog number, clone number, **OR** RRID.	Not Applicable	
**Animal observed in or captured from the field:** Provide species, sex, and age where possible.	Not Applicable	
Please detail **housing and husbandry conditions**.	Not Applicable	

Plants and microbes	Information included in the manuscript?	In which section is the information available? (Reagents and Tools Table, Materials and Methods, Figures, Data Availability Section)
**Plants:** provide species and strain, ecotype and cultivar where relevant, unique accession number if available, and source (including location for collected wild specimens).	Not Applicable	
**Microbes:** provide species and strain, unique accession number if available, and source.	Not Applicable	

Human research participants	Information included in the manuscript?	In which section is the information available? (Reagents and Tools Table, Materials and Methods, Figures, Data Availability Section)
If collected and within the bounds of privacy constraints report on age, sex and gender or ethnicity for all study participants.	Not Applicable	

Core facilities	Information included in the manuscript?	In which section is the information available? (Reagents and Tools Table, Materials and Methods, Figures, Data Availability Section)
If your work benefited from core facilities, was their service mentioned in the acknowledgments section?	Yes	Acknowledgments

Design

Study protocol	Information included in the manuscript?	In which section is the information available? (Reagents and Tools Table, Materials and Methods, Figures, Data Availability Section)
If study protocol has been **pre-registered, provide DOI in the manuscript**. For clinical trials, provide the trial registration number **OR** cite DOI.	Not Applicable	
Report the **clinical trial registration number** (at ClinicalTrials.gov or equivalent), where applicable.	Not Applicable	

Laboratory protocol	Information included in the manuscript?	In which section is the information available? (Reagents and Tools Table, Materials and Methods, Figures, Data Availability Section)
Provide DOI OR other citation details if **external detailed step-by-step protocols** are available.	Not Applicable	

Experimental study design and statistics	Information included in the manuscript?	In which section is the information available? (Reagents and Tools Table, Materials and Methods, Figures, Data Availability Section)
Include a statement about **sample size** estimate even if no statistical methods were used.	Yes	Figure legends
Were any steps taken to minimize the effects of subjective bias when allocating animals/samples to treatment (e.g. **randomization procedure**)? If yes, have they been described?	Not Applicable	
Include a statement about **blinding** even if no blinding was done.	Not Applicable	
Describe **inclusion/exclusion criteria** if samples or animals were excluded from the analysis. Were the criteria pre-established?<br><br>If sample or data points were omitted from analysis, report if this was due to attrition or intentional exclusion and provide justification.	Yes	Figure legends
For every figure, are **statistical tests** justified as appropriate? Do the data meet the assumptions of the tests (e.g., normal distribution)? Describe any methods used to assess it. Is there an estimate of variation within each group of data? Is the variance similar between the groups that are being statistically compared?	Yes	Figure legends

Sample definition and in-laboratory replication	Information included in the manuscript?	In which section is the information available? (Reagents and Tools Table, Materials and Methods, Figures, Data Availability Section)
In the figure legends: state number of times the experiment was **replicated** in laboratory.	Yes	Figure legends
In the figure legends: define whether data describe **technical or biological replicates**.	Yes	Figure legends

Ethics

Ethics	Information included in the manuscript?	In which section is the information available? (Reagents and Tools Table, Materials and Methods, Figures, Data Availability Section)
Studies involving **human participants**: State details of **authority granting ethics approval** (IRB or equivalent committee(s), provide reference number for approval.	Not Applicable	
Studies involving **human participants**: Include a statement confirming that **informed consent** was obtained from all subjects and that the experiments conformed to the principles set out in the WMA Declaration of Helsinki and the Department of Health and Human Services Belmont Report.	Not Applicable	
Studies involving **human participants:** For publication of **patient photos**, include a statement confirming that consent to publish was obtained.	Not Applicable	
Studies involving experimental **animals**: State details of **authority granting ethics approval** (IRB or equivalent committee(s), provide reference number for approval. Include a statement of compliance with ethical regulations.	Not Applicable	
Studies involving **specimen and field samples:** State if relevant **permits** obtained, provide details of authority approving study; if none were required, explain why.	Not Applicable	

Dual Use Research of Concern (DURC)	Information included in the manuscript?	In which section is the information available? (Reagents and Tools Table, Materials and Methods, Figures, Data Availability Section)
Could your study fall under dual use research restrictions? Please check biosecurity documents and list of **select agents and toxins** (CDC): https://www.selectagents.gov/sat/list.htm	Not Applicable	
If you used a select agent, is the security level of the lab appropriate and reported in the manuscript?	Not Applicable	
If a study is subject to dual use research of concern regulations, is the name of the **authority granting approval and reference number** for the regulatory approval provided in the manuscript?	Not Applicable	

Reporting

The MDAR framework recommends adoption of discipline-specific guidelines, established and endorsed through community initiatives. Journals have their own policy about requiring specific guidelines and recommendations to complement MDAR.

Adherence to community standards	Information included in the manuscript?	In which section is the information available? (Reagents and Tools Table, Materials and Methods, Figures, Data Availability Section)
State if relevant guidelines or checklists (e.g., **ICMJE, MIBBI, ARRIVE, PRISMA**) have been followed or provided.	Not Applicable	
For **tumor marker prognostic studies,** we recommend that you follow the **REMARK** reporting guidelines (see link list at top right). See author guidelines, under 'Reporting Guidelines'. Please confirm you have followed these guidelines.	Not Applicable	
For **phase II and III randomized controlled trials**, please refer to the **CONSORT** flow diagram (see link list at top right) and submit the CONSORT checklist (see link list at top right) with your submission. See author guidelines, under 'Reporting Guidelines'. Please confirm you have submitted this list.	Not Applicable	

Data Availability

Data availability	Information included in the manuscript?	In which section is the information available? (Reagents and Tools Table, Materials and Methods, Figures, Data Availability Section)
Have **primary datasets** been deposited according to the journal's guidelines (see 'Data Deposition' section) and the respective accession numbers provided in the Data Availability Section?	Yes	Data availability
Were **human clinical and genomic datasets** deposited in a public access-controlled repository in accordance to ethical obligations to the patients and to the applicable consent agreement?	Yes	
Are **computational models** that are central and integral to a study available without restrictions in a machine-readable form? Were the relevant accession numbers or links provided?	Not Applicable	
If publicly available data were reused, provide the respective **data citations in the reference list**.	Not Applicable	
